# Enamel Research: Priorities and Future Directions

**DOI:** 10.3389/fphys.2017.00513

**Published:** 2017-07-20

**Authors:** Jennifer Kirkham, Steven J. Brookes, Thomas G. H. Diekwisch, Henry C. Margolis, Ariane Berdal, Michael J. Hubbard

**Affiliations:** ^1^Division of Oral Biology, School of Dentistry, St James's University Hospital, University of Leeds Leeds, United Kingdom; ^2^Texas A&M University College of Dentistry Dallas, TX, United States; ^3^Center for Biomineralization, The Forsyth Institute Cambridge, MA, United States; ^4^Centre de Recherche des Cordeliers, INSERM UMRS 1138, University Paris-Diderot Paris, France; ^5^Department of Paediatrics, University of Melbourne Melbourne, VIC, Australia

**Keywords:** enamel, research priorities, amelogenesis, odontogenesis, future directions

At the close of the 2016 Enamel 9 International Symposium, the collective wisdom and views of enamel researchers were sought and discussions held in order to provide a consensus view of the priorities and future directions in enamel research. Researchers considered progress in the field since the Enamel VIII conference held in 2011, together with the research strengths and strategic gaps in our current knowledge portfolio, synthesizing this information toward recommendations for future advancement. We aim to present these recommendations and identified priorities here, drawing upon the Closing Session discussions and those held throughout the Symposium as presented in the Supplemental data.

It is clear that significant advancements have been made in enamel research over the last 5 years since Enamel VIII. Despite these advances, it is equally clear that many issues highlighted there as future priorities (Scientific Advisory Board, 2011) are still pressing. The greater our technical capabilities and knowledge base, the more questions and unknowns we uncover and the more we realize what we do not know. More than 100 researchers from 28 countries attended Enamel 9 and their reported findings provide indisputable evidence of the quality, breadth and vigor of enamel research across the globe. Attendance at the Symposium by >30 early career researchers gives us confidence of the continuing vibrance and sustainability of the enamel field. In addition, it was particularly gratifying to see so many examples of interdisciplinary effort used to address increasing numbers of research questions in an integrated way, as was prioritized at Enamel VIII. Our first priority reflects the need to encourage and hasten the development of this valuable trend.

## Priority 1—stronger outcomes through interdisciplinary, integrative and translational approaches

Future advancements will be made at the interfaces of disciplinary boundaries (Nature News, 2015) and we encourage such collaborations as the preferred models for future working.

We propose positioning enamel within the context of the whole organism and not viewing this tissue in isolation. Enamel is unique but the cells that are central to its formation respond to equivalent signals and insults and share common pathways with cells throughout the body. For example, we need to improve our understanding of the regulatory/signaling pathways that contribute toward enamel development and how these align with, or differ from, those in other systems. These are important issues in framing our future research questions as we move toward translating findings from bench to chairside to population. We note the greatly increased participation by clinicians at Enamel 9 and recommend clinical involvement in framing translational research questions through identification of the clinical challenges and by contextualization of existing research. This is particularly important if we wish to remain responsive to advances and shifts in the direction of clinical treatment (e.g., minimally interventive dentistry, biomimetics) and open up new frontiers such as regenerative therapies and tissue engineering.

## Priority 2—better interfacing of biology with pathology

Research into the dysregulation of amelogenesis and biomineralization defects has significantly expanded over the past 5 years, adding vastly to classical understanding obtained from studies of normal tissue. Enamel VIII predicted that the extraordinary pace of technical advancement in genetics with concomitant decreases in costs for whole exome sequencing would see all genes underlying *amelogenesis imperfecta* (AI) identified by Enamel 9. We now know that this is far from reality, despite the fact that >18 genes have been identified where mutations underlie AI, with new variants—and new genes—being reported regularly (Smith et al., 2017) Each finding brings new pointers to direct our lines of enquiry in to the normal mechanisms of enamel formation, the basis of its highly complex architecture and the pathogenesis of disease. There are clearly many more AI-associated genes to be discovered but we face a significant lag in our understanding of pathological mechanisms and phenotyping of affected tissues.

Increased awareness of the high prevalence of Molar Hypomineralization (MH)^1^, (Hubbard et al., in press) highlights both a significant public health challenge and the need to better understand the pathology (and ultimately the prevention) of enamel opacities. It is likely that MH has multi-factorial causation, with environmental/lifestyle factors playing an important role but potentially with an underlying genetic component increasing individuals' susceptibility. Given suggestions that MH prevalence has increased in recent times, there may be parallels with other “lifestyle” related diseases that have seen greatly increased prevalence over relatively short timescales, such as cardiovascular disease and diabetes, where prevalence patterns reflect both patients' lifestyle and their genetic background. Individuals' susceptibility to enamel caries has already been linked to specific genetic polymorphisms (Bayram et al., 2015) and it seems likely that this paradigm could also account for variable susceptibility to fluorosis. Improved understanding of the relationship between genes and environmental factors in the patterns of normal and abnormal enamel development should be a major priority.

## Priority 3—better standardization of experimental variables

Attendees identified the urgent need for standardization of enamel phenotyping, both in AI and MH and also in animal models more generally. This includes the need to consider effects in different tooth types (molars as well as incisors) and in different tissues and compartments (including non-dental). The need to standardize phenotyping at each level, including detailed histological, ultrastructural, and molecular phenotyping in animal models and, wherever possible, of affected human teeth is paramount.

## Priority 4—better use of animal models

Animal models have added greatly to our understanding of the fundamental biology, chemistry and physical properties of enamel and will continue to do so but it is important that we understand the disadvantages and limitations of any models (and this applies equally *in vitro* and *in vivo*) when using them to better understand pathobiology and draw conclusions in comparisons with human teeth. We recommend that a standardized (and updatable) “check list” for phenotyping would greatly facilitate our combined understanding of the effects of gene mutations and knock-outs and enable cross-comparisons to be made with wild-type animals and “equivalent” human teeth. It would also mean that we could understand the limitations of our models and increase the rigor of our findings. For example, the recent reports of ER stress as a pathomechanism in AI and the view of AI as a proteopathy (Brookes et al., 2014) signals that we should be aware that confounded observations could arise from inadvertent stressing of the ameloblasts in *in vivo* models. Major differences in fluoride metabolism between rodents and humans (Angmar-Mansson and Whitford, 1984) is another important example.

## Priority 5—better collaboration for stronger collective voice and outputs

Compared with many others areas of scientific endeavor, enamel researchers are generally few and far between, making it difficult to be competitive at both topic and individual-performance levels. For example, it is likely that the “low hanging fruit” of gene discovery in AI has been picked by small groups in multiple countries but we know that for many AI patients, no causative gene has yet been identified. At Enamel 9, the proposal was made to establish an enamel genetics research network to support effective international collaboration by sharing findings in a community of trust (“GEnamel”: “Genetics for understanding Enamel”). We hope that GEnamel will provide a paradigm for further inter-group collaborations and data sharing. In turn, it was agreed that this network might usefully interface with a translational research and education network recently established to promote issues associated with developmental dental defects (The D3 Group)^2^. Together with the potential for more frequent Enamel Symposia (see below), such combined initiatives would move toward an identifiable international community with a strong collective voice without inhibiting the independent approaches to enamel research that often generate new growth points.

## Priority 6—harnessing new technologies

Furthering our understanding of pathology and the fundamental processes of normal enamel development can be accelerated by adopting new ways of looking at old problems. Recent advances in computational evolutionary genetics and molecular paleobiology bring new perspectives on the role of specific molecules in enamel formation (Qu et al., 2015). Additional materials science perspectives on enamel and its bio-inspired analogs would enormously benefit translational research seeking to develop new biomimetic materials and regenerative approaches to dental diseases. The need for greater inclusion of materials scientists in enamel research was recognized at Enamel VIII but this remains a strategic gap that has yet to be closed. Computer modeling of the complex data sets now being generated across multiple physiological compartments is also poorly represented in the enamel field. There is an urgent need to pro-actively recruit scientists from these disciplines to join us in both framing and answering questions.

New techniques in imaging and high resolution sample interrogation, such as focussed ion beam microscopy (Smith et al., 2016) and neutron reflectometry (Tarasevich et al., 2013) are yielding previously unobtainable insights, for example in elucidating the earliest stages of amelogenesis, the elusive “mineralization front” and the physico-chemical nature of the earliest enamel minerals. We predict that improved imaging techniques will help provide answers to outstanding questions in respect of our understanding of the mineralization front and the complex four dimensional movements of ameloblasts in dictating enamel structure. However, we still lack technical capability in tissue culture, notably three dimensional (3D) *in vitro* models and long term organoculture for investigating the relationship between ameloblasts, the enamel matrix and the other constituents of the enamel organ. We have long recognized that ameloblasts and the stratum intermedium form a functional syncytium (Skobe et al., 1995) and that studying ameloblasts in isolation seriously curtails both scope and significance of findings but a 3D culture system eludes us still and remains a much needed priority.

## Priority 7—increased attractiveness to funding bodies

Funding for enamel research remains a problem world-wide despite the fact that dental disease is a major public health burden. Affecting about 3 billion people, untreated caries in adults was the number one disease reported in the last Global Burden of Disease study (Murray et al., 2012). The international dearth of “programme grant” funding schemes that support multi-disciplinary teams and enable the integrated science approach we favor here (Priority 1) is a serious problem. We urge all colleagues to impress upon funders the advantages of such an approach. Increased attractiveness to funders should follow from improvements to our research quality, quantity, utility and visibility (Priorities 1–6). We acknowledge the continued support for the Enamel Symposia from NIH and the participation at Enamel 9 by Dr Jason Wan (NICDR), illustrating a working relationship that we should aspire to emulate with research funders across the globe. The classical funding envelope might also be expanded through translational initiatives involving industry, professional bodies and government. Enamel is the most highly mineralized mammalian tissue and its exquisitely ordered structure confers unique mechanical properties of significance to a broad audience, inside and outside of the health sciences. There is much we and others can learn from this tissue in the design of new materials and therapeutics. Opportunities therefore arise for generating research funding from our national research councils (e.g., for fundamental discovery, applied and translational research), industry (e.g., oral healthcare, orthobiologics, pharmaceutical) and charitable foundations (e.g., Wellcome Trust, Gates Foundation).

## Priority 8—increased and more effective communication

Given how rapidly recent findings have impacted our thinking plus the ongoing emergence of new technologies, our final recommendation is that due consideration be given to holding the Enamel Symposia more frequently and/or that additional short, sharply focussed meetings be held alongside larger events (e.g., the IADR global congress). We also need to ask how we might help researchers, especially those new to our field, access recommendations from previous Enamel Symposia and find key work published between meetings. These challenges might be met through an easily accessible website, increasing our currency and ensuring that past knowledge and thinking are not lost.

In conclusion, the Enamel Symposia uniquely bring together researchers from across the world, engendering a sense of community that fosters collaboration and exchange of ideas to accelerate research. We look forward to the next Symposium, Enamel 10, where progress against these identified priorities—and advances currently unforeseen—will be considered.

## Author contributions

JK drafted the paper; all co-authors added intellectual content. All authors agreed to the final manuscript.

### Conflict of interest statement

The authors declare that the research was conducted in the absence of any commercial or financial relationships that could be construed as a potential conflict of interest. The reviewer CR declared a shared affiliation, though no other collaboration, with the authors JK and SB to the handling Editor, who ensured that the process met the standards of a fair and objective review.

## Footnotes

^1^http://www.thed3group.org/prevalence.html

^2^http://www.thed3group.org
