# Supplementary material for: Enamel Research: Priorities and Future Directions
Source: Front Physiol. 2017 Jul 20;8:513. doi: 10.3389/fphys.2017.00513 (PMC5517532; doi:10.3389/fphys.2017.00513)
Supplement: Supplementary file 1 [file DataSheet1.PDF]

Ninth International Symposium on Dental Enamel: "Enamel 9"

Harrogate, UK: 30th October - 3rd November 2016

Abstracts, Q&As and Panel Discussions

Session 1: Enamel Formation: Stem Cells And Differentiation 2

Session 2: Cell Biology Of Amelogenesis 19

Session 3: Biomineralisation And Biomimetics 36

Session 4: Evolution And Development 58

Session 5: Enamel Pathology 75

Session 6: Amelogenesis Imperfecta 97

Session 7: Animal Models 122

Session 8: Enamel Mineral 144

Session 9: Caries 164

Session 10: Enamel Matrix Proteins 181

Session 1: Enamel Formation: Stem Cells And Differentiation

Moderator: Ophir Klein, University of California San Francisco, USA

#1 STEM CELL NICHE AND ENAMEL KNOT-LIKE STRUCTURE IN MOUSE INCISORS

NAKATOMI, C*, ISHIKAWA, Y, SAITO, K, NAKATOMI, M, JIMI, E and OHSHIMA, H

#2 BARX1 IS DOWN-REGULATED IN CHILDREN WITH NON-SYNDROMIC SUPERNUMERARY TEETH

MENTEŞ, A*, PEKEL, B, ÇEVİK, M, KOLDEMİR, M and SÜSLEYİCİ-DUMAN, B

#3 *MSX2* DEFICIENCY INDUCES THE DEDIFFERENTIATION OF THE OUTER ENAMEL EPITHELIUM

NAKATOMI, M*, IDA-YONEMOCHI, H, NAKATOMI, C, SAITO, K and OHSHIMA, H

#4 HYDROXYAPATITE TOXICITY FROM DENTAL GYPSUM WASTE ON MESENCHYMAL STEM CELLS

IZZUDDIN, AFA*, YUSTISIA, Y, ARDHIYANTO, HB, SUMONO, A and NAINI, A

#5 VITAMIN K2 ENHANCES VITAMIN D POSITIVE EFFECT ON DIFFERENTIATION OF OSTEOBLASTS

RIKSEN, EA*, LIAN, A, MØLLER, M and RESELAND, JE

#6 AMELOBLASTIN PEPTIDES PROMOTE GROWTH AND DIFFERENTIATION OF MESENCHYMAL STEM CELLS

STAKKESTAD, Ø*, LYNGSTADAAS, SP, VONDRASEK, J, GORDELADZE, JO and RESELAND, JE

#7 HISTONE MODIFICATION DURING AMELOBLAST DIFFERENTIATION**

LI, F, WAN, M, XU, X, ZHOU, X and ZHENG, L*

***Presented by Pamela Den Besten*

KEYNOTE ADDRESS: LONG IN THE TOOTH: THE WHO, WHAT, WHEN, WHERE AND WHY OF AMELOBLAST STEM CELLS

OPHIR KLEIN M.D., Ph.D.

Hillblom Distinguished Professor in Craniofacial Anomalies; Epstein Professor of Human Genetics; Professor of Orofacial Sciences and Pediatrics; Chief, Division of Genetics; Chair, Division of Craniofacial Anomalies; Medical Director, Craniofacial Center; Director, Program in Craniofacial Biology; University of California, San Francisco

Email: Ophir.Klein@ucsf.edu

The continuously growing rodent incisor provides a model that allows us to understand how adult stem cells can produce ameloblasts throughout an animal's life. This system allows for powerful integration of investigations into how stem cells function, how they evolved, and how their behaviors are coordinated across tissues. I will present data from our recent work focusing on development and renewal of the rodent incisor. This organ, like many others such as the skin, gastrointestinal tract, and hematopoietic system, is dependent on the continuous generation of progeny from stem cells that have the capacity to self-renew as well as to give rise to the required differentiated cell types. I will first discuss candidate approaches to the identity and location of the stem cells, as well as unbiased screening techniques that can be used to deconstruct the system. I will then discuss the transcriptional and signaling networks that regulate them, focusing in particular on our new work examining the Hippo pathway. Finally, I will introduce evolutionary perspectives on continuously growing teeth.

DISCUSSION:

Jim Simmer (Michigan): Teeth tend to erupt at the rate at which they wear, and when teeth break off they wear much faster. When doing these different conditional knockouts, if you're affecting the wear rate you'd expect the kinetics to be affected.

Ophir Klein (San Francisco): That's a great question. As I mentioned, these investigations are preliminary, and the only experiments that we've done so far in terms of pathway manipulations are with a Notch-blocking antibody. We are excited to look at knockouts, and one family of genes that we've worked on a lot are Sprouty genes, which when knocked out, lead to tusk-like phenotypes. We would predict that there are big changes in the proliferative behaviour of the stem cells, and now we are going to look at that. There are 4 or 5 other knockouts where you can see ectopic enamel on the lingual side and a number of other knockouts where there is hypoplastic enamel or other kinds of hypoactive behaviour. One of the postdocs in the lab is now going back and doing the experiments that we should have done many years ago, in terms of describing the basic behaviour of the system. I should also mention that we, like many other groups, have been sectioning in 2D to try to get a perfect section down the middle of the cervical loop. But when doing this, you miss a huge amount of biology because of the 3D structure. What we've been developing are methods to dissect off the entire proximal part of the cervical loop and imaging in 3D.

#1 STEM CELL NICHE AND ENAMEL KNOT-LIKE STRUCTURE IN MOUSE INCISORS

NAKATOMI, C[1*], ISHIKAWA, Y[2], SAITO, K[3], NAKATOMI, M[4], JIMI, E[1] and OHSHIMA, H[3]

[1]Division of Molecular Signaling and Biochemistry, Department of Health Promotion, Kyushu Dental University, Kitakyushu, Japan; [2]Department of Oral Health Sciences, Faculty of Nursing and Welfare, Kyushu University of Nursing and Social Welfare, Tamana, Japan; [3]Division of Anatomy and Cell Biology of the Hard Tissue, Department of Tissue Regeneration and Reconstruction, Niigata University Graduate School of Medical and Dental Sciences, Niigata, Japan; [4]Division of Anatomy, Department of Health Promotion, Kyushu Dental University, Kitakyushu, Japan

Email: r15nakatomi@fa.kyu-dent.ac.jp

OBJECTIVES: Rodent incisors are continuously growing and maintained by both cell proliferation at the apical end and the attrition of the incisal edge. Recent molecular biology studies have demonstrated the existence of a niche for self-renewing adult stem cells in this apical end referred to as “labial cervical loop” or “apical bud.” Recently, we have reported that the enamel knot (EK)-like structure is eternally maintained in the apical bud of postnatal mouse incisors (Arch Oral Biol 60: 1122-1130, 2015). However, the relation between this EK-like structure and the adult stem cells remains to be elucidated. This study aimed to clarify the spatial relationship between the EK-like structure and the localization of label-retaining cells (LRCs), putative quiescent stem cells.

METHODS: The birthed animals at postnatal Day 3 or Week 3 were analyzed following the prenatal BrdU labeling (Cell Tissue Res 348:95-107, 2012) or the prenatal doxycycline injection into the TetOP-H2B-GFP mice and without labeling. The cryo- or paraffin sections were processed for immunohistochemistry for BrdU, GFP, and Gli1 and *in situ* hybridization for EK marker genes such as *Shh*, *Bmp4*, *Fgf4*, *Msx2*, and *P21* and Shh signaling-related genes such as *Gli1* and Patched *(Ptch)1*. Furthermore, to investigate the whole expression patterns of the examined marker genes or proteins, serial frontal or sagittal sections of the apical bud were reconstructed into 3D images.

RESULTS: The molar tooth germ-like structure is maintained indefinitely in the apical bud of incisors. This structure includes the expression of all the examined markers for the EK, and dense LRCs, Gli1 (+)-cells, and Ptch1 (+)-cells were co-localized in the outer enamel epithelium of the apical bud.

CONCLUSION: The tooth germ is eternally maintained and that the quiescent dental stem cells are regulated by Shh signaling in the apical bud.

DISCUSSION:

Ophir Klein (San Francisco): Have you looked in 3D at the structure to see how similar it is to an embryonic enamel knot? How similar are they structurally?

Chihiro Nakatomi (Kyushu): Do you mean incisor? We haven’t looked at this yet.

Ophir Klein (San Francisco): It could be interesting to see if they are molecularly similar.

#2 BARX1 IS DOWN-REGULATED IN CHILDREN WITH NON-SYNDROMIC SUPERNUMERARY TEETH

MENTEŞ, A[1]*, PEKEL, B[1], ÇEVİK, M[2], KOLDEMİR, M[2] and SÜSLEYİCİ-DUMAN, B[2]

[1]Department of Pediatric Dentistry, Marmara University, Istanbul, Turkey; [2]Department of Molecular Biology, Marmara University, Istanbul, Turkey

Email: alimentes@gmail.com

OBJECTIVES: Apart from the syndromic conditions listed as many as 26 in OMIM, the etiology of the non-syndromic or non-disease related supernumerary teeth (NST) remains to be a mystery. The aim of this study was to investigate different gene expression patterns of the gingival tissue in child patients with NST using RNA microarray assays.

METHODS: Sixteen patients; 9 boys, 7 girls (aged between 8-11) diagnosed with NST in premaxillary region were selected from children admitted to Department of Paediatric Dentistry, Marmara University. These patients had a total of 23 NST including 8 conical, 7 tuberculate and 8 supplemental NST types. Sixteen healthy children of similar age and gender were recruited as controls. This study was approved by the ethical committee of Medical Faculty, MU. Gingival tissues from the extraction sites were collected during NST surgeries and immediately immersed into RNALater® (ThermoFisher Scientific). After tissue disruption and homogenization, RNA isolations were completed using RNeasy® Microarray Tissue (Qiagen). 100ng Total RNA with RNA integrity number (RIN) determined with Agilent Bioanalyzer kits and samples with RIN>7 were included in the study. One-color microarray-based gene expression analysis was completed using Agilent Human Gene Expression Array V2 design. Preprocessing and Statistical Analysis were done using GeneSpring software (Agilent). Probe Intensities were quantile normalized and significant probes were selected according to moderated t-test with Benjamini-Hochberg correction.

RESULTS: In the microarray analyses, 112 genes showed at least two-fold change in the NST patients. Comparison analyses identified 103 down-regulated and 9 up-regulated genes. Among all NST types, *BARX1* was the single common downregulated and *CCL13* the single common upregulated gene. *BARX1* was more downregulated in boys, whereas *MMP7* was more upregulated in girls.

CONCLUSION: Investigating the expression profiles of the NST patients seems to give valuable information about the mechanisms of the dental differentiation, growth and regeneration.

DISCUSSION

Janet Moradian-Oldak (Southern California): Do you know if there are animal models of BARX1 and what can we learn from them?

Ali Menteş (Marmara): I think the next step with BARX1 is working with animal models; other genes may be important but we don't know yet. It is not related to any other genes shown in the literature. *BARX1* is the only gene that has been found.

#3 *MSX2* DEFICIENCY INDUCES THE DEDIFFERENTIATION OF THE OUTER ENAMEL EPITHELIUM

NAKATOMI, M[1 2]*, IDA-YONEMOCHI, H[1], NAKATOMI, C[3 4], SAITO, K[1] and OHSHIMA, H[1]

[1]Division of Anatomy and Cell Biology of the Hard Tissue, Department of Tissue Regeneration and Reconstruction, Niigata University Graduate School of Medical and Dental Sciences, Niigata 951-8514, Japan; [2]Division of Anatomy, Department of Health Promotion, Kyushu Dental University, Kitakyushu 803-8580, Japan; [3]General Dentistry and Clinical Education Unit, Niigata University Medical and Dental Hospital, Niigata 951-8520, Japan; [4]Division of Molecular Signaling and Biochemistry, Department of Health Promotion, Kyushu Dental University, Kitakyushu 803-8580, Japan

Email: nktm-emb@umin.ac.jp

OBJECTIVES: It has been stated that ameloblasts are lost and abnormal enamel is formed during tooth development in *Msx2* null mutant mice. However its precise mechanism is still to be elucidated. In this study, we analyzed tooth development of *Msx2* null mice in detail to clarify how *Msx2* is required for normal enamel formation.

METHODS: *Msx2* null and control mice were sacrificed at postnatal day 3, 5 and 9 and week 10, 20 and 25. Hematoxylin and eosin staining, *in situ* hybridization, immunohistochemistry, reverse transcriptase-polymerase chain reaction, transmission electron microscopy, electron probe micro-analysis, micro-computed tomography and organ culture analyses were carried out.

RESULTS: *Msx2* expression was detected in ameloblasts, stratum intermedium (SI) cells and the outer enamel epithelium (OEE) in wild-type mice. Ameloblasts were normally polarized in the early differentiating stage and expressed some marker genes such as ameloblastin, amelogenin and enamelin in *Msx2* mutants. In addition, SI cells were also differentiated and expressed Notch1 and Sox2 normally in the mutants, suggesting that *Msx2* deficiency does not affect early differentiation of ameloblasts and SI cells. By contrast, the OEE, which is usually composed of a thin single cell layer, was transformed into keratinized stratified squamous epithelium and keratin was ectopically accumulated in the enamel organ of *Msx2* mutants, leading to cyst formation in the enamel organ, depolarization of ameloblasts and subsequent abnormal enamel deposition. Remarkably, some hair follicle specific keratins were also ectopically detected in the mutant enamel organ, indicating that the OEE has potential to express hair keratins but it is usually inhibited by *Msx2.*

CONCLUSION: *Msx2* is required to maintain the outer enamel epithelium as a thin single cell layer, which is essential for normal ameloblast function and enamel formation.

DISCUSSION

Ariane Berdal (Paris): Have you evidence that the enamel peptides could be overexpressed in your system? Would you suggest that this peptide would influence your process?

Chihiro Nakatomi (Kyushu): I haven't compared the intensity of the expression but I think there are probably the same levels between wild types and mutants. A comprehensive study has not been undertaken yet.

Ophir Klein (San Francisco): What are the transcriptional targets of MSX2? Also, I've heard that if you pick out cells from the OEE (outer enamel epithelium), even distal from the cervical loop, that they have stem cell-like properties in culture. Do you think this is a fate-shift of these cells to become more like another type of keratinocyte?

Chihiro Nakatomi (Kyushu): I think they turn into a keratinocyte. I'm very interested in target genes of MSX2 transcription factors and will do microanalysis to compare the target genes during OEE de-differentiation.

Alex Vieira (Pittsburgh): In humans, mutation of *MSX2* causes a craniosynostosis syndrome which has a hyperproliferative activity in the area of the sutures. Do your mice have other bone abnormalities or is this localized?

Chihiro Nakatomi (Kyushu): I think the mice have cranial abnormalities and craniosynostosis happens.

#4 HYDROXYAPATITE TOXICITY FROM DENTAL GYPSUM WASTE ON MESENCHYMAL STEM CELLS

IZZUDDIN, AFA*[1], YUSTISIA, Y[2], ARDHIYANTO, HB[3], SUMONO, A[3] and NAINI, A[4]

[1]School of Dentistry, University of Jember, Indonesia; [2]Oral Biology Dept., University of Jember, Indonesia; [3]Biomedical Dept., University of Jember, Indonesia; [4]Prosthodontic Dept., University of Jember, Indonesia

Email: ahmadfaris16@gmail.com

OBJECTIVES: Hydroxyapatite [$Ca_{10}(PO_4)_6(OH)_2$] can be synthesized from dental gypsum waste type II, III, and IV [$CaSO_4.2H_2O$] by hydrothermal method. As a potential biomimetic material, synthesized hydroxyapatite from dental gypsum waste (DG-HA) must have ideal biocompatibility which can be determined through toxicity test. The objective of research is to evaluate DG-HA toxicity type II, III, and IV on Rat's Bone Marrow Mesenchymal Stem Cells (RBM-MSCs).

METHODS: DG-HA type II, III, and IV were immersed in culture medium with concentrations of 10 µg/ml, 100 µg/ml, and 1000 µg/ml. The immersions were then added into 96 well plate containing $5x10^3$ RBM-MSCs /well. After 24 hours of incubation, cell death were observed using MTT assay.

RESULTS: Lowest average of RBM-MSCs death in concentrations 10 µg/ml (28.978 ± 1.425%) and 100 µg/ml (29.320 ± 0.468%) showed by DG-HA type III. Lowest average of RBM-MSCs death in concentration 1000 µg/ml (33.377 ± 1.605%) showed by DG-HA type II. Highest average of RBM-MSCs death showed by DG-HA type IV in all concentration: 10 µg/ml (33.204 ± 2.518%), 100 µg/ml (34.690 ± 0.716%) and 1000 µg/ml (37.798 ± 10.993). The death of RBM-MSCs was probably caused by hydroxyl (OH^-) ions that formed during synthesis process. The differences of RBM-MSCs death in between groups might be affected by different compiler and additive subtances in dental gypsums as the source of synthesized hydroxyapatites. DG-HA type IV was assumed to contain cuprum (Cu) -a compiler as a coloring agent that was insoluble during synthesis process characterized by pink colored of hydroxyapatite particle. It probably induces RBM-MSCs apoptosis. DG-HA type IV also was assumed to contain calcium chloride ($CaCl_2$) and Magnesium Chloride ($MgCl_2$)- as additive substances to form fine gypsum particle that was insoluble during synthesis process. They probably induced RBM-MSCs necrosis. DG-HA type II and III was expected to have less coloring agent and additive substances.

CONCLUSION: Toxicity of DG-HA type II, III, and IV were lower than 50% in all concentrations on RBM-MSCs.

Abstract presented in session 2.

No questions.

#5 VITAMIN K2 ENHANCES VITAMIN D POSITIVE EFFECT ON DIFFERENTIATION OF OSTEOBLASTS

RIKSEN, EA[1]*, LIAN, A[1], MØLLER, M[2] and RESELAND, JE[1]

[1]Department of Biomaterials, Institute for Clinical Dentistry, University of Oslo, Norway; [2]Axial Vita AS, Oslo, Norway

E-mail: elisaba@odont.uio.no

OBJECTIVES: Alveolar bone loss is common after destructive marginal periodontitis. Dietary vitamin supplements might be beneficial in bone healing and mineralization. Vitamin D stimulating effect on bone homeostasis is well known, and there is a clear link between vitamin K nutrition and skeletal integrity, including fracture reduction. The aim was to investigate effects of two synthetic forms of vitamin K2 (MK4, MK7) and vitamin D (vitD) alone and in combinations on differentiation of primary human osteoblasts *in vitro*.

METHODS: Primary human osteoblasts (NHO-2) were cultivated for 1, 3, 7, 14 and 20 days with MK4, MK7 (1µM, 10µM), or with vitD (10^{-8}M) alone, or in combinations. Bone markers (IL-1b, IL-6, OPG, OC, Leptin, OPN, PTH, TNF-a, ACTH, Adiponectin and Insulin) were quantified using Luminex and presented as a % of untreated control cells.

RESULTS: MK7 and MK-4 gave an acute increase in OC secretion. In combination with vitD, 10µM MK4 had the highest effect (250%). Osteopontin (OPN) was enhanced by both MK4 alone or in combination with vitD (175%, 3 days), whereas MK7 had no effect. Parathyroid hormone (PTH) was initially increased after 1 and 3 days with all MK-4 treatments up to 163%. In contrast, MK7 initially reduced PTH to 54% but enhanced secretion after 3 days 151%. Secretion of osteoprotegrin (OPG) was reduced by MK7 both alone (50%) and in combination with vitD (25%). MK4 or vitD alone or in combination had no effect on OPG secretion. MK4 had a dose-dependent effect on IL-6 secretion. In combination with vitD, MK4 reduced the secretion to 55 % of control. MK7 or vitD had no effect on IL-6, whereas the combination of MK7 and vitD reduced the IL-6 secretion to 65% of the level of untreated cells.

CONCLUSION: In conclusion vitamin K2 in all test solutions increases osteoblast secretion of bone matrix proteins OC and OPN; but the two isoforms of vitamin K2 had different effect. MK7 is a larger molecule compared to MK4 that readily dissolves in the cell media. MK4 had more potent effect on OC, OPN, PTH, OPG and IL-6. However he combination of vitamin K2 (MK4 and MK7) with vitamin D and had most stimulative effect, thus indicating that vitamin K2 potentiate the osteopromotive effect of vitamin D.

DISCUSSION

Sylvie Babajko (Paris): Does the vitamin K interfere with the vitamin D signalling pathway?

Elisabeth Riksen (Oslo): We think the effect is combined. Vitamin K is a co-enzyme, so together we have a good effect.

#6 AMELOBLASTIN PEPTIDES PROMOTE GROWTH AND DIFFERENTIATION OF MESENCHYMAL STEM CELLS

STAKKESTAD, Ø[1]*, LYNGSTADAAS, SP[1], VONDRASEK, J[2], GORDELADZE, JO[1] and RESELAND, JE[1]
[1] Institute of Clinical Dentistry, Department of biomaterials, University of Oslo, Oslo, Norway; [2] Institute of Organic Chemistry and Biochemistry, Czech Academy of Sciences, Prague, Czech Republic

Email: oystein.stakkestad@odont.uio.no

OBJECTIVE: Extracellular ameloblastin (AMBN) is processed into fragments, whereof 17 kDa (N-terminal) and 23 kDa (C-terminal) products are found during amelogenesis. AMBN is also expressed by human mesenchymal stem cells (hMSC) and may act in modulation of tissue regeneration. However the effect of the various processed products on hMSC remains unchartered. We have thus investigated the effect of proteins (N-term and C-term) and peptides that mimic the processed products from AMBN, on hMSC to see if and how these molecules affect these cells.

METHODS: hMCS was incubated with (0.1 μM and 0.2 μM) AMBN-WT, N-term, C-term, Del exon 5 (WT without Exon 5), or peptides (0.2 μM) spanning region exon 2-6 (aa27-113) for up to 28 days. Effect on proliferation was measured by [^{3}H]-thymidine incorporation. Gene expression was measured by RT-PCR. Mineralized nodules from cells differentiated with osteogenic medium with and without AMBN or its fragments were stained with Alizarin Red. The levels of cytokines/chemokines in cell culture medium from each time point were quantified using Luminex.

RESULTS: AMBN-WT enhanced the proliferation and secretion of the chemokines RANTES 1953 ± 782 (P=0.01), IP-10 2236± 1357 (P=0.029) and MIP-1a 1215 ± 231 (P=0.001) (% of control). The N-term fragment enhanced the secretion of RANTES 451 ± 57 (P=0.0004), IP-10 314 ± 77 (P=0.003), IL-8 244 ± 133 (P=0.038) (%), and mRNA of RUNX2 2.7-fold ± 1.2 (P=0.015). The exon-5 peptides increased mRNA of osteocalcin 2.5-fold ± 1.5 (P=0.009), and the formation of mineralized nodules to 167 ± 24(%) (P=0.029), while the peptide corresponding to aa81-98 of Exon 5 induced proliferation 4.5-fold (P=<0.0001). The C-term did not have effects on any of the parameters investigated.

CONCLUSIONS: The findings suggest a role for the N-terminal part of AMBN, and its processed products, in proliferation and differentiation of hMSC into a mineralizing cell type.

DISCUSSION:

Ariane Berdal (Paris): Do you have any idea of the *in vivo* pattern of the different forms because you suggested that this would vary during tooth development? You mentioned that during late tooth development they would have a privileged distribution, and that could control the bone.

Øystein Stakkestad (Oslo): In late stage development, this peptide is translated into the full length ameloblastin.

Ophir Klein (San Francisco): How much is known about how this signal is transduced and what is downstream of it?

Øystein Stakkestad (Oslo): Not sure. We don’t know yet which receptors ameloblastin binds to.

#7 HISTONE MODIFICATION DURING AMELOBLAST DIFFERENTIATION**

LI, F[1 2], WAN, M[1 2], XU, X[1 2], ZHOU, X[1 2] and ZHENG, L[1 2 3]*

[1]State Key Laboratory of Oral Diseases, Sichuan University, Chengdu, China, 610041; [2]West China School of Stomatology, Sichuan University, Chengdu, China, 610041; [3]Department of Pediatric Dentistry, West China Hospital of Stomatology, Sichuan University, Chengdu, China, 610041

***Presented by Pamela Den Besten, University of California at San Francisco*

Email: liwei.zheng@scu.edu.cn

OBJECTIVES: Enamel Formation is precisely regulated by mechanisms in multiple layers both genetically and epigenetically. Histone modifications including H3K4me3 and H3K27me3 has been proposed to be actively involved in cell fate commitment. Balance between H3K4me3 (transcription activation related) and H3K27me3 (transcription repression related) produces diverse chromatin architectures and results in "poised", "activated" or "repressed" status of differentiation-related genes. The cervical loop region of mouse incisor is believed to be an epithelial stem cell niche, and thus an ideal model to study stem/progenitor cell differentiation for its continuous growth throughout the lifetime.

METHODS: New-born mouse incisors were dissected, and immunofluorescence was carried out to identify H3K4me3 and H3K27me3 status in cervical loop region and ameloblast-lineage cells along the epithelium sheet. Different stages of ameloblast-lineage cells were isolated under microscope followed by RNA purification/protein extraction. Histone methylases and demethylases were quantified by qPCR and western blotting. Sonic hedgehog (SHH, an early essential cytokine for ameloblast differentiation that mainly expressed in proliferating and differentiating ameloblasts) expression level was tested in ameloblast differentiations by qPCR and western blotting. Furthermore, ChIP assay was applied to test the histone modification status of *Shh* and the affinity between the enzymes and promoter region of *Shh*.

RESULTS: Histone modification changed during ameloblast-lineage cells differentiation. During this process, a stage-specific expression manner of H3K4me3/H3K27me3 methylases and demethylases was detected. The promoter region of *Shh* went through a switch of H3K4me3/H3K27me3 during ameloblast differentiation. Further investigation indicated that this switch was induced via H3K4me3/H3K27me3 specific methylases and demethylases through binding with the *Shh* promoter region.

CONCLUSION: These results indicated an epigenetic switch of histone modification during ameloblast differentiation, which is essential to enamel formation.

DISCUSSION

Tom Diekwisch (Dallas): I thought it was a wonderful study. It would be interesting to look at a second repressive marker. Also, the global change in histone markers was quite impressive. Is sonic

(hedgehog) the only target gene affected or are a number affected? Thinking about MSX, a global analysis would be interesting.

PANEL DISCUSSION:

Bernhard Ganss (Toronto): A question for all of you, but mostly for Ophir, is whether the permanently erupting incisor is just a model where we can study all of these phenomena that happen in non-continuously erupting teeth but are then terminated for some reason, or is it more than a wonderful model system?

Ophir Klein (San Francisco): I think it's important to not over promise about whether these kinds of epithelial stem cells, which is, I think, what you are probably getting at, are going to be directly applicable as the next step to making teeth for people. Clearly there are big evolutionary differences between animals that have continuously growing teeth and those that don't, so the way I look at it is that first of all I think it's a really amazing system for the study of adult stem cells generally, not just specifically for the dental community, just like how we've learned a ton about mammalian stem cell biology from studying the hair follicle or other organs that you might not think of as immediately biomedically critical. I think another thing is that, related to the point that I made at the end of my talk about this being something that's evolved so many times independently, this suggests that there's some kind of switch that can be flipped relatively easily. It's happened at least eight or so different times in mammalian evolution, so there probably is a lot that we can learn, although we don't have those exact stem cells in human epithelium, about what our epithelium does. The last point to make is that although the focus here is more on epithelium and ameloblasts, incisor mesenchyme is a very important system for understanding how you can really rev up a massive regenerative capacity, so the way I look at it is, in any kind of biological problem, you want to really, first of all understand the basics and where exactly that is going to take us, we never really know. I think there are going to be some translational implications of this but it's also important for us to not act like we are going to immediately go from understanding these stem cells to being able to repair human teeth; I think translation is a process and this is one step in the process.

Pam Den Besten (San Francisco): I think people who have been involved in the enamel field for a while now know there's this continual debate about molar versus incisor. What is the best model, what are we looking at and why are we looking at it? And I think it's really interesting to look at the tooth as more of a model system for understanding pathways and the incisors are a really interesting model to understand that. I think as Ophir said it's going to take us some time to figure out exactly where it's going to take us but we first have to understand it.

Ali Menteş (Marmara): I'm a dentist so it's very good to see all those rat models together but I think a supernumerary teeth model is also quite interesting to work with. We can look first in humans but then maybe you can extrapolate to the rats afterwards, so that's why I'm trying to get what we can get from those supernumerary teeth patients, and if someone is interested I can send my specimens to anybody and you can work together with the rat models. Probably the thing in humans and in rats is something different, as you know the roots are not growing in humans so it must be something different.

Øystein Stakkestad (Oslo): I haven't actually done any research on tooth but the differences in proliferation and differentiation of the spliced products of ameloblastin could be important for maybe a continuous proliferation of stem cells and their control.

Mitsushiro Nakatomi (UMIN): In humans, teeth are not continuously growing but I think a similar mechanism can be applied for human teeth development. The rodent incisors can grow continuously but during mouse molar development, or all human teeth development, there must be some local stem cells to make it grow. For example, in my mouse model, *Msx* knockout mice, the same phenotype happens in both the incisors and the molars so during molar development the keratin is accumulated by the enamel organ and no enamel is deposited so I think during molar development in mice some local stem cells exist to make it grow towards the root elongation and also molar epithelial growth. So, to understand the stem cell mechanism is also important to understand human tooth development.

Pam Den Besten (San Francisco): I would like to encourage people who are making these wonderful models and looking at the incisors to actually also look at the molars, because I think we could learn a lot. We all know that there are things that show up in the incisors and not the molars, we've got the model, so it would be interesting to look at both teeth.

Olivier Durverger (NIH): I have a question for Dr Menteş about the BARX1 study. Have you looked in those patients to see if they have SNPs in the promoter region or on the locus of the gene? The second question is: have you also looked at gene profiling in the dental pulp because tooth development starts with signals from both the mesenchyme and the epithelium so I think you may find very interesting information from the pulp as well.

Ali Menteş (Marmara): We haven't looked at this, because the pulps of the supernumerary teeth are so small, we cannot get any good specimens. Our first aim was to collect those tissues but we couldn't, so we took the gingival part around those supernumeraries. In Finland there was a congress (Tooth Morphogenesis and Differentiation), where I also presented DNA results of the supernumerary teeth and I looked for the copy number variations of those children, but every child has a different DNA profile so we couldn't combine together all of the results. So if you look at the DNA especially- what we collected from saliva- we couldn't find any relation with those RNA.

Derk Joester (Northwestern): I have a question for Ophir, a technical one and a general one. The technical one is that I didn't quite understand why you needed 84 microarrays for the gene network analysis. Can you comment on the sample number? The second question is that I thought the model system was amazing. When you did the analysis, did you already compare the lingual with the labial loop, as there should be a lot of interesting things in that comparison?

Ophir Klein (San Francisco): Those are both good questions. In terms of the first one, I didn't really understand that either initially. We did this in collaboration with Mike Oldham at UCSF who uses this approach in the brain, and he kept saying you need to use a lot of samples, like 100 samples, and

I didn't understand why at first. If you think about it, what's driving the variation that enables you to track whether these genes are going up or down together, it is that you're essentially micro-dissecting out a very similar region. Although Kerstin, the postdoc who did this, is a very precise and careful person and so the samples should be as close to identical as possible, the micro-dissection generates a certain amount of heterogeneity which is just based on the way that you are cutting it. So what that means is that you have slightly different percentages of cell types in each sample. But you also need them to be very, very similar, and so the only way you can have these be similar enough and yet have these differences, is by having a lot of them. So when I said, can we try 50? He said, no, 50 is not enough, you've got to do 100. I was amazed that when you feed this data into the co-expression algorithm, you get the entire structure of the system, so that kind of segues into this next question of can you look at the lingual versus the labial cervical loop. What Ariane was saying was that what we call the lingual cervical loop is a very thin piece of epithelium that connects around with the labial side. Being able to micro-dissect that out would be technically very difficult, so I don't know of a lot of people who are trying to do that. One thing that has occurred to me in retrospect that would have been a smarter thing to do, so maybe now we can go back and do it, is that the level of resolution that you have with this approach scales with the size of the tissue. So this has been very useful for us to look at the region that we micro-dissected, which is the proximal component containing the epithelium and mesenchyme, but there is a level of resolution higher that will look specifically within the labial cervical loop, and we currently have too large a piece of tissue for that. Actually, the epithelial compartments that we have in terms of the cervical loops are very similar among all the tissues, because that's not where we're cutting, and so now what we'd like to do is go back and do a second round of this so we can micro-dissect off that epithelium, like I showed you with the Hippo project, to look at that apical bud region.

Colin Robinson (Leeds): With regards to the rat incisor, if I remember correctly, eruption slows down as the animal ages, so is it possible therefore that this is not continuously erupting, it's just erupting over a very long time and the animal dies before it's finished?

Ophir Klein (San Francisco): I have heard from people that they have found old squirrels and other types of rodents where the teeth have stopped growing. It is true that there are changes over time, I didn't show this for the sake of time but we've been looking at animals immediately post-natal, one week, a month and three months of age and you see things changing over time. I think it could be a really interesting system for the study of stem cell ageing; the problem is getting somebody to do that project when you have to age all these mice and presumably age mutants, and this is really painful.

Jukka Jernvall (Helsinki): So the slowing down could be a reasonable model, because in molars of rodents like voles it can be very old individuals that will eventually develop roots in those species that do have the roots, and we even have fossils where you have roots in the 3rd molar, but the 1st and 2nd molar are basically continuously growing, so in this kind of model it's the slowing down process and it differs between species.

Ophir Klein (San Francisco): It could be really cool to do cross-species comparisons. I was thinking about this when Ben (Bernhard Ganss) asked the question about how good a model is the incisor, and

that is related to the question of, "*are these teeth continuously growing or are they just growing for a really, really long time*". That, I think, brings up a really interesting stem cell biology question that we're trying to think about how to ask, but we haven't figured out a good way which is, you can imagine that there are two different ways that the system could be set up. One way is what you're saying, which is that this is a continuation of a process that you see in the molars, where they're growing for a period of time, they might have cells that look like these stem cells, and that's basically shut off and converts to becoming roots in molars. So there could be a process by which the cells in the embryo are locked away and persist into the adult. The other possibility, which we have a little evidence for, but it's not very strong, is that there are two separate processes. First, you have the normal developmental process, and then you have this continuously growing module that is tacked onto it. That could be a separate molecular cascade. I think that's a very interesting question of which of those two is the case and that would kind of go to this question that Ben was asking about how good a model is this.

Ariane Berdal (Paris): What is striking when you present all of your data is that the same transcription factor may be involved in a developmental cascade, and the same transcription factor may be active on enamel forming genes, which means that one factor would be acting as a developmental process that can be stem cells and so on but you have also the possibility of the level of activity being at the ameloblast, which is a second level, a cell-autonomous level. Thirdly, we have investigated the impact of osteoclast activation during tooth development and osteoclast activation recruits and displays a growth factor from bone to the adjoining tooth. If you modify the osteoclast activity you accelerate the process, which means you have several levels to consider. So probably in MSX2, the osteopetroses or so-called inhibition of osteoclast activity would be part of playing a role on the tooth phenotype, so I think we will get some light on the levels of regulation which are quite complex.

Tom Diekwisch (Dallas): I thought I'd comment on Colin's comment and question, also in response to Ophir and Jukka. Actually, molars also continuously grow and erupt throughout life. There was a huge debate during the beginning of the last century and one of the most prominent examples of the publication history was actually the archaeological ageing of humans because your teeth continue to erupt, your skulls become taller and archaeologists have used this measurement extensively to determine the age of skeletal populations. The older you get, the taller your skull becomes, not much but statistically significant, so even molars continue to grow, not that extensively, but they do.

Jim Simmer (Michigan): You're labelling these cells at the beginning near the proximal end and I was wondering how long you followed them, because, like you said, the tooth is getting larger and larger, so that means that some stem cells have to be moving further forward from a band of cells to keep supplying this ever-growing surface of ameloblasts as the tooth grows further up. So even as you lay down more enamel, the amount of ameloblasts covering it has to be increasing, and I'm wondering how long you followed these cells as they are moving forward to see how the ameloblasts are able to continuously add cells further incisally to cover the ameloblast layer as the tooth grows bigger.

Ophir Klein (San Francisco): I didn't show that data, but we kind of stop when we get into the pre-ameloblast region, so we're not moving very far distally for both technical reasons and because our focus has been more on the progenitor cells, but as part of this analysis of the kinetics of the system we've been trying to get calculations about the number of cells produced and it's pretty staggering, it's a huge number of cells. It's not trivial to make those calculations but it's tens of thousands of cells per day, and that's why it's really important to look in three dimensions and to try to develop techniques that enable that because when you just take the slice down the middle you lose a lot of the expansion that happens. We're in the process of making those calculations and I'm happy to show you afterwards what the numbers look like.

Bernhard Ganss (Toronto): We're looking at these ameloblasts differentiating along and all the textbooks tell us that you have to have a pre-dentine matrix for the ameloblasts to form first. So are we starting to look at mechano-biology aspects of a stiffer matrix that these cells need in order to do what they're supposed to do?

Ophir Klein (San Francisco): The stuff I talked about with the YAP signalling, the non-canonical regulation that you can see through integrins and FAK and CDC42, part of what that pathway does is mechano-sense. There's a couple of different ways you can look at it. One way, which we think would be more exciting, although I don't have a lot of evidence for it, is it's possible that there's actually a component of this which is a long-term transmittal of force from the gnawing end of the incisors, such that, for example, when you're trimming something, that is sensed and this force somehow activates the stem cells. Another possibility that is perhaps less interesting, but maybe more of the case, is that you're not really transmitting these long range forces, but it's more very local effects of the basement membrane that are sensed. We've done some work *in vivo* and in cell culture to try and get at this mechano-sensing, and we're trying to do a cell culture CRISPR screen to find factors that would be involved. It doesn't really make sense to us yet why, in essentially the same mechano-sensing environment, you have these two populations of the transient amplifying cells with incredibly high levels of nuclear YAP adjacent to the stem cells that have very low levels of nuclear YAP, so something in addition to mechano-sensing must be at play, because the basement membrane around those two cell types is similar.

Ariane Berdal (Paris): We have published some months ago that SOST (sclerostin), which is a biomechanical response of osteocytes to regulate bone metabolism, is expressed in the odontoblast depending on the eruption stage. So probably the inhibition of the Wnt pathway might contribute to the regional activity of YAP biomechanics but the coupling is different from bone.

Session 2: Cell Biology Of Amelogenesis

Moderator: Ariane Berdal, Centre de Recherche Des Cordeliers, Paris, France

#8 THE V-H^+-ATPase-a3-SUBUNIT CONTRIBUTES TO ELABORATE HIGHLY CALCIFYING ENAMEL DURING AMELOGENESIS

HARADA, H*, IDA-YONEMOCHI, H, SAHARA, Y, OHSHIMA, H, FUJIWARA, N, MATSUMOTO, N, NAKANISHI-MATSUI, M and OTSU, K

#9 EXPRESSION OF NKCC1 (SLC12A2) IN MOUSE AMELOBLASTS DURING AMELOGENESIS

JALALI, R*, ZANDIEH-DOULABI, L, MICHA, D, MELVIN, JE, CATALAN, MA and BRONCHERS, ALJJ

#10 Ca2+ RELEASE FROM THE ER VIA IP3RS IN AMELOBLAST

SORGE, JL*, NURBAEVA, ML, ECKSTEIN, M and LACRUZ, RS

#11 FROM CELLS TO MATRIX: ENAMEL ION TRANSPORT BY CLATHRIN-COATED VESICLES

PANDYA, M*, DANGARIA, S, GREENE, LE, and DIEKWISCH, TGH

#12 ENDOCYTIC PATHWAYS OF AMELOGENIN DURING ENAMEL FORMATION

PHAM, CD*, SIMMER, JP, HU, JC-C and CHUN, YP

#13 DO AMELOBLASTS TAKE A BREAK ON SUNDAY?

TAN, HXS, THIO, YLNA, YUAN, J, LIMBRI, F and HSU, CS*

#14 THE ADDED VALUE OF LNA PROBES FOR AMELOGENIN RNA DETECTION

HOTTON, D, LIGNON, G, ISAAC, J, ASSELIN, A, FAUTREL, A and BERDAL, A *

#8 THE V-H$^+$-ATPase-a3-SUBUNIT CONTRIBUTES TO ELABORATE HIGHLY CALCIFYING ENAMEL DURING AMELOGENESIS

HARADA, H[1*], IDA-YONEMOCHI, H[2], SAHARA, Y[3], OHSHIMA, H[2], FUJIWARA, N[1], MATSUMOTO, N[4], NAKANISHI-MATSUI, M[4] and OTSU, K[1].

[1]Division of Developmental Biology and Regenerative Medicine, Department of Anatomy, Iwate Medical University, 2-1-1, Nishitokuta, Yahaba, Iwate 028-3694, Japan; [2]Division of Anatomy and Cell Biology of the Hard Tissue, Department of Tissue Regeneration and Reconstruction, Niigata University Graduate School of Medical and Dental Sciences, 2-5274 Gakkocho-dori, Chuo-ku, Niigata 951-8514, JAPAN; [3] Department of Physiology, Iwate Medical University, 2-1-1, Nishitokuta, Yahaba, Iwate 028-3694, Japan; [4]Department of Biochemistry, School of Pharmacy, Iwate Medical University, 2-1-1, Nishitokuta, Yahaba, Iwate 028-3694, Japan

Email: hideha@iwate-med.ac.jp

OBJECTIVES: Ameloblasts build the hardest tissue in the body through various differentiation stages, but the mechanisms remain to be completely elucidated. Vacuolar proton-ATPase (V-H$^+$-ATPase) is a multi-subunit enzyme that regulates proton transport to create acidic microenvironment. It has been known that the maturation-stage ameloblasts express V-H$^+$-ATPase strongly and play a role of produce the acidic microenvironment at the surface of enamel matrix. Our aim is to reveal a role of a3-isoform of V-H$^+$-ATPase (referred to as an osteoclast type) during amelogenesis.

METHODS: We examined the expression pattern of a3-isoform by immunohistochemistry and a3-isoform-GFP mice, and carried out a3-isoform overexpression using rat ameloblast cell line (HAT7; maturation stage model, Bori, E., et al., J Dent Res, 2016) as a gain of function experiment. The teeth of epithelium-specific a3-isoform deficient mice were analyzed as a loss of function experiment.

RESULTS: The expression of ameloblasts was detected with low intensity compared with the intense expression of osteoclasts. The overexpression of a3-isoform induced acidic microenvironment at the basal side and led to some resorption pits on the apatite coat in culture dish. Further, epithelium-specific a3-isoform deficient mice exhibited amelogenesis imperfecta with white turbidity and crack in lower incisors.

CONCLUSION: The creation of acidic microenvironment is closely associated with a3-isoform expression by maturation-stage ameloblasts, and it may contribute to elaborate highly calcifying enamel at the maturation stage. In this paper, we will discuss the differentiation mechanisms from secretory to maturation stage, and further consider a role of acidic microenvironment for calcification during amelogenesis.

DISCUSSION:

Jim Simmer (Michigan): In your *in vitro* system, was a biomineralization process ongoing in the matrix?

Hidemitsu Harada (Iwate): Ameloblasts were in direct contact with the plastic dish. We did not test apatite-coated dishes.

Jim Simmer (Michigan): Under the ameloblasts in *vivo*, the formation of hydroxyapatite produces a huge amount of protons raising the question of generating acidity in the mineralizing enamel.

Hidemitsu Harada (Iwate): In the *in vitro* model, ameloblasts were in direct contact with the plastic dish. They were at the maturation stage, expressing KLK4, MMP20 and a small amount of matrix proteins.

#9 EXPRESSION OF NKCC1 (SLC12A2) IN MOUSE AMELOBLASTS DURING AMELOGENESIS

JALALI, R[1]*, ZANDIEH-DOULABI, L[1], MICHA, D[3], MELVIN, JE[4], CATALAN, MA[4] and BRONCHERS, ALJJ[1]

[1]Oral Cell Biology & [2]Functional Anatomy, ACTA, University of Amsterdam and VU- University of Amsterdam, Amsterdam, Netherlands; [3]Department of Clinical Genetics, Vrije Universiteit Medical Center, Amsterdam, Netherlands; [4]The Secretory Mechanisms and Dysfunction Section,NIDCR/NIH, Bethesda, MD, USA

Email: r.jalali@ACTA.NL

OBJECTIVES: Ameloblasts regulate the acid/base status of mineralizing enamel. It has been proposed that maturation stage ameloblast express a wide variety of membrane-associated ion channels and transporters to control pH and calcium homeostasis during enamel formation. Enhanced levels of sodium and potassium in hypomineralized enamel observed in *Ae2*-null and *Cftr*-null mice suggest a possible role for sodium and potassium recycling from the enamel layer. We tested the hypothesis that maturation-stage ameloblasts express Na:K:Cl cotransporter (NKCC1) to recycle sodium, chloride and potassium from enamel layer.

METHODS: Hemimandible from young adult *Nkcc1* null mice and wild type controls were collected, fixed in buffered formalin and processed into paraffin. After antigen retrieval in EDTA solution (pH 9.0, overnight, 60 °C) sections were incubated with primary antibody (goat anti-NKCC1). Freeze-dried enamel organs from lower incisors were used for micro-CT analysis and Western blotting.

RESULTS: Nkcc1 was immunolocalized in the outer enamel epithelium in early secretory stage of amelogenesis and in papillary layer at the maturation stage of amelogenesis. Mice with null mutation of *Nkcc1* had a normal dental phenotype without changes in mineral density in lower incisors as measured by micro-CT. Western blotting indicated that the amount of protein of the Cl^-/HCO_3^- exchangers SLC26A3/Dra and SLC26A6/Pat1 (members of solute carrier 26A family) and sodium bicarbonate cotransporter (Nbce1) were substantially increased in *Nkcc1* null mice.

CONCLUSION: The outer enamel epithelium and adjacent supportive enamel organ cells, but not maturation-stage ameloblasts, express Nkcc1 during amelogenesis. NKCC1 may transport of ions from connective tissue into the enamel organ to transport fluid and regulate osmotic pressure. Upregulation of Dra, Slc26a6 and Nbce1 in the *Nkcc1*- null enamel organ suggests that an imbalance in ion transport by enamel organ epithelium is compensated by upregulation of pH regulators. Furthermore, our results support the notion that the ameloblast and papillary layers cooperate to form dental enamel.

DISCUSSION:

Ariane Berdal (Paris): Did you relate your study to aquaporins or water transport?

Rozita Jalali (Amsterdam): There is one study reporting aquaporins in the ameloblasts but we couldn't find specific antibodies and so were unable to investigate this.

Pamela Den Besten (San Francisco): One of the biggest challenges in working with ameloblasts is cell culture systems. It seems that in your study, the papillary layer *in vivo* and the HAT7 cells *in vitro* were both positive. Would HAT7 cells be papillary layer cells?

Rozita Jalali (Amsterdam): Microarray data from your laboratory confirmed our data on NKCC1 and NKCC2. There is another study (Mori et al, Budapest), showing the bicarbonate transporter expressed in MRBC1 ameloblast cells *in vitro* as well as the papillary layer *in vivo*. It may be concluded that these transporters might be markers for enamel organ cells.

#10 Ca^{2+} RELEASE FROM THE ER VIA IP3RS IN AMELOBLAST

SORGE, JL*, NURBAEVA, ML, ECKSTEIN, M and LACRUZ, RS[1]

NYU College of Dentistry, New York, NY 10012, USA

Email: jls26@nyu.edu

OBJECTIVES: The endoplasmic reticulum (ER) of cells acts as a Ca^{2+} sink releasing Ca^{2+} into the cytosol as needed for various functions. Here we aim to determine whether the release of Ca^{2+} pools from ER uses inositol 1,4,5- trisphosphate receptor (IP3Rs) as the release channels in enamel cells.

METHODS: Firstly, Real Time (RT)-PCR and immunofluorescence were used to determine the expression of all three IP3R isoforms (IP3R1, IP3R2 and IP3R3). Cells of the mouse-derived ameloblast-like LS8 cell line were loaded with mag-fluo-4/AM, a fluorescent Ca^{2+} indicator with a low affinity for Ca^{2+}. LS8 cells were then permeabilised to release cytosolic Ca^{2+}, leaving mag-fluo-4/AM trapped within the ER. This enables us to gain experimental access to the ER Ca^{2+} pools and monitor Ca^{2+} release from this organelle. ATP was used as an agonist to initiate Ca^{2+} uptake into ER in the presence of a mitochondrial Ca^{2+} uptake inhibitor.

RESULTS: Using RT-PCR, transcripts of IP3R1, IP3R2 and IP3R3 were identified in LS8 cells and their expression was further confirmed by immunofluorescence. To examine the function of IP3Rs in our system, LS8 cells were loaded with mag-fluo-4/AM and permeabilised. Upon the addition of adenophostin A, which selectively activates IP3Rs in permeabilised cells, Ca^{2+} was released from the ER in LS8 cells.

CONCLUSION: In this study, we demonstrate that all the IP3R isoforms are endogenously expressed in LS8 cells and show that one or more of these isoforms enable Ca^{2+} release from the ER.

DISCUSSION:

Mike Hubbard (Melbourne): Do you think eventually we'll be able to probe the differences between the IP3 responses in the ER, smooth ER and tubular vesicular structures present in the ameloblasts? Would you have enough optical resolution?

Jessica Sorge (New York): The optical resolution is OK, but I am not so sure if this is possible with primary cells as they don't last long enough.

#11 FROM CELLS TO MATRIX: ENAMEL ION TRANSPORT BY CLATHRIN-COATED VESICLES

PANDYA, M[1 2]*, DANGARIA, S[3], GREENE, LE[4], and DIEKWISCH, TGH[1 2]

[1 2]Department of Periodontics and Center for Craniofacial Research and Diagnosis, Texas A&M College of Dentistry, 3302 Gaston Avenue, Dallas, TX 75206, USA; [3]University of Southern California, School of Dentistry, 925 W 34th St, Los Angeles, CA 90089, USA; [4]National Heart, Lung, and Blood Institute, Building 50 Room 2537, Bethesda, MD 20814, USA

E-mail: diekwisch@tamhsc.edu

Enamel formation involves the transport of high numbers of mineral ions across the ameloblast cell layer into the enamel matrix. This process requires complex packaging and efficient protective barriers to protect the intracellular environment from mineral toxicity.

OBJECTIVES: Based on earlier studies in amelogenin null (*Amel*$^{-/-}$) mice we have hypothesized that enamel mineral transport involves the intravesicular packaging of calcium and phosphate ions within an amelogenin matrix inside of clathrin coated secretory vesicles.

METHODS: Five days postnatal mouse molars from control and *Amel*$^{-/-}$ mice were prepared for co-immunoprecipitation and Western blots. For microscopic analysis, mineralized deposits from 5-7 days postnatal enamel organs were collected and subjected to immunohistochemistry, transmission electron microscopy and electron diffraction analysis.

RESULTS: Amelogenin and clathrin co-localized in ameloblast secretory vesicles via immunohistochemistry. When tested for protein-protein interactions, anti-amelogenin antibodies precipitated the 180kDa Clathrin protein. *Amel*$^{-/-}$ mouse molars displayed greatly reduced clathrin immunoreactivity and featured approximately 20 micrometer tall apatite pegs on the barren dentin surface and in other areas of the enamel organ. Intravenous Injection of the calcium indicator Fura-4 resulted in fluorescent label accumulation at the proximal ameloblast pole of amelogenin null mice, while Fura-4 label reached the enamel layer in wild-type counterparts after 24 hours. When compared on an ultrastructural level, *Amel*$^{-/-}$ ameloblast secretory vesicles lost their characteristic electron dense matrix, and mineral content was greatly reduced. Element mapping studies demonstrated increased calcium and protein content in wild-type secretory vesicles, further supporting our concept of ameloblast secretory vesicles as calcium ferries. Ameloblasts of mice lacking the clathrin-uncoating protein auxillin were characterized by voluminous vacuoles instead of secretory vesicles, with discrete amelogenin reactivity at the vacuole boundaries.

CONCLUSION: Together, these studies support our hypothesis that enamel mineral ions are transported through ameloblasts via clathrin-coated vesicles inside of which they are stored within an amelogenin-rich protein matrix.

<u>DISCUSSION:</u>

Jim Simmer (Michigan): We also looked at the amelogenin null mice and saw that the nodules in the molars were forming in the maturation stage. That's on my poster, do you care to comment?

Mirali Pandya (Texas): Our study showed that the clathrin is the one helping with the deposition. Our assumption is that cells probably would not survive to such high toxicity of these ions, there is a need for some kind of packaging for them to go through this layer and for ameloblasts to survive. The ectopic deposition we saw occurred during the early stages. I would assume that this is due to a cell transportation problem.

#12 ENDOCYTIC PATHWAYS OF AMELOGENIN DURING ENAMEL FORMATION

PHAM, CD[1]*, SIMMER, JP[2], HU, JC-C[2] and CHUN, YP[1]

[1]Department of Periodontics, School of Dentistry, University of Texas Health Science Center at San Antonio, USA; [2]Department of Biologic and Materials Sciences, School of Dentistry, University of Michigan, Ann Arbor, Michigan, USA

Email: phamc3@uthscsa.edu

Endocytosis is a critical event in amelogenesis to remove processed enamel proteins. Insufficient removal of enamel proteins due to disrupted endocytosis causes *Amelogenesis imperfecta*. Ameloblasts internalize cleaved enamel proteins via vesicles. However, it is unknown what endocytosis pathway is utilized following vesicle formation.

OBJECTIVE: The goal was to define the endocytic pathway of amelogenin *in vivo* and *in vitro*.

METHODS: Hemimandibles of wild-type PN5 and PN11 mice were fixated in 4% PFA by immersion, decalcified, embedded in paraffin and sectioned in sagittal direction. The tissue section of the first molar was incubated with anti-Rab5 and -Rab7, -TGN46 and -cathepsin K antibodies for immunolocalization and counterstained with methyl green. Images were taken under a light microscope. For *in vitro* experiments, full-length recombinant murine amelogenin (Amel) was expressed from pET179 in *E. coli* and purified as described previously (Simmer et al. 1994). Amel was labeled with Alexa-Fluor-488nm and added to enamel organ cells LS8 at a final concentration of 25 µg/ml at 37°C from 0-4 h. Cells were fixated in 2% PFA/0.2% Triton-X100, and incubated with the same antibodies. Alexa-Fluor-568nm labeled secondary antibody was used for fluorescence detection. Cells were mounted on Vectorshield/DAPI and visualized using laser confocal microscopy.

RESULTS: Immunostaining with anti-Rab5, -Rab7, -TGN46 and -cathepsin K, yielded positive reactions in the infranuclear region of ameloblasts at secretory and maturation stages *in vivo*. In LS8 cells, at 0h Amel-488 was diffusely spread throughout the cytosol, whereas at 2h and 4h Amel-488 progressively localized into distinct foci increasing in size and number. The formation of co-localized foci for Amel-488 and endosomal markers Rab5, Rab7, TGN46 and cathepsin K progressed in similar fashion.

CONCLUSIONS: Proteins involved in endocytosis localize to ameloblasts. Amel is routed to endosomal trafficking through early, late endosomes, trans-Golgi network and lysosomes.

ACKNOWLEDGEMENTS: This research was supported by the NIDCR grants K08 DE022800 (YPC) and T32 DE014318 (CDP).

DISCUSSION:

Ariane Berdal (Paris): Could you get some specific inhibitors to test which pathways are operating for each step?

Cong-Dat Pham (San Antonio): We could do that. We also want to test some other markers, like LAM1 and some others in the lateral membrane to see whether there is any transport into the other cells.

Wendy Shaw (Pacific Northwest National Laboratory) : Did you also look at the degraded proteins or did you just look at the whole amelogenin and did you see differences?

Cong-Dat Pham (San Antonio): We looked at the full amelogenin.

Wendy Shaw (Pacific Northwest National Laboratory) : Is it part of your plan to look at the differences? I would guess that they might be quite distinct.

Cong-Dat Pham (San Antonio): I have not tried that with degraded protein. For a spliced version of amelogenin (LRAP), I have found the same results. But I haven't presented that.

#13 DO AMELOBLASTS TAKE A BREAK ON SUNDAY?

TAN, HXS[1], THIO, YLNA[1], YUAN, J[1], LIMBRI, F[1] and HSU, CS[2]*

[1]Ministry of Health, 16 College Rd, Singapore, 169854; [2]Department of Dentistry :: a member of NUHS, National University of Singapore, 11 Lower Kent Ridge Road, Singapore 119083

Email: denhsus@nus.edu.sg

Striae periodicity refers to the exact number of cross-striations between successive Retzius lines. Academics have proposed that striae of Retzius denote periods of inactivity by ameloblasts, which occur approximately every 7 days, such that striae periodicities follow a median value of 7. While European and American studies have found variations in striae periodicity values, research comparing striae periodicities across different gender groups and populations in different geographical locations, particularly in the Asian context, is lacking.

OBJECTIVES: To compare the striae periodicities of the Heilongjiang Chinese and Singaporean Chinese population, as well as that of Singaporean males and females, in addition to their respective difference from the value of 7.

METHODS: 35 non-carious and non-restored permanent teeth from Singapore and 35 teeth from Heilongjiang, China were collected. Buccolingual sections with 150-180 µm in thickness were obtained from each tooth and polished down to a thickness of 80-100µm. Striae periodicities were measured using polarized light microscopy. The IBM SPSS Statistics 20 programme was used for statistical analyses using appropriate parametric and non-parametric tests.

RESULTS: A statistically significant difference between the striae periodicities of Chinese teeth from Heilongjiang and Singapore was found ($p<0.05$), and no significant difference between striae periodicities of Singaporean Chinese Female and Singaporean Chinese Male teeth ($p>0.05$). The 1-sample Wilcoxon Signed Rank test results showed that the combined striae periodicity of all samples followed a median value of 7 ($p>0.05$), but the striae periodicities of individual geographical locations deviated from the median of 7 ($p<0.05$).

CONCLUSION: Enamel periodicities vary according to geographic locations, but within the same geographic location, striae periodicity does not vary with gender. In general, striae periodicities follow a median value of 7.

DISCUSSION:

Jason Wan (Bethesda): Have you looked at other environmental factors, like geographical location and sex?

Stephen Hsu (Singapore): When we chose the location, our underlying speculation was environment and lighting would be important factors. Maybe diet too, although we tried to restrict the population to Chinese people. Singapore probably has a more westernised diet. We require further clarification and this is just the beginning of the journey. We tried to look at the bigger picture first.

Pamela Den Besten (San Francisco): They were beautiful images. We were recently looking at the effects of stress on tooth and enamel formation and found *in utero* effects of maternal stress on the thickness of enamel. We are interested in looking at the periodicity of the lines as a sort of biomarker of stress. Do you think there are any differences related to the behaviour stress in these two populations?

Stephen Hsu (Singapore): My speculation is that ameloblasts, like you and me, react when they are stressed but they all go with the same pattern. I would imagine that the daily products of 3-4 microns between the cross striation would be shorter, narrower (2.5 or 2.9) under stress. For the rhythm, this is actually an environmental or genetic program through lighting, hypothermia or metabolic variations. Amongst these factors, we don't know which one is predominant. I guess that the stress would reduce their output and so the distance between two cross-striations may be shorter. However, their number should remain quite constant.

Raquel Gerlach (Sao Paulo): I was wondering if you had looked back into archaeological data, using these lines to count daily rhythms?

Stephen Hsu (Singapore): Based on a literature review, there was a wide range of mean values. In general, it seems to be in this range around 6 to 12 microns. The methodology may easily miss one cycle. For values of 12-14 microns, one band or a group of enamel prisms may be outside of the focus depth. In general, the newest technologies such as micro CT and synchrotron studies would help much.

#14 THE ADDED VALUE OF LNA PROBES FOR AMELOGENIN RNA DETECTION

HOTTON, D[1], LIGNON, G[1], ISAAC, J[1], ASSELIN, A[1], FAUTREL, A[2] and BERDAL, A[1] *

[1]Molecular Oral Pathophysiology, Cordeliers Research Center. UMRS 1138 INSERM. Paris-Descartes, Pierre-et-Marie-Curie, Paris-Diderot Universities, 15 rue de l'Ecole de Médecine, 75006 Paris, France ; [2]Plateforme d'histopathologie H2P2. UMR991 INSERM, University Rennes 1, 2 avenue du Pr Léon Bernard, 35043, Rennes Cedex, France

Email: ariane.berdal@crc.jussieu.fr

OBJECTIVES: Mineralized samples require stringent preparation process for histology that extract tissue peptides and RNAs. In order to increase probe affinity and specificity for *in situ* hybridization (ISH), Locked Nucleic Acids (LNA), which contain one internal methyl bridge between 2'O and 4'C have been designed. Already shown efficient for micro RNAs, LNA probes were tested here for amelogenin (AMELX) mRNAs.

METHODS: RTqPCR was done on microdissected cells to classify them based on their expression levels. AMELX and scramble (20-nucleotides) LNA probes were tagged with digoxigenin and visualized either with NBT/BCIP (alkaline phosphatase) or fluorescence tags (rhodamine). Manual or automated ISH was performed on routine histological sections (decalcification, paraffin-embedding of jaws from C57Bl6 3-30 days mice).

RESULTS: LNA labelling levels were: secretion-stage ameloblasts ~ odontoblasts > maturation ~ protection-stage ameloblasts ~ osteoblasts > cementoblasts ~ first third of epithelial root sheath > the rest of Hertwig epithelial sheath = controls (scramble, AMELX LNA in AMELX KO mice) = no signal. Dental ligament expression levels varied.

CONCLUSION: LNA probes provided the cellular distribution of AMELX RNAs corresponding to previous RTqPCR data on native microdissected cells, confirmed on laser captured (Jacques - PLos One, 2014) and FACS-sorted cells (Isaac, unpublished). Fluorescent 20 nucleotide-LNA probes will give access to mRNA (co)localization and subcellular distribution. Cell signaling functions of dental peptides, widely accepted in regenerative medicine and *in vitro*, are obscured by a lack of information on their native *in vivo* expression pattern in enamel and jaw. The underlying regulatory pathways might involve multiple cell cross-talks including biomechanical constraints (Amri - Am J Pathol, *in press*).

No Questions

PANEL DISCUSSION:

Bernhard Ganss (Toronto): I want to raise a general question since we've been talking about transport phenomena through and in the ameloblasts. How much of these transport phenomena do we think is happening inter-cellular versus intra-cellular, through or in between cells? The other question is, is there any relationship between these transport phenomena and smooth- and ruffle-ended border ameloblasts?

Hidemitsu Harada (Iwade): This is a very difficult question. HAT7 cell cultures show two types of ameloblasts: ruffle-ended and smooth-ended types. In confluent cultures, Alizarin red staining may be either intra-cellular or between the cells. I think that at the maturation stage, two types of calcifications may be present, with intra-cellular carrier transport for ruffled-ended ameloblasts, and para-cellular transport for smooth-ended ameloblasts. This is my hypothesis using the data on HAT7 cells.

Rozita Jalali (Amsterdam): Our group, (published in Bone, 2012) evidenced that the lysosomal, T-cell, immune regulator 1 (Tcirg1, v-H-Atp6v(0)a(3)), which is an essential part of the plasma membrane proton pump in the osteoclasts, is not present in the ruffled border of the maturation stage ameloblasts, and ameloblasts are unaffected in the Tcirg1 null mutant. In the ameloblasts, the ruffled borders could play a role in the intra-cellular regulation of ion transportation. Regarding the second question about the ion transportation between the cells, we do have the stratum intermedium and ameloblasts in the secretory stage, then papillary layer and ameloblasts in the maturation stage. These two layers are working together, as concluded from my NKCC1 results. I'm going to work with a patch clamp system to see how they interact with each other.

Mirali Pandya (Dallas): I would like to comment on whether we feel it is intra-vesicular or between the cells. There are a lot of intra-vesicular coated vesicles involved in the transportation of these mineral ions from the blood vessels to the enamel layer. There is a whole lot going through the cell and within the cell. There is a lot of capability to carry on the transportation instead of associating with other cells. There might be many pathways but we think this is one of the major pathways.

Derk Joester (Northwestern): I have a question regarding the ion transport. If I understood correctly, what you proposed is that essentially calcium is taken up in these clathrin-coated vesicles and phosphate, at blood plasma level. Is that accurate?

Mirali Pandya (Dallas): Yes that is correct.

Derk Joester (Northwestern): So that would mean that you transport actually at a low concentration. From a chemist's perspective, if there's no concentration step, you would assume that you have to carry a huge amount of water with these ions to the mineralisation area and you'd have to carry all that water back and a lot of other ions as well, so I wondered what your thoughts were about that. It seems like a terribly inefficient way of going about things.

Mirali Pandya (Dallas): We feel that this is a whole assembly of not just the coated-vesicles but there are a lot of proteins and they all work together. They form a lattice that covers the ions and the minerals inside and it does not lead to toxicity for these cells.

Derk Joester (Northwestern): I understand that part of the story but what I don't understand is what the advantage would be of using such low concentrations, so I would rather challenge that and say it doesn't seem like a good idea to be constantly transferring calcium and phosphate at these low concentrations. It would make much more sense, intuitively to me, if there was a concentration step that would bump up the concentration, not just at the mineralisation site.

Mirali Pandya (Dallas): I think there are a lot of ions that are being carried in these clathrin-coated vesicles. We have some data from inside these vesicles, there was a high concentration of calcium.

Derk Joester (Northwestern): So the concentration was higher than in the source?

Mirali Pandya (Texas): Yes, in the wild type mice we saw there was a higher concentration than in the amelogenin null mouse.

Mohammed Al-Mosawi, (QMU): My question is about the talk entitled: 'Do ameloblasts take a break on Sunday?'. It was mentioned that the Striae of Retzius are the product of ameloblast inactivity. How certain are we that the ameloblast inactivity is the cause rather than a slowed (reduced) activity of ameloblasts? Second question, the literature suggests that ameloblasts slow down as they get older. Does this affect the Striae of Retzius periodicity? And, if it does, do you take this into consideration?

Stephen Hsu (Singapore): We sleep, we don't die and resurrect. The ameloblast output reduced, so you can see a band, implying that the crystal formation, either quality or quantity, is reduced depending on the time of day. Animal studies evidenced shorter cross-striations; the band is still there, indicating the slow-down of ameloblast activity. So, the answer to the first question is yes, they continue functionally, as the quality and quantity of their output reduces. The second question was about the site-specific periodicity. We specifically selected the outer enamel that is between the middle and the cervical third of the tooth crown. Knowing these site-specificities, we targeted this region. To speculate whether there is any ageing effect in the ameloblasts during the formation of the first inner and the outer enamel, we do see differences. Most of us know that the chemical composition of the inner enamel is very different from the outer enamel and perhaps also the crystal quality and orientation; whether it's a sign of ageing I'm not sure. As a novice in this area I am curious, but I can see a big difference.

Kaushik Mukherkjee (Southern California): Can anyone explain or comment on the signs of intra-cellular amelogenin aggregation in vesicles under physiological conditions and its significance *in vivo*. Can I just add a quick follow-up? I asked that because in some of the mutant phenotypes, it is this very intra-cellular aggregation that affects the ameloblasts and causes apoptosis. So if it's

something that's happening under physiological conditions, in one case you have regular functioning and in another cases, ameloblasts would go through apoptosis.

Tom Diekwisch (Dallas): We've found good evidences to reply to your question and which also explain Dr Simmer's concerns about the fate of these minerals. It seems like these ions are bound to amelogenins, incredibly well bound, in these secretory vesicles. The secretory vesicles are incredibly well organised and packaged to host high concentrations of ions. There must be a mechanism to concentrate the ions that come from the blood vessels into these vesicles. Once we look at the vesicles, these ions are not all over the ameloblasts, causing stress and toxicity. They are packaged, finely and very precisely packaged, in the centre of these vesicles. These vesicles are coated with a clathrin membrane and a number of other proteins in a way that it may be almost impossible for these ions to leak until they are secreted. If we block amelogenin, what happens is these deposits are not formed all at once, they are formed step-by-step. So in the amelogenin null mice, you don't see these packed like deposits all at once but they are continuously growing. In mice, from the early secretory stage, you have fine granules and then they fuse into these big packs like you see here at a later stage of the secretory ameloblasts.

The answer to the question is: these ions are wonderfully packaged in these vesicles and we have good evidences for that.

Unknown: Coming from a different field, I would say that there are actually several different pathways. We shouldn't exclude, for example, the exclusion pathway or calcium signalling pathway. There is a balance between the two, it's not just this pathway, there are also others. There are different pathways to think about and not just one.

Kei Katsura (San Francisco): Most of the mechanisms that we're looking at are calcium uptake/ deposition and endocytosis, viewed as two separate mechanisms. But do you have any evidence suggesting that there's calcium driving endocytotic events that occur in other cell types or if it's something where endocytosis is driving calcium deposition or uptake? For example, in the neuronal cells you have calcium driving endocytotic events and vice versa and it would be interesting to look at what really drives modulation.

Unknown: I haven't looked at it so I cannot comment.

Sherif Elsharkawy (QMU): I have a question for Dr Hsu about cross-striations. You've looked at the same ethnic group in different geographical locations and found significant differences. What is interesting is that those two groups are living in different geographic locations: up to 30-1000 metres above sea level in the case of the first group while the Singapore group is no more than 15 metres above sea level. So I think there's something to do with atmosphere or atmospheric pressure that makes a lot of stresses for enamel formation. It would be really interesting to look at the effect of altitude and a comment would be much appreciated.

Stephen Hsu (Singapore): Thank you for the challenge. My intuitive speculation looking at these two locations is that there are many contributing environmental factors: one of the factors you mentioned is the altitude - and also light - the majority of the time in Singapore you have sunlight, you don't have winter. So if you ask me, there is a real statistical difference between the two populations, but whether this statistical difference really is clinically relevant is questionable, because the difference is quite actually small, and it could be moderated by those factors that you just mentioned. This is the beginning, as although they are both Chinese, the populations are different in socioeconomic status and Singapore's economy is different from other areas. We assumed that the teeth collected are from Chinese people, and in Singapore we did ask the dentists to specifically collect for us from their Chinese patients. But I don't think this plays a major part. More probably differences in biological genetics and epigenetics are important, but your comments on the statistical difference, yes they are different and it's an interesting point as to whether they are clinically relevant.

Session 3: Biomineralisation And Biomimetics

Moderator: Janet Moradian-Oldak, University of Southern California, USA

#15 AMELOGENIN, ENAMELIN, AMELOBLASTIN, AND EARLY AMELOGENESIS

SIMMER, JP*, SMITH, CE, HU, Y and HU, JC-C

#16 SUBUNIT STRUCTURES IN DEVELOPING ENAMEL CRYSTALS

ROBINSON, C* and CONNELL, SD

#17 POLYMER-INDUCED LIQUID PRECURSOR (PILP) APPLIED TO AMELOGENIN NANORIBBONS

CARNEIRO, KMM*, NURROHMAN, H, BONDE, J and HABELITZ, S

#18 AMELOGENIN-INSPIRED PEPTIDES FOR ENAMEL MIMETICS

MUKHERJEE, K*, RUAN, Q, CHAIDEZ, E and MORADIAN-OLDAK, J

#19 FABRICATION OF HIERARCHICALLY-ORDERED ENAMEL-LIKE APATITE: MOLECULAR AND DIFFUSION MECHANISMS OF FORMATION

ELSHARKAWY, S, AL-JAWAD, M, MEHTA, K, AGARWAL, S, TEJEDA-MONTES, E, WILSON, RW, STEVENS, MM, ANDERSON, P and MATA, A

#20 ELASTIN-LIKE PROTEIN MEDIATED CALCIUM PHOSPHATE FORMATION – TOWARDS IN SITU ENAMEL REGENERATION

SHUTURMINSKA, K*, ANDERSON, P, BUSHBY, AJ, MATA, A, AZEVEDO, H and AL-JAWAD, M

#21 ENAMEL MATRIX CONFIGURATION CHANGES DURING MINERAL TRANSPORT, NUCLEATION AND GROWTH

YAN, X, JIN, T, DANGARIA, SJ, ALLEN, M, LUAN, X, and DIEKWISCH, TGH*

#22 TOWARDS A 4-D MODEL OF HUMAN DENTAL ENAMEL BIOMINERALISATION

AL-MOSAWI, M*, DAVIS, GR and AL-JAWAD, M

#15 AMELOGENIN, ENAMELIN, AMELOBLASTIN, AND EARLY AMELOGENESIS

SIMMER, JP[1]*, SMITH, CE[1 2], HU, Y[1], HU, JC-C[1]

[1]Department of Biologic and Materials Sciences, University of Michigan School of Dentistry, 1210 Eisenhower Place, Ann Arbor, MI 48108; [2]Facility for Electron Microscopy Research, Department of Anatomy and Cell Biology, Faculty of Dentistry, McGill University, Montreal, Quebec H3A 0C7, Canada

Email: jsimmer@umich.edu

OBJECTIVES: To better characterize enamel formed in the *Amelx*, *Enam*, and *Ambn* null mice to better determine their roles in mineral ribbon deposition and extension.

METHODS: C56BL/6 null mouse molars and incisors were characterized using dissecting, light, transmission electron, focused ion beam, and scanning electron microscopy (SEM), backscattered SEM, immunohistochemistry, nanohardness testing, and X-ray diffraction.

RESULTS: Demonstrated that *Enam* and *Ambn* are absolutely required for the deposition of enamel mineral ribbons. Ultrastructural analyses determined that dentin mineral forms normally in the *Enam* and *Ambn* null mice. Despite the progressive thickening of the dentin mineral layer, ameloblasts deposit no enamel ribbons and remain in contact with the increasingly mineralized dentin surface. The *Ambn* null mice deposit a thick globular layer of organic material (presumed to be amelogenin) on the dentin surface, but this layer does not initially mineralize and seems to diminish as the ameloblasts become increasingly pathological. The *Enam* null appears similar to the *Ambn* null, without the build-up of organic matrix. No ribbons form. Enamel mineral ribbons form in the *Amelx* mice but, following deposition of the initial enamel, inter-rod growth sites do not extend interproximally to define a Tomes process, the matrix does not thicken properly, and the enamel ribbons fuse into plates that radiate to the enamel surface. *Amelx* heterozygous mice produce enamel of variable thickness and hardness, which can be explained by lyonization.

CONCLUSION: Enamelin and ameloblastin are necessary for enamel ribbon formation and ameloblast integrity. Amelogenin does not lengthen the mineral ribbons, but its continued secretion and accumulation expands the extracellular matrix, which is necessary for sustained crystal elongation, thickening the enamel layer, and for proper ameloblast function. Amelogenin separates the enamel ribbons, inhibits their fusion, and regulates their conversion into hydroxyapatite.

ACKNOWLEDGEMENTS: This study was supported by NIDCR/NIH grants DE-015846 and DE-012769.

DISCUSSION:

Elia Beniash (Pittsburgh): This FIB (Focused Ion Beam) is a great technology, and it is really fascinating. Have you thought about correlative microscopy with these types of techniques, e.g. something like confocal microscopy, and then on the same samples, using the FIB.

Jim Simmer (Michigan): That's not really our focus right now. We've looked at a lot of samples and we'll do some grant writing and then we will be looking at trying to increase the complexity.

Wendy Shaw (Pacific Northwest National Laboratory): I'm curious, can you look even closer to see if there is any intertwining between the dentine and the enamel, or does it still look like there is a distinct junction?

Jim Simmer (Michigan): It's a distinct junction where you can clearly see that this is enamel and that this is dentine. The dentine is much more dense before the enamel starts forming on it. And the enamel ribbons that form are not nearly as dense and you can see that it is not a smooth line, there is an ameloblast finger-like process that goes into the surface. They start a group of ribbons growing right on the mineralised collagen. It would be two tissues that are clearly separate but they overlap spatially, so that it's almost like it's interlocking.

Wendy Shaw (Pacific Northwest National Laboratory): At the end of the movie you could see these large dark features moving through in between the cells. What is that?

Jim Simmer (Michigan): That is something that people have seen many times in the past, associated more with tissues that are a little more well-fixed. I think that the size of the interproximal protein accumulations are actually large in those and are seen in TEMs as well. Immuno-gold and TEM has shown that there is a lot of amelogenin and some ameloblastin in these accumulations, so what it highlighted is that there appears to be secretion of these proteins interproximally and that these things seem to work their way into the enamel at the inter-rod process. There are ideas on what they can do to help form enamel, but these are ideas and there is no work done on this yet.

#16 SUBUNIT STRUCTURES IN DEVELOPING ENAMEL CRYSTALS

ROBINSON, C[1]* and CONNELL, SD[2]

[1]School of Dentistry University of Leeds, Leeds, LS2 9LU, UK; [2]School of Physics and Astronomy, Molecular and Nanoscale Physics Group, EC Stoner Building, University of Leeds, Leeds, LS2 9JT, UK

Email: c.robinson@leeds.ac.uk

INTRODUCTION: Investigations of developing enamel crystals using Atomic Force Microscopy (AFM) revealed a subunit structure of 30-50 nm collinear swellings on crystal surfaces[1]. 30-50 nm structures were also seen using freeze etching[2]. AFM studies of mature enamel, suggested that these contained smaller~10-15 nm units perhaps an imprint of crystal initiation sites[3].

OBJECTIVES: The investigation examined original freeze etched images at high resolution to see if the smaller subunits observed using AFM in mature enamel were present in developing enamel i.e. before loss of the organic matrix.

METHODS: For freeze etching[2], developing rat enamel was rapidly frozen, fractured under vacuum, ice sublimed from the fractured surface which was shadowed with gold and the metal replica subjected to high resolution TEM. For AFM[3], high-resolution tapping mode of human mature enamel sections was performed in air under ambient conditions.

RESULTS: High resolution freeze etching images of very early enamel showed a disordered arrangement of ~30-50 nm spherical structures with no obvious smaller 10-15 nm units. However, when linear arrangements of 30-50 nm units were visible, smaller ~10-15 nm units were observed.

CONCLUSIONS: Structures ~10-15 nm in diameter were detected in developing enamel. These were most evident when the 30-50 nm structures were in linear arrays. Formation of linear arrays of ~30-50 nm subunits may be associated with the development of mineral initiation sites and attendant processing of matrix proteins.

[1] Kirkham J, et al 1998. Connect Tiss Res: 38(1-4), 91-100; [2] Robinson C, et al 1981. J Cryst. Growth: 53, 160-165; [3] Robinson C, et al. 2006. Eu J Oral Sci: 114 (Suppl 1), 99-104.

DISCUSSION:

Elia Beniash (Pittsburgh): It's great, one question: what do you think about the fact that the ribbons at this 15 nanometer stage are anisotropic in all three dimensions?

Colin Robinson (Leeds): If we look at your paper (2010), if you look at a lot of those images carefully they are not *flat* ribbons, many of them actually have 15 nm corrugations on their surfaces. I think these are the same 15 nm subunits. I think the 15 nm units are clustered, and may fuse into a ribbon.

Elia Beniash (Pittsburgh): I'm talking about the previous stage, the 15 nm, not the 50 nm sub-units. Even at the 15 nm size they look like wide ribbons. So why are they like this?

Colin Robinson (Leeds): I think what you are saying is, do the sub-units all string together? I think they do but I think it happens a little bit later. I think it fits the data quite well. Also I think Tom Diekwisch has also shown 15 nanometer units in an earlier publication.

#17 POLYMER-INDUCED LIQUID PRECURSOR (PILP) APPLIED TO AMELOGENIN NANORIBBONS

CARNEIRO, KMM[1]*, NURROHMAN, H[2], BONDE, J[3] and HABELITZ, S[2]

[1]School of Dentistry, University of Toronto, Toronto, M5G 1G6, Canada: [2]School of Dentistry, University of California, San Francisco, 94158, United States; [3]Division of Pure and Applied Biochemistry, Center for Applied Life Sciences, Lund University, Lund, Sweden

Email: karina.m.m.carneiro@gmail.com

OBJECTIVES: The mechanism guiding tooth enamel mineralization has not been fully described, in part because of the complex and transient nature of the organic matrix during tissue maturation. Previously we reported the *in vitro* self-assembly of recombinant human amelogenin (rH174 and rH146) into amyloid-like nanoribbons. Such ribbons were not able to induce mineralization, suggesting that modifications to amelogenin and/or interactions with other groups may be required for guided crystal growth *in vitro*. Here we investigate the ability of the polymer-induced liquid precursor (PILP) method, which has been used for intrafibrillar collagen mineralization, to promote calcium phosphate mineralization in association with amelogenin supramolecular structures.

METHODS: Amelogenin nanoribbons were assembled and subsequently incubated for up to 3 days with varying concentrations of polyaspartic acid (pAsp) in the presence of calcium and phosphate ions (33 mM $CaCl_2$ and 21 mM KH_2PO_4). Self-assembly and mineralization of full-length amelogenin (rH174) and C-terminus modified amelogenin (rH146 and rH174+9) were characterized by atomic force microscopy (AFM) and transmission electron microscopy (TEM).

RESULTS: In the presence of varying concentrations of pAsp, amorphous calcium phosphate aggregates were observed in association with individual amelogenin ribbons. Growth of apatite nanocrystals occurred within rH174 ribbons and bundles of nanoribbons of rH174+9. Acicular apatite crystals formed along protein ribbons, suggesting templated mineral growth. In contrast, the amelogenin lacking the hydrophilic C-terminus did not interact with the pAsp droplets.

CONCLUSION: This study demonstrates that PILP may function in conjunction with amelogenin superstructures in a similar fashion as it does with collagen fibrils to produce an amorphous mineral precursor that transforms into aligned apatite. Our results suggest that non-amelogenin proteins may interact with amelogenin nanoribbons to guide mineralization of apatite nanofibers in developing enamel *in vivo*.

ACKNOWLEDGEMENTS: NSERC postdoctoral fellowship to KC, NIH/NIDCR R21DE023422 to SH and The Swedish Foundation “O.E. ock Edla Johanssons vetenskapliga stiftelse” to JB.

DISCUSSION:

Henry Margolis (Forsyth): I noticed in your abstract that you tried this with a truncated amelogenin without the C-terminus and that this did not do this with PILP. Can you comment?

Karina Carneiro (Toronto): We tried with the recombinant rH146 and the rH163, the information is in my poster. So with the rH146 we have not seen any mineralisation. The reason for that is that

where the proteins have C-terminus charges we can induce bundling, so you probably need a certain concentration of PILP droplets on top of the protein, whereas in the case of the 146 it does not form the bundles. You can see the droplets on top of the individual ribbons. We have shown that if you have pH at around 7 you will see with the 174 some crystals forming on top of it but these are not everywhere as you see it here. That’s where the PILP process is helping with the mineralisation.

Maisoon Al-Jawad (QMU): You mentioned you incubated your samples for three days. I wondered, if you tried a different time point or if you left it for longer, would you see the amorphous phase transform?

Karina Carneiro (Toronto): Actually the image that I’m showing is just with 20 minutes incubation with the PILP and the amelogenin nanoribbons, so in this case, the amelogenin nanoribbons are already formed. I then add the PILP droplets on to my amelogenin nanoribbons, so overtime you will see the mineralisation happen. If you incubated for a longer period of time it gets messy because you start to see some precipitation, but I’m not showing any of that data here. 20 minutes seems to be the magic number for us so we are able to get mineralisation.

#18 AMELOGENIN-INSPIRED PEPTIDES FOR ENAMEL MIMETICS

MUKHERJEE, K*, RUAN, Q, CHAIDEZ, E and MORADIAN-OLDAK, J

Institution: Center for Craniofacial Molecular Biology, Division of Biomedical Sciences, Herman Ostrow School of Dentistry, University of Southern California, Los Angeles, CA 90033, USA

E-mail: kmukherj@usc.edu

The predominant enamel matrix protein amelogenin self-assembles to control crystal orientation, elongation and packing, forming structured arrays of carbonated hydroxyapatite crystals during enamel biomineralization.

OBJECTIVE: To rationally design amelogenin-inspired peptides with active functional domains and test their potential in regulating the oriented growth of enamel-like crystals *in situ*.

METHODS: Two amelogenin-derived peptides (P26 and P32) were designed based on the identification of active apatite-binding and mineralization kinetics-promoting domains. The mineralization efficacy of the synthetic peptides in controlling the formation of ordered bundles of apatite crystals was compared with that of leucine-rich amelogenin peptide (LRAP) on demineralized enamel surfaces. Human third molars were sectioned longitudinally into 2 mm-thick tooth slices. To mimic carious lesions, the slices were immersed in a demineralizing solution (pH 4.6) at 37 °C for 2 hours. Peptides (P26, P32, and LRAP) were applied to the demineralized windows and the slices were placed in artificial saliva (pH 7) for 3-7 days. The morphology, composition and mechanical properties of newly formed crystals were characterized using SEM, XRD and Vickers microhardness tests respectively.

RESULTS: SEM revealed a dense homogeneous mineralized layer composed of needle-like *de novo* crystallites after 3 days, which matured to a more robust, well-oriented apatitic layer (10-15 µm thick) in 7 days. The regrown crystals assembled perpendicularly to the enamel surface and consolidated into a tight interface with the underlying native enamel. XRD displayed prominent diffraction peaks for hydroxyapatite that indicated good crystallinity of the new layer with a preferred c-axial crystal orientation (002 peak). The microhardness values of the synthetic peptide-treated tooth samples were striking: comparable to those achieved with the natural peptide (LRAP) and significantly higher than those of conventional fluoride-treated tooth samples.

CONCLUSION: Our results demonstrate that amelogenin-inspired peptides hold a promising potential in the development of durable, biomimetic, complex apatite microstructures for enamel repair and engineering.

<u>DISCUSSION:</u>

Derk Joester (Nothwestern): I have a quick question about the regrown crystals on the enamel surface. Can you talk a little bit about the morphology of the regrown crystallites on the enamel surface?

Kaushik Mukherjee (Southern California): They are needle-like, fine apatite crystals. In some of our

previous studies, we have shown an inside view and its attachment to the underlying enamel using FIB TEM. It's fine, needle-like apatite crystals with a preferred orientation.

Derk Joester (Nothwestern): How big are the crystallites?

Kaushik Mukherjee (Southern California): The thickness we have shown is up to 10 microns.

Derk Joester (Nothwestern): Individual crystallites?

Kaushik Mukherjee (Southern California): We haven't characterised individual crystals**.**

#19 FABRICATION OF HIERARCHICALLY-ORDERED ENAMEL-LIKE APATITE: MOLECULAR AND DIFFUSION MECHANISMS OF FORMATION

ELSHARKAWY, S[1 2 3], AL-JAWAD, M[3 4], MEHTA, K[2], AGARWAL, S[5 6], TEJEDA-MONTES, E[2], WILSON, RW[2 4], STEVENS, MM[5 6], ANDERSON, P[1 3] and MATA, A[1 2]

[1]Institute of Bioengineering, Queen Mary University of London, London E1 4NS, UK; [2]School of Engineering and Materials Science, Queen Mary University of London, London E1, UK; [3]Institute of Dentistry, Barts and The London School of Medicine and Dentistry, Queen Mary University of London, London E1 4NS, UK; [4]Materials Research Institute, Queen Mary University of London, London E1 4NS, UK; [5]Department of Materials, Imperial College London, London SW7 2AZ, UK; [6]Department of Bioengineering, Imperial College London, London SW7 2AZ, UK

E-mail: s.a.a.elsharkawy@qmul.ac.uk

OBJECTIVES: Dental enamel's distinctive hierarchical organization generates its remarkable mechanical properties, and critical role in tooth function (1,2). The aim of this study was to grow synthetically via organic matrix-mediated biomineralization a hierarchical apatite material that mimics the hierarchical structure of enamel.

METHODS: Cross-linked elastin-like polypeptides (ELPs) containing the analog of the 15 amino acid N-terminal residue of statherin (STNA15) were used as an organic scaffold to guide mineral growth. STNA15-ELP was cross-linked into membranes that were incubated in a supersaturated solution with respect to apatite at near-physiological conditions. The degree of cross-linking was varied with respect to the lysine content. The hydrodynamic radii, zeta potential, and secondary structure of the cross-linked ELP-STNA15 molecules were characterized via dynamic light scattering (DLS) and circular dichroism (CD) with and without the presence of calcium ions. Mineralised samples were investigated using a comprehensive suite of microscopy techniques (SEM/TEM) and chemical analyses (EDX/XRD/NMR/FTIR).

RESULTS: At optimum crosslinking amounts, we observed through microscopy and chemical analyses that ionic diffusion takes place within the bulk of the membrane where crystal nucleation occurs and grows into hierarchically-ordered apatite structures closely resembling those within dental enamel. The structures consist of elongated apatite nanorods ~85±22 nm thick organized into enamel-like prism microstructures ~3.8±0.9 µm thick that assemble to cover macroscopic areas. At the molecular level, STNA15-ELP, above its transition temperature (23 °C), exhibits β-spiral conformation at the expense of random coils. This would allow the hydrophilic sequences that possess negative charge to be presented, thus we observed higher calcium binding contributing its ability to organize the direction of growth of apatite within the membrane.

CONCLUSION: By employing a controlled molecular scaffold and diffusion parameters, we have grown complex hierarchically-ordered apatite structures that resemble those found in human dental enamel both chemically and morphologically at multiple length scales.

1. A. Boyde: Microstructure of enamel. CIBA Foundation Symposia (205), 18-31 (1997); 2. S. N. White, W. Luo, M. L. Paine, H. Fong, M. Sarikaya and M. L. Snead: Biological organization of hydroxyapatite crystallites into a fibrous continuum toughens and controls anisotropy in human enamel. J Dent Res, 80(1), 321-326 (2001)

DISCUSSION:

Janet Moradian-Oldak (Southern California): Is this something that you tried in *in situ* experiments and are you considering it as a bulk material, what is the thickness that you can get?

Sherif Elsharkawy (QMU): Right now, we are getting thickness of up to 30 microns, so this is still not bulk material, but we are in the process of developing the diffusion technique. With understanding of the scientific basis we can then develop bulk material, but it's a very thin coating for now. We tried some *in situ* but I haven't presented that, we are focusing on *in situ* on dentine tubules and on natural enamel as well.

Janet Moradian-Oldak (Southern California): You don't know what happens at the interface? Do you have a good attachment?

Sherif Elsharkawy (QMU): We looked at the interface with dentine. There are crystals that can infiltrate within the dentine tubules so we found that these coatings can cover an even surface and infiltrate the dentinal tubules quite well.

Wendy Shaw (Pacific Northwest National Laboratory): Do you think the rods that grow on the inside of the membrane; are those hydroxyapatite or amorphous phase and do they change over time?

Sherif Elsharkawy (QMU): I haven't looked at the rods with time, but they are hydroxyapatite by the end of the mineralisation. Different studies show there is a brushite phase which transforms to fluorapatite crystals at the early stages, for a few hours from mineralisation these start to transform. They absorb out to form this more acidic acid resistance phase, which is hydroxyapatite.

#20 ELASTIN-LIKE PROTEIN MEDIATED CALCIUM PHOSPHATE FORMATION – TOWARDS IN SITU ENAMEL REGENERATION

SHUTURMINSKA, K[1 2]*, ANDERSON, P[1], BUSHBY, AJ[2], MATA, A[2], AZEVEDO, H[2] and AL-JAWAD, M[1]

[1]DPSU, Barts and The London School of Medicine and Dentistry, London, E1 4NS; [2]SEMS, Queen Mary University of London, London, E1 4NS

Email: k.shuturminska@qmul.ac.uk

OBJECTIVES: Elastin-like proteins (ELPs) have been produced and used for bone regeneration. ELPs containing the 15 amino acid N-terminal residue of statherin (STNA15) are of interest since salivary statherin is associated with enamel remineralisation. Here we explore the influence of STNA15-ELP conformation on hierarchical apatite formation.

METHODS: Effects of protein conformation were studied by comparing a) STNA15-ELP (0.1 mg/ml) coating on borosilicate glass, b) STNA15-ELP (0.1 mg/ml) in solution and c) no protein, all in a fluoridated mineralising solution; incubated for 8 days. Protein conformation and mineral morphology / composition were characterised by FTIR, SEM and EDX.

RESULTS: In condition a), flat platelets form (~50 µm length with ~500 nm thickness) and in condition b) nano-scale needle-like morphologies with an abundance of spherical shapes are observed. In condition c) no apatite formation is observed. EDX shows the platelets to have Ca:P ratio 1.65 indicative of apatite, however for protein incubated directly in solution, calcium deficient apatite forms with Ca:P ratio 1.55. In both cases fluorine was detected, suggesting the mineral phase is fluorapatite.

CONCLUSION: STNA15-ELP promotes FAp formation in supersaturated fluoridated solutions. Ca:P ratios were altered by modifying how protein was added to mineralising solution. Our results suggest that immobilised protein, controls mineralisation, whereas random apatite formation occurs with free protein in solution, representing, indirectly, an effect of STNA15-ELP conformation on mineral formation. Thus controlling protein conformation can allow organised and aligned crystallites to form, opening the possibility for ELPs to be used as a tool in enamel remineralisation and regeneration.

DISCUSSION:

Janet Moradian-Oldak (Southern California): Can you just connect what you discussed in the previous talk with this talk, do you look at fluorapatite or hydroxyapatite?

Kseniya Shuturminska (QMU): I suppose I am trying to build a mechanism at the molecular level other than the scaffolds in the cells. We do grow fluorapatite, but this is because it is more resistant in the acidic environments. We prefer to grow some kind of structure other than the hydroxyapatite, which can then re-dissolve if we try and use it as a therapeutic.

Janet Moradian-Oldak (Southern California): Will you use Sherif's system to grow fluorapatite?

Kseniya Shuturminska (QMU): If we can understand how these proteins mineralise and we can control this process, then we can apply it to the membrane system. To have control over that means that we can use it therapeutically.

#21 ENAMEL MATRIX CONFIGURATION CHANGES DURING MINERAL TRANSPORT, NUCLEATION AND GROWTH

YAN, X, JIN, T, DANGARIA, SJ, ALLEN, M, LUAN, X, and DIEKWISCH, TGH*

Brodie Laboratory, University of Illinois at Chicago, Chicago, IL

E-mail: diekwisch@bcd.tamhsc.edu

Enamel formation is a continuous process that involves (i) mineral transport, (ii) crystal nucleation, and (iii) crystal elongation/maturation.

OBJECTIVES: To test whether the enamel protein matrix is involved in all three phases of enamel crystal growth.

METHODS: Recombinant amelogenin molecules were subjected to atomic force microscopy, fluorescent imaging of *in situ* aggregates, and labeling studies. Transmission electron micrographs of enamel matrix assemblies were analyzed for subunit dimensions and shape. Furthermore, key portions of the enamel organ were probed with antibodies against fragments of the amelogenin molecule.

RESULTS: Our data indicate that amelogenin proteins self-assembled into parallel rows of pyramidal elevations on freshly cut mica surfaces. In solution, enamel matrix proteins formed fluorescently labeled lines of self-assembled proteins. Analysis of transmission electron micrographs of developing mouse molar enamel revealed supramolecular assemblies within secretory vesicles and in the early enamel matrix. On these micrographs, the onset of initial enamel crystal precipitation was associated with a rapid conformational change in matrix configuration, both in 3 days postnatal mouse molars and in E16 +12 days organ cultured samples. His-labeled recombinant amelogenins revealed electron-dense U-shaped rings on the inner surface of assembled matrix subunits. Finally, amelogenin fragment Western blot mapping of select phases of the developing enamel organ linked the polyproline-rich and the C-terminal region of the amelogenin molecule with growing enamel crystal surface.

CONCLUSION: Specifically, these data link the N-terminal amelogenin fragment with the assembly of enamel matrix subunit compartments prior to enamel crystal nucleation and the C-terminal amelogenin fragment with the growing crystal surfaces. Together, these data establish the enamel matrix as a multifunctional protein assembly involved in all aspects of enamel formation, including ion transport, crystal nucleation, and crystal maturation.

DISCUSSION:

Derk Joester (Northwestern): Could clarify in your model whether the hexagonal units of the crystal makes them assemble in an orientated format so that you end up with a single crystal?

Tom Diekwisch (Dallas): So I think that the rods in amelogenesis self-assemble in an elongated fashion; there is something about amelogenin that drives the direction of self-assembly. Our model is based on studies where we've shown that these amelogenins have a phenomenal ability to group these crystals, make them adhere and condense them.

Derk Joester (Northwestern): At what point do you actually form the crystal and how is the crystal growth direction controlled? How does that evolve out of your model?

Tom Diekwisch (Dallas): The growth direction comes from deposition from the direction of the matrix deposition.

#22 TOWARDS A 4-D MODEL OF HUMAN DENTAL ENAMEL BIOMINERALISATION

AL-MOSAWI, M*, DAVIS, GR and AL-JAWAD, M

Institute of Dentistry, Barts and The London School of Medicine and Dentistry, London, E1 4NS, UK

Email: m.al-mosawi@qmul.ac.uk

OBJECTIVES: Precise timings and spatial progression of human enamel biomineralisation are still largely unknown due to scarcity of developing human enamel specimens available for investigation. However, this information is crucial for understanding how the hierarchical structure of enamel occurs. The aim was to characterise and compare the crystallography, mineral concentration (MC) and microstructure of human enamel at various maturation stages towards building a temporal and spatial model of human enamel biomineralisation.

METHODS: Four archaeological developing permanent upper central incisors and a type-matched mature contemporary tooth were compared. X-ray microtomography (XMT) with 15 μm^3 resolution at 90 kV was used to map MC distribution. Synchrotron X-ray diffraction (S-XRD) at the European Synchrotron Radiation Facility and at Diamond Light Source were used to quantify hydroxyapatite crystallites organisation (texture magnitude and orientation). Scanning electron microscopy (SEM) was used to assess and visualise the microstructure.

RESULTS: XMT revealed that overall as enamel matures the MC increases. In developing enamel, MC was highest near the enamel-dentine junction (EDJ) and the incisal tip, however, in developed enamel, MC was more homogeneous, showing that mineralisation starts at the incisal tip and the EDJ and progresses down the tooth incisal edge as a function of maturation. S-XRD revealed that two populations of crystallite orientations coexist with an angular separation of 30°-50°, and higher crystallite alignment (texture magnitude) in one population compared to the other. Crystallites were oriented approximately perpendicular to EDJ in all development stages, indicating initial crystallites preferred directions persist from early through to full maturation. SEM revealed that the intra-prism crystallites mineralise first followed by prism boundaries. Using SEM, it could be seen that the two populations observed with S-XRD are most likely due to prism decussation.

CONCLUSIONS: These results provide new insight into the fundamental understanding of natural growth and formation of human incisal enamel.

ACKNOWLEDGEMENTS: Mills, D, Montgomery, J, Beaumont, J, Wermeille, D, Bouchenoire, L, Bikondoa, O, Thompson, P and Brown, S.

DISCUSSION:

Jim Simmer (Michigan): When you are getting the two different orientations, did you consider that those could be due to rod and inter-rod enamel?

Mohammed Al-Mosawi (Barts and The London): Brookes' paper refuted this idea because there is a gradual change between the rod and inter-rod enamel, the tails and heads of the present rod. I also

believe that this is most likely due to prism decussation rather than heads/tails relationship. Here we see significant, not gradual, pronounced differences between the two different orientations.

Jim Simmer (Michigan): Is the thickness of these two peaks maintained throughout the thickness of the enamel, or is it more strong in some places than in others?

Mohammed Al-Mosawi (Barts and The London)*:* Well we found that it is less well pronounced in cuspal enamel, less pronounced near the surface of the enamel, more pronounced closer to the EDJ, but both are always present. The appearance shows as more pronounced peaks near the EDJ and not the enamel under the cusp.

PANEL DISCUSSION:

Janet Moradian-Oldak (Southern California): This is a question for Colin Robinson. We saw in your presentation some interesting images and their interpretation and there are some spheres there and some interpretation about assembly of amelogenin and the presence of mineral. What do you recommend to the next generation of students and new investigators in how to pursue this? What do you think can be done to clarify this area based on your experiences? What is the next step, because obviously we have some really good advanced technology imaging? We have cryo-techniques, we have FIB, we have correlated microscopy. What can we do next? We certainly cannot go back and zoom our images. We have to go a little bit farther. Is there anything you can recommend, or Tom, Jim or anybody?

Colin Robinson (Leeds): That's kind of a tall order! Do I advise the next generation of scientists in the room? No, but I'll simply rely on the approach we have taken, which is to say, look at the tissue which you are investigating and try to avoid any possibility of artefact. That's where we started. This is because many years ago, we looked at beautiful pictures of apatite crystals from Hershey Warshawsky, Antonio Nanci *et al*, and time and again, we described how they produced them: dehydrate, fix the proteins and embed in a hydrophobic plastic such that the dielectric constant changes. As a chemist, I said that's exactly what I would do to bring a crystal or a mineral out of solution! I said at the time that we've got to answer that question. It's not to say the crystals were not there in the images but were they there when you actually fixed the tissue? Are we looking at artefacts? This is not to say that artefactual material is not important, it is and it's very useful, but you need to consider whether or not it is an artefact. So from my own personal point of view, we tried to stay with the tissue and avoid artefacts if possible.

Janet Moradian-Oldak (Southern California): With new technology we definitely do have the potential to try to avoid those artefacts but we would also want to believe that not everything we publish and we talk about is an artefact. Just look at these things and interpret them more carefully. I invite the audience, if they have any additional comments about this, to participate.

Derk Joester (Northwestern): I have a comment for Colin. It is very easy in freeze-etched samples to create 10-50 nm nodular structures, no matter what you have in your sample. It's a freeze-etching artefact. So I would be very, very careful in interpreting freeze-etched samples at that level of resolution, with any sort of technique.

Colin Robinson (Leeds): You may be correct but you're going to have to produce more detail. Secondly, the point is, there were no crystals in the images, and a crystal is very obviously different from those chains of spheres that we saw. Also, the chains of spheres are the same size and the same orientation as the crystals, so there is a lot of weight behind saying that these are forerunners of crystals. If you look in mature enamel with the AFM, where all that has been done to the tissue was just a polish, you get the same kind of subunits. So the weight of evidence I think, suggests that whether or not that's an artefact at 15 nm in the freeze-etched images, it's the same size as the mineral subunits in mature enamel.

Wendy Shaw (Pacific Northwest National Laboratory): What was causing your transformation in the organised way from your circular structures and is it amorphous through all of the circular phases shown and only transforms to hydroxyapatite in the very last step?

Tom Diekwisch (Dallas): In fact the initial mineral precipitation causes a dramatic change in pH and that change in pH causes a change in configuration of the amelogenin to no longer be the one in terms of spacing, but to actually promote crystal growth. We are living in a fantastic time, the means of microscopy are ever expanding e.g. the recent advances in Focus Ion Beam Microscopy (FIB) and aberration correction microscopes; you can continuously reduce the number of artefacts. So as a new researcher in enamel (!), I can give you promise and hope that the microscope developers will always come up with new microscopes and advances in microscopy techniques and so fantastic new opportunities.

Wendy Shaw (Pacific Northwest National Laboratory): My question was aimed at Colin, not Tom.

Henry Margolis (Forsyth): I have a question for Dr Mukherjee. You mentioned in your presentation and your abstract about the robustness of your growing mineral and I was wondering how you assess that in such fine structures.

Kaushik Mukherjee (Southern California): We used FIB-TEM to study the interface between crystallographic dimensions of the synthetic layer and underlying native enamel. At the nanoscale level we saw a well-bound, seamless interface so that gave us an idea about the robustness of the interface at the nanoscale level. We also sonicated the samples well for 10-20 minutes, so what we observed is just the well-bound crystals, not loosely-bound crystals.

Henry Margolis (Forsyth): So you see a seamless interface; are you doing any biomechanical testing of any sort?

Kaushik Mukherjee (Southern California): We do complement that with mechanical testing using Vickers microhardness and nano-indentation tests as well.

Henry Margolis (Forsyth): But that gives you hardness, I'm thinking of the interface between the enamel mineral and the growth mineral.

Kaushik Mukherjee (Southern California): We did consider it but in materials science you have to have materials of much thicker dimensions to actually look into the interface bonding strength.

Henry Margolis (Forsyth): I might not call that robust, I might call it high resolution TEM, coherent or seamless.

Wendy Shaw (Pacific Northwest National Laboratory): Have you tried any enzymatic degradation to try to get rid of the proteins while you were growing the mineral?

Kaushik Mukherjee (Southern California): We have two experiments running side by side, one where we've used full-length amelogenin, LRAP and amelogenin-inspired peptides, and the second study where we are using MMP20 to see whether these enzymes can degrade the matrix and promote changes in crystal dimensions which would result in improved, enhanced or other differences in mechanical properties. We have observed some positive results.

Janet Moradian-Oldak (Southern California): We are going to present the MMP20 data at the 2017 IADR, San Francisco.

John Bartlett (Ohio): Colin mentioned that the enamel mineral is not crystalline during the secretory stage, that's not my impression, are you saying that?

Colin Robinson (Leeds): No. It may not be crystalline during that phase. What we don't know is, when it comes out of the ameloblast membrane, how soon is that transformed to apatite? What we saw with Warshawsky were crystals that went right up to the membrane. I'm saying that we don't know where that front is.

John Bartlett (Ohio): So you're not saying that enamel crystallisation doesn't wait until the maturation stage? I misunderstood, I'm sorry.

Kaushik Mukerhjee (Southern California): I have a question for Professor Robinson. In your slides you had nanospheres that are of 30 to 50 nm diameter and you also had smaller substructures. What do you attribute to these multiple sized substructures and nanospheres? For example, Professor Wendy Shaw has shown that it's actually a surface induced phenomenon, which results in multiple functional forms of amelogenin that are not near any nanosphere. What do you think would be the physiological significance *in vivo* of larger structures or smaller substructures in controlling crystal aspect ratio?

Colin Robinson (Leeds): We don't know what the nanosphere structures comprise, they may be amelogenin but they may be complexes of amelogenin and other proteins. I suspect they are. They must, however, contain mineral at their core if you look at the mature enamel, where we know the matrix has gone. I think there are just the two sizes. The smaller units we hadn't realised were there. When we looked at mature enamel crystals we find crystalline discontinuities in them which are because these units are fused. The interesting thing is, how do the small sub-units assemble with the mineral in the middle but then they are clustered into sizes which represent the final thickness of the crystal and stack in a length which tells you where the crystals are going to be? So the analogue is

laid down and then all you have to do is remove the stabilising protein for crystals to grow within that, but since there are a number of sites you get these "fusion lines" or "discontinuity lines".

Kaushik Mukerhjee (Southern California): So from what I understand from studies delving into protein-mineral interactions, the dynamics of the kinetics is crucial to understanding the whole process?

Colin Robinson (Leeds): Absolutely, you're then into guess work. A nice guess would be that it's the C-terminal end (of amelogenin) in the middle of the nanosphere because that is removed very quickly and if that's near the ameloblast membrane then in fact you would get that transformation occurring very quickly and the crystal following up behind as well. But the nature of those initial units and what is stabilising the mineral is a very crucial thing to find out.

Kaushik Mukerjee (Southern California): Thank you so much.

Tom Diekwisch (Dallas): A quick response to the same question. These very initial units, viewed with new methods of microscopy, in fact have a vortex-like appearance of a protein cylinder. Elia Beniash has done some beautiful studies of that. It turns out that the minerals accumulated in the centre of these tubes and the surrounding protein cues elongation. We have some data about that.

Alex Vieira (Pittsburgh): Dr Al-Mosawi, a very elegant presentation that I really appreciated. For someone like me who's thinking of a disease that affects the enamel, and affects a lot of people, like caries, one working argument that we have is that at the individual level, one might be more susceptible to acidic attack in a specific location. At the end of your presentation you mentioned something along those lines; that the organisation and the texture of the tissue is defined very early on. That makes me think that as genetic variation and epigenetic influences are defined quite early, how would you direct someone like me as to where to look?

Mohammed Al-Mosawi (Barts and The London): Yes the crystallites show two differently oriented populations. These are defined early on, but in the maturation stage, where the mineralisation is changing, it's changing quite significantly. You saw in the sample at the earliest developmental stage that you can see only a very small blue area indicating that is has high mineral density while at the end you will see the whole homogenous blue. Yes it's true that the orientation of the crystallites and the population of crystallites are defined early on but the main stage is the maturation stage and it's gradual as the tooth develops. Regarding your question that pathology can be predicted early on, you are right, but I don't know how to answer that to be honest.

Yong-Hee Chun (Texas): I have a question for Jim Simmer. It was nicely shown how wild-type initial enamel forms and I was wondering if you could summarise for us what we can learn from the knock-out mouse models?

Jim Simmer (Michigan): From the different knock out (KO) mouse models we looked at the secretory stage enamel; amelogenin, ameloblastin, enamelin and MMP20 with the focus high beam. In both the enamelin and the ameloblastin (KO) mice, no enamel ribbons form, period. The ameloblastin KO looks different to the enamelin KO in that there seems to be these accumulations of large amounts of what looks to be amelogenin on the dentine underneath the ameloblasts during this early stage and after but no mineral appears there. The cells also tend to go pathological and deteriorate. In the amelogenin KO, the initial ribbons form and then look completely wild type-like, so it seems that there's something a little bit wrong, but there's minerals that extend from the dentine surface to the ameloblasts that are orientated in that direction. So it seems that the in the amelogenin KO, you see the normal shape of the ribbons has already formed, so I think that casts a lot of doubt as to a role for amelogenin in shaping the ribbons as they form in the absence of amelogenin. The mineral also does not appear to be hydroxyapatite in the amelogenin null mouse, so that perhaps suggests that in the pathology that's created in its absence, that there is a problem controlling the mineral phase. That in turn adds extra credence to the idea that amelogenin could be helping regulate which mineral phase forms. In the wild-type enamel the things that struck us were, first of all, there was a very obvious continuation between the onset of the enamel ribbons and the dentine mineral. That suggested to us that the nucleation of enamel ribbons may be an actual mineral from the dentine, it's continuous with it and so it means that you don't necessarily have to propose a nucleator for the onset of enamel mineralisation. It looks like from the very beginning, and throughout, that the orientation of the ribbons is in the direction of the retrograde movement of the ameloblast membrane and so I think that that suggests that in the absence of the cell that there are problems with the idea that secreted proteins at a distance from the cell are orientating the ribbons. They're being oriented correctly as they elongate at the membrane right from the beginning to the end and so that's another thing that comes out of this is that looks like a very confident conclusion.

Janet Moradian-Oldak (Southern California): I just wanted to mention that when we talk about a case of pathological mineralisation, such as we see when a gene is removed, we have to be a little bit cautious about making statements or concluding about a chemical or physical process of controlling phase transformation, because so many things can go wrong with the cell and with the way that the cell brings things to the place. That's just my view. When there is pathology, things are just so messy, so we have to be a little bit careful about extrapolating to a function of that specific protein.

Henry Margolis (Forsyth): Janet started the session by saying that there are *in vitro* methods and *in vivo* methods, and they both have their limitations and one of the limitations in working with knock out mice is that sometimes the knock out affects the expression of another protein. It's my understanding, that in the ameloblastin knock out, amelogenin expression, as measured, is greatly reduced and it is non-existent in the enamelin knock out. So I think it's very difficult to then relate a property to amelogenin -that it doesn't do this or it doesn't do that -because there are limitations to that experiment. *In vitro* amelogenin shows special shape regulating properties and that's not relative to other proteins and so I think one has to be very careful in drawing conclusions.

Janet Moradian-Oldak (Southern California): I do encourage you all to continue this discussion, it is a great thing for everybody to know what are the limitations of the strategies that we use as well as what are the advantages.

Session 4: Evolution And Development

Moderator: Jim Simmer, University of Michigan, USA

KEYNOTE ADDRESS: FOSSILS, DEVELOPMENT, GENOMES AND FAMILY TREES: A FRAMEWORK FOR RECONSTRUCTING THE EARLY EVOLUTION OF ENAMEL AND DENTINE

PER AHLBERG

#23 KERATINS IN ENAMEL: A NEW CHAPTER IN AN OLD STORY

BENIASH, E*, YANG, X, DUVERGER, O, YAMAKOSHI, Y, MORASSO, M

#24 LOCALIZATION OF KERATIN 75 IN RAT AMELOBLASTS BY IMMUNOCHEMICAL TECHNIQUES

YANG, X*, DUVERGER, O, MORASSO, M and BENIASH, E

#25 FORMATION OF MAMMALIAN ENAMEL DEPENDS ON EVOLUTIONARILY CONSERVED SELF-ASSEMBLY MOTIF

WALD, T*, SPOUTIL, F, OSICKOVA, A, PROCHAZKOVA, M, BENADA, O, KASPAREK, P, BUMBA, L, KLEIN, O, SEDLACEK, R, SEBO, P, PROCHAZKA, J and OSICKA, R

#26 NEW TOOLS FOR QUANTIFICATION OF ENAMEL THICKNESS SPATIAL DISTRIBUTION

THIERY, G*, LAZZARI, V AND GUY, F

#28 CHARACTERIZATION OF PEPTIDES FROM ARCHEOLOGICAL TOOTH ENAMEL

STEWART, NA*, MONTGOMERY, J and GERLACH, RF

#29 PALEOPROTEOMIC ANALYSIS OF EARLY PLEISTOCENE FOSSIL REMAINS

CAPPELLINI, E*, FOTAKIS, A, RAKOWNIKOW JERSIE-CHRISTENSEN, R, BUKHSIANIDZE, M, COLLINS, MJ, OLSEN, JV, LORDKIPANIDZE, D and WILLERSLEV, E

KEYNOTE ADDRESS: FOSSILS, DEVELOPMENT, GENOMES AND FAMILY TREES: A FRAMEWORK FOR RECONSTRUCTING THE EARLY EVOLUTION OF ENAMEL AND DENTINE

PER AHLBERG

Department of Organismal Biology, Uppsala University

Email: Per.Ahlberg@ebc.uu.se

The fossil record of vertebrates yields a wealth of information about the early evolution of biomineralized tissues. Histological preservation is often near perfect, even in specimens that are close to 500 million years old, allowing the cellular and fibrous architectures, as well as the crystallite organization, to be investigated in detail. Crucially, fossils illuminate parts of the vertebrate family tree that are not represented at all by living vertebrates, such as the jawed vertebrate stem group (the common ancestral lineage leading to modern sharks, bony fish, and land vertebrates) where mineralized tissues including bone and dentine first evolved. Histological and morphological data from fossils can be integrated with genomic and developmental data from extant vertebrates by mapping them all onto the same family tree; this allows surprisingly detailed inferences to be drawn, not only about the evolution of the biomineralized structures themselves, but also about the evolution of their development and patterning.

Although we tend to think of teeth as structures specific to the mouth, the fossil record shows unambiguously that tooth-like structures (odontodes) were originally distributed across the whole body surface and probably originated there rather than in the mouth. Among extant vertebrates, only chondrichthyans (sharks and rays) and a few primitive bony fish such as gar (*Lepisosteus*) bichir (*Polypterus*) and coelacanth (*Latimeria*) still have odontodes in the skin. All teeth and external odontodes of living vertebrates develop at the contact surface of an epithelial fold and underlying mesenchyme; the morphology and histology of odontodes in early fossil vertebrates strongly suggest that they formed in the same way. However, while the mesenchyme always produces dentine, the role of the epithelium appears much more variable, as shown by the variable presence of different hypermineralized surface tissues.

In land vertebrates and lobe-finned fishes such as *Latimeria*, teeth and (if present) skin odontodes are covered with enamel and Enamel Matrix Protein genes are present in the genome. In teleosts (modern ray-finned fishes such as zebrafish) enamel and EMP genes are absent but the teeth are tipped with acrodin, a hypermineralized dentine. In the primitive ray-fins *Lepisosteus* and *Polypterus*, the teeth carry an enamel collar below the acrodin tip and the scales are covered in a strange multi-layered enamel known as ganoine. Two out of three EMP genes are present. Fossil evidence from the 425 million year old bony fishes *Lophosteus*, *Andreolepis* and *Psarolepis* indicates that enamel originated on the odontodes of the scales, whereas the teeth originally consisted of naked dentine. Sharks and rays have teeth and skin odontodes covered with enameloid, a superficially enamel-like tissue that is built on a collagen-rich matrix apparently secreted by the odontoblasts before the beginning of dentine formation. EMP genes are probably absent. True enameloid is unique to chondrichthyans and not homologous with enamel. However, during enameloid development the epithelial cells differentiate into an ameloblast-like morphology and probably deposit the mineral component of the tissue. Many early vertebrates have hypermineralized surface dentine, loosely described as "enameloid" but differing in structure from that of chondrichthyans. It appears that the odontode bud

epithelium always had the latent capacity to deposit mineral, but that this was deployed in different ways in different vertebrate groups.

DISCUSSION:

Jim Simmer (Michigan): First there are odontodes without enamel and then odontodes with enamel on the scales only. At the same time you have odontodes in the mouth without enamel. Where you have odontodes with enamel in the mouth, what are your thoughts? Did the entire odontodes that made enamel have to come into the mouth from the skin, or did the odontodes in the mouth start expressing enamel genes from the skin that caused that transition?

Per Ahlberg (Uppsala): The latter, I'm quite sure. Let's look at the actual organisation of the teeth and method of deployment, and think about the tooth form in *Psarolepis* which had enamel on the scales' surface but no enamel on the teeth. In every other respect, besides the absence of enamel, its teeth are perfectly conventional-looking lobe finned fish teeth, their morphology, their deployment, whatever. We have an odontode set in the mouth that is historically stable. Of course they undergo gradual evolution, but they have the ability to make the tissue that jumps onto this odontode set. So it is very much a matter of conferring a new set of abilities to the ameloblasts that are forming the teeth in the mouth. In *Psarolepis*, the ameloblasts that are forming the skin odontode are taking part in enamel production and those in the mouth are not. In slightly more derived lobe finned fishes, the odontodes in the mouth are also making enamel but it's because they have taken up a new ability, not because of an actual physical replacement of one set of odontodes by another.

Jim Simmer (Michigan): What are the possibilities that there are unfound fossils from an earlier era that have enamel on both scales and teeth?

Per Ahlberg (Uppsala): Quite possible, we can only look at what we have. At the moment our whole understanding of really early jawed vertebrates and early bony fish evolution is being shaken up by a series of discoveries of really magnificent material from the Silurian and early Devonian of China. We had a paper out in Science last week [M Zhu *et al*., (2016) Science 354:334-6] and I'm working in close collaboration with Chinese colleagues describing a form called *Qilinyu,* which stands at the interface of placoderms and bony fish. Suffice to say that yes, there may yet be major surprises. Of course, we have to work with what we have for the present. There's a lot of new data coming. We have to be open to that. And we have the application of new techniques like synchrotron tomography being applied to existing material, which can also hopefully get us further than where we are now. This is a very active field of research, so we will see what comes.

#23 KERATINS IN ENAMEL: A NEW CHAPTER IN AN OLD STORY

BENIASH, E[1 2]*, YANG, X[1], DUVERGER, O[3], YAMAKOSHI, Y[4], MORASSO, M[3]

[1]Department of Oral Biology, University of Pittsburgh, Pittsburgh, PA, USA; [2]Department of Bioengineering, University of Pittsburgh, PA, USA; [3]Laboratory of Skin Biology, NIH/NIAMS, Bethesda, MD; [4]Department of Biochemistry and Molecular Biology School of Dental Medicine, Tsurumi University, Yokohama, Japan

Email: ebeniash@pitt.edu

OVERVIEW: During the maturation stage of amelogenesis, the majority of matrix proteins are degraded and replaced by thickening enamel crystals. However, a tiny organic fraction, containing an insoluble proteinaceous material, remains in the mature tissue. The protein composition of the mature enamel matrix (EM) remains unknown to this day due to its extreme insolubility. Earlier studies demonstrated that amino acid composition of mature EM is similar to keratins. Later immunochemical studies suggested a presence of keratin-like components in mature EM. Our recent findings of Keratin 75 (KRT75) in ameloblasts and enamel, and the link between mutations in this gene and higher susceptibility to caries, raise questions about its role in this tissue. Since keratins are cytosolic proteins, lacking signal peptide, they cannot be secreted via conventional secretory pathways. Solving this mystery of trafficking and secretion of KRT75 can significantly advance our understanding of amelogenesis and will potentially address a fundamental cell biology question, namely, how a cytosolic protein lacking a signal peptide enters a secretory pathway?

EVIDENCE: (1) We observed KRT75 secretory vesicles in cell bodies and Tomes' processes of secretory ameloblasts by immunohistochemistry. We also found KRT75 signal in enamel matrix by immunochemistry and Western blot. (2) Several literature reports demonstrate specific interactions between enamel matrix proteins and keratins. Furthermore, mutations in a keratin-binding domain of amelogenin, causing amelogenesis imperfecta, disrupt the binding. (3) A number keratins were found extracellularly in other tissues. (4) Recently, several unconventional secretory mechanisms for cytosolic proteins, i.e. secretory autophagy and lysosomal exocytosis, were identified.

CONCLUSION: The evidence listed above lead us to a hypothesis that KRT75, and potentially other keratins, enter the secretory pathway via an unconventional mechanism and that interactions between keratins and enamel matrix proteins, such as amelogenin and ameloblastin, are critical for amelogenesis and enamel structural and mechanical integrity.

DISCUSSION:

Ariane Berdal (Paris): There is an old question in the enamel field dating back to Warshawsky and other electron microscopists. The question was: could the whole Tomes' process be an exosome? That is, a part of a cell that would be externalised. Would the secretion of matrix into the enamel follow a classical secretory pathway? Am I clear?

Elia Beniash (Pittsburgh): I know these stories and essentially the Tomes' process is so actively secreting that some parts of it are actually dislodged into the enamel. There is electron microscopy evidence of this.

Ariane Berdal (Paris): So that would be the pathway?

Elia Beniash (Pittsburgh): I don't think so because the other proteins are definitely secreted and not shed and keratin 75 co-localises with them.

Ariane Berdal (Paris): So do you think your keratins would go through the classical secretory pathway?

Elia Beniash (Pittsburgh): No, not the classical secretory pathway as it doesn't have signalling peptides. We don't know the pathway.

Janet Moradian-Oldak (Southern California): Have you done any biochemical studies and is there any evidence of the protein co-migrating on SDS gels with amelogenin and/or other proteins?

Elia Beniash (Pittsburgh): Yamakoshi did Westerns on porcine unerupted teeth and he sees this in both secretory and maturation stages, and what's interesting is the amount seems to be the same, so it doesn't degrade.

#24 LOCALIZATION OF KERATIN 75 IN RAT AMELOBLASTS BY IMMUNOCHEMICAL TECHNIQUES

YANG, X*[1], DUVERGER, O[2], MORASSO, M[2] and BENIASH, E[1 3 4]

[1]Department of Oral Biology, University of Pittsburgh School of Dental Medicine, Pittsburgh, PA, USA; [2]Laboratory of Skin Biology, NIAMS, Bethesda, MD, USA; [3]Department of Bioengineering, Swanson School of Engineering, University of Pittsburgh, Pittsburgh, PA, USA; [4]Center for Craniofacial Regeneration, McGowan Institute for Regenerative Medicine, Pittsburgh, PA, USA

Email: xuy14@pitt.edu

OBJECTIVES: Keratin 75 (KRT75) is a type II keratin that is expressed in the companion layer of hair follicle and plays critical roles in supporting the hair shaft. We recently discovered KRT75 in enamel and demonstrated that a single point mutation KRT75 p.A161T, which causes pseudofolliculitis barbae, increases susceptibility to caries[1]. The aim of this study is to identify stages of enamel formation at which KRT75 is expressed and its localization.

METHODS: Mandibles from 4 week-old rats were fixed in 4% paraformaldehyde, demineralized in EDTA, embedded in paraffin, LR White and Epon, sectioned and subjected to immuno-fluorescence (IF) and immunogold TEM (IG TEM).

RESULTS: IF studies revealed that KRT75 is expressed primarily in secretory stage ameloblasts. We observed fluorescent granules in the distal regions of ameloblasts and diffuse fluorescence in the cytoplasm and Tomes' process. IG TEM studies confirmed the existence of KRT75 in large multivesicular bodies (400 nm - 2000 nm) in the distal portion of ameloblasts. KTR75 was also found in smaller granules (around 100 nm) in the cytoplasm and Tomes' processes of ameloblasts and adjacent stratum intermedium. Importantly, no association of IG signal with intermediate filaments was detected. Some IG signal was also observed in the enamel matrix at the secretory stage of amelogenesis, although no preferred localization to the periphery of rod in rat enamel was found under TEM. No signal was identified in dental tissue.

CONCLUSION: KRT75 is expressed primarily by secretory stage ameloblasts. It was found in vesicles of secretary ameloblasts and enamel matrix. Our results suggest that KRT75 is secreted into the matrix, confirming earlier observations. These findings are intriguing since generally the keratins have been reported as cytosolic structural proteins assembling into intermediate filaments.

[1]Duverger, O et al. Hair keratin mutations in tooth enamel increase dental decay risk. *The Journal of Clinical Investigation* 124, 5219-5224, doi: 10.1172/JCI78272 (2014).

No questions

#25 FORMATION OF MAMMALIAN ENAMEL DEPENDS ON EVOLUTIONARILY CONSERVED SELF-ASSEMBLY MOTIF

WALD, T[1]*, SPOUTIL, F[2], OSICKOVA, A[1], PROCHAZKOVA, M[2], BENADA, O[1], KASPAREK, P[2], BUMBA, L[1], KLEIN, O[3], SEDLACEK, R[2], SEBO, P[1], PROCHAZKA, J[2] and OSICKA, R[1]

[1]Institute of Microbiology of the CAS, v. v. i., Videnska 1083, 142 20 Prague 4, Czech Republic; [2]Institute of Molecular Genetics of the CAS, v. v. i., Videnska 1083, 142 20 Prague 4, Czech Republic; [3]Institute of Human Genetics, University of California, San Francisco, CA 94143-0442

Email: tomas.wald@gmail.com

OBJECTIVE: One of the central processes during vertebrate evolution is the occurrence of mineralized tissues, which have enabled various adaptive phenotypes such as endoskeleton for locomotion (bone), body armor for protection, and teeth (enamel, dentin) for predation. While formation of bone and dentin depends on self-assembly of type I collagen, the most abundant mammalian matrix protein, via consecutive Gly-X-Y motif, the mechanism of self-assembly of enamel matrix proteins (EMPs) and its role in formation of an organized layer of mineral crystallites remains poorly understood.

METHODS: Recombinant protein expression and purification, size exclusion chromatography (SEC), surface plasmon resonance (SPR), transmission electron microscopy (TEM), transgenic mouse generation by TALEN technology, scanning electron microscopy, μCT, histology, immunohistology, qPCR, western blot.

RESULTS: By using SEC, TEM and SPR followed by sequence alignment we report here identification of a novel evolutionarily conserved self-assembly motif, common to the major EMPs, ameloblastin and amelogenin (two evolutionarily related proteins), which is essential for polymerization of both proteins into higher-order structures. To confirm the indispensability of the self-assembly motif for enamel formation, we generated mice with targeted mutations in ameloblastin. Using high-resolution imaging, we demonstrated that the self-assembling capacity of ameloblastin determines the organization of enamel organic matrix and accounts for proper organization of hydroxyapatite crystallites into compact bundles that make up the structure and ensure the mechanical resistance of enamel.

CONCLUSION: This is the first *in vivo* evidence that the EMP self-assembly into supramolecular structures is essential for the development of highly structured enamel in vertebrates.

No questions

#26 NEW TOOLS FOR QUANTIFICATION OF ENAMEL THICKNESS SPATIAL DISTRIBUTION

THIERY, G*, LAZZARI, V AND GUY, F

iPHEP UMR CNRS 7262 INEE, 6 rue M. Brunet, Bat B35, TSA 51106, Universite de Poitiers, 86073 Poitiers Cedex 9, France

Email: ghislain.thiery@ntymail.com

OBJECTIVES: Because thick enamel may prevent tooth failure, enamel production is highly susceptible to natural selection. Consequently, it has been suggested that primates consuming hard or abrasive food on a regular basis have thick-enameled molars in comparison to primates consuming soft food. Furthermore, variation in the spatial distribution of the enamel thickness (ETSD) is expected to reflect tooth adaptation to durophagy. For instance, thick-enameled cusp tips of *Paranthropus* or thin-enameled cusp tips that result in a lower relief in *Gigantopithecus*, are both interpreted as a protection against tooth fracture. Still a proper methodology to quantify ETSD remains to be developed.

METHODS: Here we introduce new dental variables for measuring ETSD, as well as enamel thickness 3D variation. Using an X-ray microtomography scanner, we digitized unworn to slightly worn upper second molars from 20 genera of anthropoid primates, corresponding to a wide range of food cracking abilities. From 3D virtual reconstructions of the enamel cap, we computed the following variables: (1) 3D average enamel thickness; (2) enamel pachymetric profiles; (3) enamel thickness per increment of occlusal orientation.

RESULTS: Using variables (1) and (2), we tested the hypothesis that cusp and crest tips of primates adapted to hard food cracking are characterized by relatively thick apical enamel, as seen in *Paranthropus*. In addition, we used variable (3) to test whether jaw movements during mastication have some influence in ETSD. Our results show that there might be more than one modality of hard food cracking. They also point toward an influence of average enamel thickness over ETSD.

CONCLUSIONS: The new variables detailed in this work separated different kinds of adaptation to hard food cracking. This might change our way of assessing dental adaptation to hard food consumption in paleoanthropology. Overall ETSD offers multiple implementations for deciphering dental evolution of extant and extinct primates.

ACKNOWLEDGEMENT: This work was funded by the Centre National de la Recherche Scientifique (CNRS INEE), the Ministere de l'Education Nationale, de l'Enseignement Superieur et de la Recherche, the Universite de Poitiers and the Region Poitou-Charentes (Conventions #07/RPC-R-100 & #12/RPC-013) and the International Primatological Society.

DISCUSSION:

Jim Simmer (Michigan): When you are looking at the differences in (cusp) slope depending upon whether the diet was higher or lower in hardness, could you explain why it's advantageous to have a higher or lower slope for cracking harder foods?

Ghislain Thiery (Poitiers): The slope is a consequence of the difference between the maximal and the minimal enamel thickness. For example, when the minimal and maximal enamel thicknesses are similar, this means that the enamel thickness is more evenly distributed. Greater slopes mean you have really thick enamel at the cusps with really thin enamel over the tooth surface. In terms of the mechanics, it doesn't propose that thicker enamel on the apex of the cusps acts as protection for the cusps of the teeth, allowing them to be functional, even with wear. Where there is thicker enamel on the tip of cusps even when the individual is older, it could still crack.

Jim Simmer (Michigan): When you have a greater slope does that put any more sheer stress on the tooth? Would you worry about the tooth being able to fracture with a higher slope or a lower slope? Do you have any considerations as to whether or not the tooth would break in these different shapes?

Ghislain Thiery (Poitiers): I did not test this. I will have to test it using biomechanical approaches and/or finite elemental analysis.

#28 CHARACTERIZATION OF PEPTIDES FROM ARCHEOLOGICAL TOOTH ENAMEL

STEWART, NA[1]*, MONTGOMERY, J[2] and GERLACH, RF[3]

[1]School of Pharmacy and Biomolecular Sciences, University of Brighton, Brighton, BN2 4GJ, UK; [2]Department of Archaeology, Durham University, Durham, DH1 3LE, UK; [3]Department of Morphology, Physiology and Basic Pathology, School of Dentistry of Ribeirão Preto, University of São Paulo, Ribeirão Preto, SP, 14040-904, Brazil

Email: n.stewart@brighton.ac.uk

OBJECTIVES: Dental enamel is unique in being the hardest, densest and most mineralized tissue in vertebrates. The crystalline structure of enamel is a result of a maturation process involving enamel specific proteins. During maturation these proteins are processed by proteases with only peptides constituting <1 % proteinaceous material remaining in mature enamel. A major protein involved in tooth development, amelogenin, is expressed as dimorphic isoforms from both the X and Y chromosomes and therefore the sequencing of these gender dimorphic peptide regions provides a promising means of determining sex in the human, especially when no other biomaterial is available. The main purpose of this study was to investigate the possibility to recover and identify Y-chromosome-specific enamel peptides from archaeological teeth samples.

METHODS: A method employing nanoflow liquid chromatography (nLC) electrospray ionization tandem mass spectrometry (MS/MS) was used to analyze peptides released through an acid etch of the enamel from single teeth from archaeological sites dating back to 5,500 BP.

RESULTS: Peptides from the major enamel structural proteins were identified including: amelogenin isoforms, ameloblastin, and enamelin. Modified peptides were also identified, including phosphorylation and deamidation. Furthermore, Y-chromosome-specific amelogenin peptides were also detected in mature enamel.

CONCLUSION: Peptides can be recovered and identified from the enamel of single teeth from archaeological skeletons. The identification of sex specific peptides with this approach allows for potential sexing applications in forensic and archaeological studies when high quality DNA is unavailable and will be particularly useful for sexing juvenile skeletons that have yet to develop reliable osteological indicators of sex.

DISCUSSION:

Jim Simmer (Michigan): Did you see any Keratin 75 in there?

Nicholas Stewart (Brighton): I did not. I think if you process the samples in a clean manner you shouldn't see much keratin. Keratin is typically a contaminant with many proteomic samples, you could run nothing and still see keratin, so you have to be careful. This is not to say that it's not there though, it depends on the peptides, on their ionisation and other properties. So it may be there, but not in abundance.

Mike Hubbard (Melbourne): What you referred to as "crap", probably contains a lot of material that other people are interested in in terms of adsorbed surface proteins. What did you do to get rid of surface proteins including pellicle?

Nicholas Stewart (Brighton): It was passed through a solvent extraction to get rid of most of the protein, but there were some issues with high pressure in the system. This didn't seem to be due to high protein load but rather particulate matter that would clog the system.

Mike Hubbard (Melbourne): Did you polish the tooth?

Nicholas Stewart (Brighton): Yes, we did.

Raquel Gerlach (Sao Paulo): When looking at peptides in old or ancient teeth, I would really recommend carrying out two etches. We didn't use the first etch, we used the second one. Both were very shallow etches, 3 µm in depth, enough to get rid of the outer contaminants. From the very first surface etch you will get keratins or all kinds of things derived from saliva. The second etch is 3 to 6 µm from the surface. This can be calculated by measuring phosphate or calcium concentrations in the etch, so you know the depth from which your sample comes. In the second etch you almost only get enamel proteins, Nicholas has seen this many times because people don't have to destroy teeth from museums.

Elia Beniash (Pittsburgh): I'm really interested in the discussion about the sample preparation. In our study, we actually removed all of the enamel and collected the insoluble fraction, the "tuft" protein, extracted with SDS and digested proteolytically for three days. We still see a lot of blood proteins, dentine proteins, basal lamina proteins and keratins. So are these proteins there in the enamel, or are they contaminants?

Nicholas Stewart (Brighton): This is the method we used, perhaps it's not extracting those peptides as protein.

#29 PALEOPROTEOMIC ANALYSIS OF EARLY PLEISTOCENE FOSSIL REMAINS

CAPPELLINI, E[1]*, FOTAKIS, A[1], RAKOWNIKOW JERSIE-CHRISTENSEN, R[2], BUKHSIANIDZE, M[3], COLLINS[4], MJ, OLSEN, JV[2], LORDKIPANIDZE, D[3] and WILLERSLEV, E[1]

[1]Centre for GeoGenetics, Natural History Museum of Denmark, University of Copenhagen, Øster Voldgade 5–7, 1350 Copenhagen K, Denmark; [2]Novo Nordisk Foundation Center for Protein Research, Faculty of Health Sciences, University of Copenhagen, Blegdamsvej 3b, 2200 Copenhagen, Denmark. [3]Georgian National Museum, 3 Purtseladze Street, 0105 Tbilisi, Georgia; [4]BioArCh, University of York, York YO10 5DD, UK.

Email address: ecappellini@snm.ku.dk

OBJECTIVES: Ancient DNA sequencing liberated geneticists from accessing present-day genomic information only. Recovering ancient genetic information has enabled reconstruction of molecular phylogenies for extinct species and major breakthroughs in phylogeography and population genetics. Nevertheless, despite technological and methodological improvements in recovering and sequencing small amounts of damaged DNA, the oldest genomes reconstructed so far date to less than 1Ma, even in high latitudes.

METHODS: Sequencing of ancient protein residues using mass spectrometry represents an alternative solution to extend biomolecular investigation further back in time. Ancient protein residues persist in fossil samples much longer than DNA.

RESULTS: We will present preliminary data demonstrating feasibility of retrieving ancient proteomes, extracted from fossil remains dated to approximately 2 million years ago (Mya) and originating from temperate geographic areas.

CONCLUSION: These results, obtained from dental enamel, enable molecular-based phylogenetic reconstruction of early Pleistocene remains.

<u>DISCUSSION:</u>

Elia Beniash (Pittsburgh): How much AMELY protein is present in enamel during amelogenesis? I had understood that it's not a functional protein and essentially not produced in any significant amount.

Enrico Cappellini (Copenhagen): I'm sure there is someone more competent than me to answer this question, but what I've found in the literature is that 10% of amelogenin production is the Y form, so the ratio (of AMELX to AMELY) is not equal.

Jim Simmer (Michigan): Salido [EC Salido *et al.*, (1992) Am J Hum Gen 50:303-16] said it was 10% of the total amelogenin transcripts and it is still considered to be functional, but it simply can't by itself cover the function(s) of amelogenin if AMELX is missing.

Per Ahlberg (Uppsala): Are you going to be looking at the hominin materials? You're talking about mammalian material in general but Dmanisi is one of the most important non-African early hominin sites in the world.

Enrico Cappellini (Copenhagen): Dmanisi is the site where the evidence of the most ancient traces of humans is, outside of Africa. At the moment we only worked on large mammals from there simply because we wanted to check the methods on less precious material first. The previous speaker clearly highlighted the kind of concerns we face. We found out quite surreptitiously that enamel was a new source of ancient proteins. In terms of application of our research to hominins, potentially yes, but we want to consolidate things first.

<u>PANEL DISCUSSION:</u>

Colin Robinson (Leeds): Can I ask something about the keratins and a little bit more? There's an awful lot of insoluble tuft protein near the dentine which contains keratin, but it seems to be very intimately associated with the prism boundaries. There's a lot of protein there and it attenuates through the enamel. It's also very highly cross-linked and I'm wondering if you think this material has anything to do with the formation of prisms, and given it's cross-linked, is it susceptible to any of the enamel proteases (MMP20 or KLK4)? Secondly, are we are looking at a recapitulation of ontogeny? Does enamel start by forming enameloid, "enamelish", or any of the other weird things, but then it forms enamel after that?

Per Ahlberg (Uppsala): The very short answer would be, I'm not certain! This is really something that could do with a more in-depth investigation. The thing that seems very clear to me when looking at odontoid evolution generally is that the thing that is truly and obviously conserved is the interaction or the presence of two groups of cells: the epithelium and the underlying mesenchyme. There are obviously components of the interactions between them that are very highly conserved. There are others that, I suspect, are switched on and off. There is an evolutionary story to delve into here that would start to give answers to that question. I'm really intrigued by this business with the keratin, partly because, as I mentioned briefly in the talk, if you go to the extant jawless vertebrates, they have no biomineralised tissues, however what they do have, lampreys in particular, are rather nasty hooks which are said to be keratinized, but I have not looked into what the actual basis for that is. You know there are many keratins. I don't know how old the keratin 75 gene (*KRT75*) is, phylogenetically. This is an obvious one to look at. Do we actually have a *KRT75* gene in ray-finned fish for example? Do we have it in chondrichthyans and so on? This is the sort of information that might begin to allow us to build a framework for understanding the early evolution of these interactions. It's the best answer I can give at the moment.

Elia Beniash (Pittsburgh): I kind of already forgot Dr Robinson's question, but I'll try to answer it as best I can. Is it (keratin) involved in the formation of rods? Based on analysis of the enamel from people carrying a single amino acid mutation in *KRT75*, it's clear that the enamel prism (rod) pattern is compromised, therefore it is quite likely that KRT75 plays a part in the shaping of the prisms (rods). In terms of the highly cross-linked nature of this protein and the amount present during enamel formation, Yasuo Yamakoshi actually did the Western blot analysis on this, and what he found was that, based on just looking at the staining intensity, the amount of keratin 75 didn't obviously change from secretory to maturation stages. He hadn't seen anything like this in enamel before because usually the proteins start to degrade and you can see the degradation pattern very clearly as amelogenesis progresses.

Jim Simmer (Michigan): I would like to add one comment about part of Dr Robinson's question and that is the question of whether enamel starts off as enameloid but then goes on as enamel. It seems quite clear that the onset of enamel mineralisation is actually enamel formation with the characteristic ribbons starting right on the dentine and continuing in the same manner all the way through the secretory stage, so I don't think that the evidence supports an idea of enameloid preceding enamel during regular tooth production.

Colin Robinson (Leeds): I'm saying that there is some kind of memory there. It doesn't produce enameloid.

Jim Simmer (Michigan): The typical enamel proteins are expressed right from the beginning of enamel secretion and the process looks the same.

Jason Wan (Bethesda): What are the efforts in biomimetics? Part of what we heard from the previous session was to look at amelogenins, or maybe statherins, to see if we can use the information to recapitulate the structure of enamel. I was wondering what the potential might be to look at maybe some of the enameloid or homologous proteins that are found in some of these extant species and whether we might be able to explore some of those avenues in biomimetic strategies.

Per Ahlberg (Uppsala): This is a slightly tricky one. As you will have gathered from my talk, a lot of what we do within my group hangs on integrating very different data sources within a phylogenetic framework. Now with the fossil material, of course, we get no direct preservation of proteinaceous material. If you're looking at archaeological-aged materials, as we've just seen, you can have actual peptide sequences in there. Nobody's actually looked at (much older) material like from the Silurian, but frankly, I think finding anything would be very lucky. What we have are more or less well-preserved, or perhaps somewhat imperfect, crystallite architectures. To get at that, we need to go at it from the angle of the extant organisms. We need to look at the related protein families that we have amongst extant animals, and you are looking at the usual range of manipulative experiments with extant models to see how these different proteins might relate to biomineralisation, and then see if we can mesh that in with architectures that we observe in the fossil record. It's not an impossible avenue to pursue, but it's not a trivial thing to try to integrate.

Raquel Gerlach (Sao Paulo): Just a very short point. When you search proteomics databases the spectra for enamel protein peptides are not there, whereas keratin and albumin, for example, are the most highly represented proteins found in the databases. This is one problem that maybe the enamel field can help to solve. It would be very nice for the next years, if we want to pursue this, in for example, fossils and the like, that we create a record for future reference. If we simply search the databases now in the usual way that proteomics people do, we won't get the right data. It's important that people realise this before they start or they'll have bad results because we don't have data on enamel proteins in the databases. Samples from many other tissues are there but the enamel proteins are absent.

Mike Hubbard (Melbourne): Being a bit older than Raquel, I see it the other way round. Before all the fancy proteomics stuff came along we always had to re-sequence manually, so when you have beautiful spectra you can read them manually and then subtract all the other stuff that is everywhere. I think manual reading of the spectra would still move you forward but what we should do is get Raquel's data and others' data and put them all in the database so that there's an enamel spectrum database that everybody can share.

Tom Diekwisch (Dallas): I'm intrigued by your work on keratins. It reminds me of all these findings of keratins in claws, for example, or in baleen [whales]. Do you think that keratin is perhaps an evolutionary conserved template for mineral nucleation or crystallisation? I also find it interesting that you identified a specific keratin. When we did stains we found that pan-keratin antibodies label samples all the time, but not all keratins in fact would contribute to the staining in the enamel. Do you want to comment?

Elia Beniash (Pittsburgh): The keratin that we are talking about is keratin 75. Just the number tells you that there are quite a few of them (keratins) and there are two groups: basic and acidic, and they form hetero-dimers and assemble. Keratin 75 is very specific to a couple of cell layers in the hair follicle that actually "hug" the out-coming hair, so they probably have very specific functions. The thing is that, when you look at the sequences of these proteins, you cannot guess which keratin you have because their similarity is very, very high, even in the spectrum when you get it. In the program I use, there are asterisks at every keratin, which tell you that the sequences that you have obtained can belong to x number of proteins, but there is one sequence in particular that identified, this specific protein. It's a really tricky business especially considering obvious contaminations. Keratin 1 for example, could come from your fingers or elsewhere, so there are a lot of problems. Regarding the templating, it's possible, but I think there will be other nucleators. It's a very good mechanical template, but in terms of the nucleating properties of keratins, I cannot say anything.

Per Ahlberg (Uppsala): To me this is one of those problems that becomes really interesting from a phylogenetic perspective where we have a very clear picture, with conservation of keratin 75 shown rather widely across teeth if you look at a mammalian lineage and way beyond that. Teeth are much older phylogenetically than hair follicles. If keratin 75 has a specific role in mammalian hair follicles that is in all likelihood co-opted from the role in the teeth, not the other way around. This is the sort of thing that becomes relatively straightforward to answer in a phylogenetic framework and of course it's quite interesting also from a functional perspective.

Jim Simmer (Michigan): I personally find the keratin story intriguing, but I for one think that there's still huge obstacles here for a protein that doesn't have a signal peptide but is being secreted and yet is supposed to be literally the only protein left in maturation stage (enamel). Yet even after a thousand years you can (still) find amelogenin and ameloblasts and things. So I would just like to voice my concern that there's still a lot of work left to be done before we can accept that idea that keratin 75 is actually in the enamel matrix.

Enrico Cappelini (Copenhagen): Is it demonstrated that keratin 75 is present in mature enamel? Yes? So it could be retrieved and sequenced?

Elia Beniash (Pittsburgh): Our last run on the mass spec. showed a couple of sequences present in keratin 75 but the problem is that they were shared with keratins 6A or 6C, and therefore it cannot be positively identified as being there. As to the question of contamination, these keratins are hair follicle keratins, it's not like something that would fall off your gingiva or from your fingers, so it's

harder to imagine that these particular keratins, that never get to the skin surface, will be contaminants. Does that answer your question?

Enrico Cappelini (Copenhagen): Yes it does.

Janet Moradian-Oldak (Southern California): I have a question for Tomas Wald. I told you yesterday that I really like your animal model because it's a specific point mutation and you can pinpoint some function. But I was wondering if you had considered other mechanisms rather than self-assembly? You mention the effects on higher order organised structures, but there are other things that can go wrong because of this mutation, and one of them is that the protein is not appropriately cleaved, so those N-terminal portions that need to be around the prisms are not formed properly. Have you noticed if the proteolytic processing is disturbed, so the functional fragments are not formed correctly?

Tomas Wald (Prague): Thank you for your question. I think the proteolytic cleavage is something that we should like to be done, definitely, but we haven't checked that so far. I presented a Western blot of ameloblastin isolated from the incisors. We haven't observed any cleavages: not in the mutant mouse nor in the wild-type mouse, however definitely this is something which should be done, perhaps at the recombinant level, or with (*in vivo*) isolates. Regarding your second question for sure there might be effects that we did not consider, like for example, the interactions between ameloblastin and amelogenin, also their cleavage products.

Janet Moradian-Oldak (Southern California): I would like to get back to Jason's question about recapitulating some of these (non-enamel, "enamelish") tissues. In my opinion the main point here is the functionality. If the tissue that you want to recapitulate is functional in terms of the combination of hardness and modulus for example, or has the resilience that enamel has, I would say, why not? Maybe we can recapitulate "enamelish" as long as it really does function for repairing the tooth or if it is something that could be useful clinically.

Elia Beniash (Pittsburgh): So you hit a point because for many of the bio-inspired and biomimetic materials you can get nice features but you don't have designed parameters. For example, even when you talk about in this condition, the hardness or modulus is twice as high as in this (other condition) for a biomimetic, it can still be orders of magnitude lower than enamel, so how useful it is? Before we start to actually build our materials we should consider, not just that we can do it, and we get nice organisation and pictures etcetera, but that they have the properties we need.

Session 5: Enamel Pathology

Moderator: Mike Hubbard, University of Melbourne, Australia

#30 DISRUPTION OF STEROID AXIS, A NEW PARADIGM FOR MIH

BABAJKO, S*, JEDEON, K, HOUARI, S, LOIODICE, S and BERDAL, A

#31 FLUORIDE REDUCES KLK4 ACTIVITY, MODULATED THROUGH THE ANDROGEN RECEPTOR

LE, MH*, NAKANO, Y, ZHU, L, ZHANG, Y and DEN BESTEN, PK

#32 DELAYED TIGHT-JUNCTION FORMATION BY FLUORIDE IN A NOVEL AMELOBLAST MODEL

RACZ, R*, BORI, E, FOLDES, A, HARADA, H, STEWARD, MC, DEN BESTEN, P, BRONCKERS, ALJJ and VARGA, G

#33 FLUORIDE IMPACTS IRON METABOLISM DURING AMELOGENESIS ALTERING ENAMEL PROPERTIES

HOUARI, S*, WURTZ, T, VENNAT, E, GUERQUIN-KERN, JL, BERDAL, A and BABAJKO, S

#34 EFFECT OF 4PBA ON ENAMEL MALFORMATION IN DENTAL FLUOROSIS

SUZUKI, M* and BARTLETT, JD

#35 EFFECT OF PRENATAL BPA EXPOSURE OR CHRONIC STRESS TO AMELOBLASTS

DUMAN, C*, ÖZKAN, N and MENTEŞ, A**

*** Presented by A Menteş on behalf of C Duman*

#36 A SMALL GROUP OF GENES TARGETED BY BPA AND FLUORIDE DURING AMELOGENESIS

LOIODICE, S*, HOUARI, S, JEDEON, K, THUY, TT, BERDAL, A and BABAJKO, S

#37 MOLAR HYPOMINERALISATION: A CALL TO ARMS FOR ENAMEL RESEARCHERS

HUBBARD, MJ*, MANGUM, JE and PEREZ, VA

#30 DISRUPTION OF STEROID AXIS, A NEW PARADIGM FOR MIH

BABAJKO, S*, JEDEON, K, HOUARI, S, LOIODICE, S and BERDAL, A

Centre de Recherche des Cordeliers, INSERM UMRS 1138, University Paris-Diderot, Laboratory of Molecular Oral Pathophysiology, Paris, F-75006 France

Email: sylvie.babajko@crc.jussieu.fr

OVERVIEW: Molar-Incisor Hypomineralization (MIH) is a common developmental enamel defect characterized by asymmetric demarcated opacities in permanent molars and incisors. MIH now affects 15 to 20 % of 6 to 9 years old children, whereas it was almost inexistent until the 80s' decade, suggesting that it may result from recent changes of environmental conditions. Its aetiology still remains unclear. However, several causal factors have been proposed as prematurity, viral or bacterial infections, respiratory diseases, asthma and exposure to endocrine disrupting chemicals (EDCs). The present paper identifies a common mechanistic hypothesis that may account for MIH.

EVIDENCE: Enamel hypomineralization has been associated to exposure to dioxin and PCBs, two groups of EDCs. In the same sense, our recent experimental data obtained on rodents exposed to low-doses of bisphenol A (BPA), genistein, vinclozolin, or phthalates (such as DEHP) acting through steroid receptors showed disruption of amelogenesis. In addition, ameloblasts express the incriminated steroid receptors, demonstrating their susceptibility to these molecules. Otherwise, data reported in the literature showed that many, if not all MIH causal factors hypothesized are associated either with medication as corticoids, or exposure to EDCs (premature babies are highly contaminated with BPA and phthalates). Conversely, the secosteroid vitamin D has been proposed to prevent MIH.

CONCLUSION: Many of the proposed causal factors for MIH including EDCs, anti-inflammatory corticoids, and also the possible protection by vitamin D, involve the large family of the steroid receptors. They thus appear as the common elements able to modulate the expression of enamel key genes leading to MIH.

DISCUSSION

Mike Hubbard (Melbourne): I agree that the term MIH was introduced in 2001 but do you think the clinical condition might be a little older than that?

Sylvie Babajko (Paris): It may be. But when we listen to dentists and older ones especially, they never found so much MIH as now. So it really is a change. It's impossible that the prevalence was so high before the 1980s.

Mike Hubbard (Melbourne): I think there'll be some different opinions on that.

Sylvie Babajko (Paris): But we can maybe define more precisely MIH and enamel hypomineralisation?

Elia Beniash (Pittsburgh): People started to see it only in the beginning of the century, and I don't think it's just that they missed it before because it's so obvious a pathology. All kinds of cortisteroids and other things were around for ages, at least for decades. So it looks more like an epidemic,

bacterial or viral, because usually with these diseases, if there is evolution of a bug, it occurs, then you see it everywhere, while all these other things should have been observed before.

Sylvie Babajko (Paris): There is no clear correlation between infections and enamel hypomineralisation, meaning that children may have enamel hypomineralisation without an infection.

Elia Beniash (Pittsburgh): (Without a) *known* infection, because what if it's due to something we don't know about?

#31 FLUORIDE REDUCES KLK4 ACTIVITY, MODULATED THROUGH THE ANDROGEN RECEPTOR

LE, MH*, NAKANO, Y, ZHU, L, ZHANG, Y and DEN BESTEN, PK

Department of Oral and Craniofacial Sciences, School of Dentistry, University of California at San Francisco, San Francisco, CA 94143, US

Email: lem5202@gmail.com

OBJECTIVES: Our aim was to determine whether enamel fluorosis was linked to reduced KLK4 activity in the enamel matrix. We further investigated possible pathways for this effect of fluoride on enamel formation.

METHODS: Ameloblasts and enamel matrix were separately collected at secretory and maturation stages from rats given 0 or 100 ppm F ad libitum in drinking water for 4 weeks. Maturation stage enamel matrix samples were standardized by protein concentration and demineralized in 0.5M acetic acid overnight. Samples were assayed for KLK4 proteolytic activity using a KLK4-specific quencher peptide and analyzed using a fluorescent plate reader. Expression of *Klk4* mRNA in rat maturation enamel organ was compared by qPCR.

To explore the possibility that the androgen receptor (AR) mediates pathways for KLK4 expression, ameloblasts and mandibles were separately collected from C57BL/6 (C57) mice given 0 or 50 ppm fluoride. Expression of *Klk4*, *Ar*, *Ccnd1*, *Tgfbr2*, *Tgfb1*, and *Amel* mRNA was compared by qPCR. Immunostaining for AR, CCND1, TGFBR2, and TGFB1, as well as ameloblast differentiation profiles, were compared between control- and fluoride-treated C57 mice.

RESULTS: *Klk4* mRNA expression ($p<0.001$) and KLK4 proteinase activity ($p<0.01$) were significantly reduced in the fluorosed rat incisor ameloblasts and enamel. In mice given fluoride, AR translocation, and TGFBR2 and TGFB1 synthesis were reduced. Furthermore, there was a significant decrease in mRNA expression of *Ar* ($p<0.01$), *Tgfbr2* ($p<0.05$), and *Tgfb1* ($p<0.01$), no changes to *Amel* expression, and significant increase in mRNA expression of *Ccnd1* ($p<0.01$). Interestingly, fluoride-treated C57 mice had shorter maturation ameloblast layer when compared to control mice.

CONCLUSIONS: Our findings indicate a direct effect of fluoride on *in vivo* KLK4 activity, and mediated through androgen receptor signalling. In addition, our findings also indicate that fluoride affects ameloblast differentiation by inducing early transition of maturation stage that results in reduced *Klk4* expression.

DISCUSSION

John Bartlett (Ohio): You tested one side of the equation where you showed that KLK4 expression was decreased but you didn't test the other side to see if KLK4 activity was decreased. Did you test that? In other words, was the enzymatic activity in an individual KLK4 molecule decreased by fluoride exposure?

Michael Le (San Francisco): In vivo or *in vitro*?

John Bartlett (Ohio): Either

Michael Le (San Francisco): *In vitro* it's been shown that mixing fluoride and KLK4 doesn't result in an effect. Most of my work was focused just on ameloblasts so it would be interesting to see if that is really the case in the matrix side as well.

#32 DELAYED TIGHT-JUNCTION FORMATION BY FLUORIDE IN A NOVEL AMELOBLAST MODEL

RACZ, R[1]*, BORI, E[1], FOLDES, A[1], HARADA, H[2], STEWARD, MC[3], DEN BESTEN, P[4], BRONCKERS, ALJJ [5] and VARGA, G[1]

[1]Department of Oral Biology, Semmelweis University, H-1089, Nagyvarad ter 4, Budapest, Hungary; [2]Department of Anatomy, Division of Developmental Biology & Regenerative Medicine, Iwate Medical University, Iwate, Japan; [3]Faculty of Life Sciences, The University of Manchester, Manchester, M13 9PT, UK; [4]Department of Orofacial Sciences, University of California, San Francisco, USA ; [5]Department of Oral Cell Biology, Academic Centre for Dentistry Amsterdam (ACTA), University of Amsterdam and VU University Amsterdam, MOVE Research Institute, Amsterdam, the Netherlands

Email: racz62@gmail.com

OBJECTIVES: We have recently developed a novel *in vitro* model using HAT-7 ameloblast cells to functionally study epithelial ion transport during amelogenesis (JDR 95:588-96, 2016). Our present aims were to examine HAT-7 cell viability, the development of transepithelial resistance and also vectorial bicarbonate transport during fluoride exposure.

METHODS: To obtain monolayers, the HAT-7 rat ameloblast cells were cultured on Transwell permeable filters. We monitored transepithelial resistance (TER) as an indicator of tight junction (TJ) formation and polarization. We evaluated intracellular pH changes by microfluorometry using BCECF fluorescent indicator. Ion transporter activities were tested by withdrawal of various ions from the outer solutions, and by using transporter specific inhibitors, and by activation of transporter activities by forskolin and ATP. Cell survival was estimated by WST-1 assay.

RESULTS: Measurements of transepithelial bicarbonate transport showed a significant increase over control (-0.36 ± 0.09 mM/min) bicarbonate flux in response to simultaneous Ca^{2+}- and cAMP-mobilizing stimuli (ATP and forskolin, -1.22 ± 0.18 mM /min) ($p<0.05$). Acute 30 -1000 μM fluoride exposure did not significantly affect stimulated bicarbonate secretion. Cell viability was decreased by 56 ± 2% when 1000 μM fluoride was applied for 24 h, but concentrations up to 300 μM were ineffective, while 3000 μM and higher doses were totally toxic. However, we detected a 1-2 days delay in TJ formation by 300 μM fluoride, whereas 600 μM caused a 4-8 day shift in TER development.

CONCLUSION: HAT-7 cells can be functionally polarized and are able to transport bicarbonate ions from the basolateral to the apical direction. This vectorial transport is not affected by a wide range of acute fluoride exposure. 24 h fluoride reduced cell viability in the dose range observed before. However, the formation of tight junctions was inhibited by doses which do not induce cell death, indicating a novel, previously unknown mechanism contributing to dental fluorosis.

ACKNOWLEDGEMENTS: Supported by NIH-NIDCR 5R01DE013508 subaward: 7743sc.

No questions

#33 FLUORIDE IMPACTS IRON METABOLISM DURING AMELOGENESIS ALTERING ENAMEL PROPERTIES

HOUARI, S[1]*, WURTZ, T[1], VENNAT, E[2], GUERQUIN-KERN, JL[3], BERDAL, A[1] and BABAJKO, S[1]

[1]Centre de Recherche des Cordeliers, INSERM UMRS 1138, Team Berdal, Laboratory of Molecular Oral Pathophysiology, Paris, F-75006 France; [2]Centrale Supelec, Laboratoire MSSMat, 92295 Chatenay Malabry Cedex, France; [3]Institut Curie, INSERM U759, 91405 Orsay Cedex, France

Email: sophia.houari@crc.jussieu.fr

OBJECTIVES: The aim of the present study is to analyze the relationship between excessive fluoride intake and iron metabolism in ameloblasts in order to explain the adverse impact of fluoride on enamel mechanical, optical and biochemical properties.

METHODS: We used Balb/c mice to highlight the *in vivo* targets impacted by chronic exposure to 5 mM sodium fluoride in the drinking water over 6 weeks. The specific iron accumulation was followed by Perl's staining and the sensitive nanoSIMS imaging technique. Immunofluorescent assays were used to localize the components involved in iron metabolism such as Heavy chain Ferritin (FTH1), ferroportin, DMT-1 and transferrin receptor- 1 (TFR). In addition, their expression levels were measured by RT-qPCR and Western Blot analyzes. The mechanical properties of human and mice control and fluorotic enamel were analyzed using nanoindentation.

RESULTS: Human and mouse fluorotic enamel presented severe hypomineralization area and reduced hardness. Our results clearly showed reduced iron storage within the mature ameloblasts following fluoride chronic treatment. The reduced *Fth1* and increased ferroportin mRNA and protein levels may explain the lower capacity of iron storage in ameloblasts and the higher exportation of iron outside the cells, both on the distal and proximal poles of mature ameloblasts. Interestingly, fluoride was also able to reduce the iron storage in mouse liver and intestine.

CONCLUSION: Iron storage, which is involved in enamel hardness, was clearly reduced by chronic exposure to fluoride. The relationship between fluoride and iron metabolism is here reported for the first time. The decreased iron storage upon fluoride treatment may explain the decreased enamel hardness and mineralization. This reduced iron storage was also observed in the liver and in the intestine, which suggests a general perturbation of iron metabolism by fluoride arguing for the use of dental defects as a biomarker of exposure to environmental conditions.

DISCUSSION:

Mike Hubbard (Melbourne): For species such as humans that don't have pigmented enamel, what do you think is happening with the iron transport? Do you think it's relevant to species without pigmented enamel?

Sophia Houari (Paris): Because on human teeth we can see brown coloration in areas, the hypothesis is that the brown areas have high amounts of iron but that's not been demonstrated.

Alex Vieira (Pittsburgh): Regarding the argument that you have a mutation in a particular gene, the *FTH1* gene, do you know the frequency of variance there? Is there anything we know about it?

Sophia Houari (Paris): In humans?

Alex Vieira (Pittsburgh): Yes

Sophia Houari (Paris): There is just one deletion of this gene reported and the patients had perturbation of iron status in their blood. But anything about their teeth is unknown at the moment.

#34 EFFECT OF 4PBA ON ENAMEL MALFORMATION IN DENTAL FLUOROSIS

SUZUKI, M* and BARTLETT, JD

Division of Biosciences, College of Dentistry, The Ohio State University, 305 W. 12th Ave., Columbus, OH, USA

Email: Suzuki.94@osu.edu

OBJECTIVES: We previously reported that fluoride induces endoplasmic reticulum (ER) stress and oxidative stress in ameloblasts. Cell stress impairs ameloblast function, leading to dental fluorosis characterized by porous, soft enamel that is vulnerable to erosion and decay. Sodium phenylbutyrate (4PBA) is a histone deacetylase inhibitor (HDACI) and is also a chemical chaperone that interacts with mis-folded proteins to prevent ER stress. To determine whether 4PBA protects ameloblasts from fluoride toxicity, we quantified gene expression, fluorosis levels and enamel hardness.

METHODS: Ameloblast derived cells (ALC) were treated with fluoride (5 mM) with or without 4PBA (1 or 5 mM) for 24 h. Expression of *Tgf-β1*, *Bcl2* and *Bax* were then quantified by real-time PCR. Sprague-Dawley rats and C57BL/6J mice (6-week-old) were provided water *ad libitum* containing fluoride (0, 50, or 100 ppm, N=5/group) and were fed for six weeks with either fluoride-free or 4PBA-containing chow (7g/kg).

RESULTS: Fluoride treated ALC cells had decreased *Tgf-β1* expression that was reversed by 4PBA treatment. The *Bcl2*/*Bax* ratio was significantly increased in ALCs treated with fluoride/4PBA compared to fluoride treatment alone. These results suggest that 4PBA mitigates fluoride-induced gene suppression and apoptosis *in vitro*. However, in general, significant differences were not observed *in vivo.* With few exceptions, enamel microhardness, fluorosis levels, and bone, urine and plasma fluoride concentrations did not differ significantly between fluoride treated animals fed with fluoride-free or 4PBA-containing chow.

CONCLUSION: 4PBA reversed fluoride-induced gene repression and increased anti-apoptotic gene expression *in vitro.* However a diet rich in 4PBA did not attenuate dental fluorosis in rodents. Perhaps, not enough intact 4PBA reaches the rodent ameloblasts necessary to reverse the effects of fluoride toxicity.

<u>DISCUSSION:</u>

Mike Hubbard (Melbourne): How do you distinguish the effects of 4PBA on histone deacetylase from its chemical chaperone effect?

Maiko Suzuki (Ohio): I think that the 4PBA inhibitor function for histone deacetylase also contributes to the amelioration of fluoride-induced separation of the TGF-beta reduction.

Mike Hubbard (Melbourne): In the cells but maybe not in the animal?

Maiko Suzuki (Ohio): I think both, I hope.

Alex Vieira (Pittsburgh): The results of the hardness for the maxilla and mandible at 100 ppm fluoride look quite different. Do you know why?

Maiko Suzuki (Ohio): The number of mice in each group is only 5 so I think this huge error bar is due to the small sample number.

Alex Vieira (Pittsburgh): Why is the 100 ppm fluoride value so low versus the other one?

Maiko Suzuki (Ohio): I'm not sure sorry.

#35 EFFECT OF PRENATAL BPA EXPOSURE OR CHRONIC STRESS TO AMELOBLASTS

DUMAN, C[1]*, ÖZKAN, N[2] and MENTEŞ, A[1**]

[1] Department of Paediatric Dentistry, Marmara University, İstanbul, Turkey: [2] Department of Pathology Laboratory, Marmara University, Istanbul, Turkey

*** Presented by A Menteş on behalf of C Duman*

Email: drcananduman@gmail.com

OBJECTIVES: Enamel disturbances have been widely observed in children and the aetiology may rely on both genetic and environmental factors during amelogenesis. This study evaluated immunohistochemical staining of amelogenin and ameloblastin in mandibular first molars of rats exposed to one of two environmental factors prenatally, such as bisphenol-A (BPA) or chronic stress (CS).

METHODS: 9 pregnant rats were randomly divided into 3 groups as BPA, CS and control. In BPA group, rats were carried to glass cages and fed by bottles made of glass to avoid BPA and phthalates contamination. From gestational day E1 until weaning, pregnant females were orally administered daily 5 µg/kg BPA that was dissolved in corn oil and added to drinking water. In CS and control groups, rats were placed to standard cages and provided with tap water ad libitum. In CS group, from day E18 until the end of the pregnancy, rats were exposed chronically to a 12:12 light/light cycle (LL) in order to create CS. All BPA, CS and control groups were fed with standard diet. Seven pups from each group were breastfed until day P10 when they were euthanized. Lower jaws were immediately fixed in 10% formalin and after decalcification, embedded in paraffin. Sections were incubated with rabbit polyclonal antibodies to AMELX (ab59705, Abcam, Cambridge, UK), and to AMBN (bs-12467R, Bioss Antibodies, Massachusetts, USA) and staining intensity of ameloblast stages of first molars were evaluated microscopically.

RESULTS: In BPA and CS groups, amelogenin staining was significantly lower in secretory, transitional and maturation stages of ameloblasts (p= 0.0048, p= 0.0019, p= 0.0453 respectively) than control, whereas ameloblastin expression significantly lower only in maturation stage (p= 0.0281).

CONCLUSION: This study suggests that BPA intoxication or CS in pregnancy of rats interfere with enamel protein expression in their puppies at the different stages of ameloblasts.

DISCUSSION:

Jason Wan (Bethesda): I just have a question about the chronic stress. What was the condition you were administering that with?

Ali Menteş (Marmara): The light exposure – we don't allow the rats to sleep. This is one way to get stress in rats. There are some other ways to do it but if rats are pregnant, people say that this is the best way to get the stress for the animal.

Brian Clarkson (Michigan): Why did you use 5 micrograms of BPA?

Ali Menteş (Marmara): We looked at the literature; there are recent studies that say this is a good amount to use. Actually we used BPA as a control. Recent studies show there is an enamel defect with the BPA. We wanted to see whether stress has the same effect as the BPA.

Sylvie Babajko (Paris): This is a low dose that corresponds to the environmental doses.

#36 A SMALL GROUP OF GENES TARGETED BY BPA AND FLUORIDE DURING AMELOGENESIS

LOIODICE, S[1]*, HOUARI, S[1], JEDEON, K[1], THUY, TT[2], BERDAL, A[1] and BABAJKO, S[1]

[1]Centre de Recherche des Cordeliers, INSERM UMRS 1138, University Paris-Diderot, Laboratory of Molecular Oral Pathophysiology, Paris, F-75006 France; [2]Faculty of Odonto-Stomatology, Hô-Chi-Minh University of Medicine and Pharmacology, Hô-Chi-Minh Ville, Viêtnam

Email: sophia.loiodice@crc.jussieu.fr / sylvie.babajko@crc.jussieu.fr

OBJECTIVES: The aim of the present study was to determine the genes involved in enamel hypomineralization resulting from environmental conditions in order to characterize molecular events leading to specific enamel defects.

METHODS: Wistar rats were chronically exposed to 5 µg/kg/d of bisphenol A (BPA) from day 1 of gestation to day 65 after birth (P65) in combination, or not, with 5 mM fluoride from P21 to P65. Both agents are commonly encountered in human populations. Dental epithelia were microdissected and RNA submitted to micro-array analysis. Incisors were analyzed by micro computed tomography (µCT) and scanning electron microscopy (SEM).

RESULTS: First, resulting enamel defects were comparable to human enamel pathologies molar incisor hypomineralization (MIH) and dental fluorosis (DF), and were more severe in rats exposed to both agents. Second, large-scale transcriptomic analysis of dental epithelium evidenced a small group of genes for which expression was affected by exposure to BPA or NaF: Among the 19,239 genes analyzed, few were significantly ($p < 0.05$) differentially expressed (more than 1.5-fold) in the BPA (19), NaF (78) and BPA + NaF (135) group compared to the control group. Among those most modulated, many are directly involved in amelogenesis (those encoding enamel matrix proteins, including amelogenin and enamelin, proteases, mainly *KLK4*, and ion-transporters and exchangers). Each of these perturbations to gene expression may contribute to enamel defects.

CONCLUSION: Exposure to BPA weakens enamel, making it more susceptible to frequent mineralization defects, MIH and DF. Our study identifies a small group of genes involved in systemic enamel hypomineralization, some of them are also reported in genetic enamel pathologies.

DISCUSSION:

John Bartlett (Ohio): I just wanted to make a comment. There is a lot of similarity between the various MMPs and I'm not aware that MMP9 is expressed in ameloblasts. You might have been cross reacting with MMP20. So just be careful with your antibody selections.

#37 MOLAR HYPOMINERALISATION: A CALL TO ARMS FOR ENAMEL RESEARCHERS

HUBBARD, MJ[1 2]*, MANGUM, JE[1] and PEREZ, VA[1 3]

[1]Department of Pharmacology & Therapeutics, The University of Melbourne, Victoria 3001, Australia; [2]Department of Paediatrics, The University of Melbourne, Victoria 3001, Australia; [3]School of Dentistry, University of Talca, Talca, Chile

Email: mike.hubbard@unimelb.edu.au

OVERVIEW: Developmental dental defects (DDDs, hereafter D3s) hold significance for scientists and practitioners from both medicine and dentistry. Although attention has classically dwelt on three other D3s (amelogenesis imperfecta, dental fluorosis and enamel hypoplasia), dental interest has recently swung towards molar hypomineralisation (MH), a condition characterised by demarcated enamel opacities. MH imposes a comparatively high burden on global health and, being linked to infantile illness, has good potential for medical prevention. Yet awareness of this childhood problem and allied research opportunities remains narrow, including within the research community. Major knowledge gaps exist at population, case and tooth levels, including quandaries surrounding the pathogenesis and biophysical makeup of demarcated opacities. Moreover, salient contributions by enamel researchers have sometimes been omitted from clinically-oriented conclusions.

EVIDENCE: Aiming to ultimately prevent MH and the associated risk for dental caries, a cross-sector network group was recently established across Australia and New Zealand. This translational network (The D3 Group) seeks better understanding and care of people with D3s through improved research and education. Initial education efforts comprised a comprehensive online education resource (www.thed3group.org) and a public-friendly awareness campaign (www.chalkyteeth.org). Affiliated researchers have elucidated MH at population, case and tooth levels. One ongoing study, involving a team of biochemists and dentists, is producing novel insights to the pathogenesis and diagnosis of "chalky enamel".

CONCLUSION: A cross-sector translational approach has already proven beneficial at educational and research levels. Further internationalisation of this network seems worthwhile given its potential to use an unprecedented set of capabilities to attract high-level resourcing. For this to work, diverse researchers (basic science, paediatric medicine, dentistry, allied industry and public health) must be attracted to this nascent field. A particularly strong need exists for more enamel researchers to join the worthy fight against MH.

DISCUSSION:

Pam Den Besten (San Francisco): What is your hypothesis for the cause, you must have some standing hypothesis that you are considering?

Mike Hubbard (Melbourne): Yes we are. We're not there yet but we have been trying to find an answer to the big problem for the field – "is it the disease, or is it the treatment of the disease?". There's been a lot of interest in maybe antibiotics causing MIH. If that was the case you should be able to set up animal models. You should be able to look at the prevalence of molar hypomineralisation versus time when various antibiotics came on board. You may be able to go back

further in the literature to find earlier reports of the condition before it was called MIH. When one-in-six kids are affected, it's clearly something very common. If you did start to find out what the cause is, either a single factor or a combination of things, you may need to be careful, ethically, not to disturb young parents who could be scared that their child could get this problem. When researchers make sufficient breakthrough to talk about it, that information might have to be managed very carefully. It could be better to get some handle on prevention, or preventability, before saying this is the cause.

Elia Beniash (Pittsburgh): If you don't know the cause, how can you prevent it?

Mike Hubbard (Melbourne): Our ambition is to generate interest and research funding so that the cause plus the interventions can be sorted out.

Elia Beniash (Pittsburgh): So you did the proteomics? What did you find there?

Mike Hubbard (Melbourne): That was a paper published in 2010, which was spoken about a little at the last Enamel Symposium. When you do proteomics on the chalky enamel lesions, what we reported is that we've been able to find albumin just in the location of the chalky enamel. Colin Robinson has done a lot of background work in that area and the question really was "is that a normal constituent or not?", because other people have reported finding albumin in normal enamel. We have had to work through that and there will be something published soon. So having found albumin there, the question then becomes "where did it come from, are there other tissue-fluid proteins there or not?". And as a loose answer, the answer is yes.

Yong-Hee Chun (Texas): Can you comment on the genetic contribution to MIH? I'm thinking in regards to SNPs, maybe susceptibility genes but also epigenetics?

Mike Hubbard (Melbourne): Yes it's a question that crops up often, usually in context of the obvious genetic cases such as AI. So the first answer is no, we are not anticipating that there is a primary monogenetic cause. But with modern genetics people are now looking at multi-allelic and multi-gene effects, so obviously there will be genetic contributions, it's just questionable how much these affect 1 in 6 kids. Sibling studies haven't been done yet, twin studies are on the way. I imagine that genetics will have a contributory effect but I don't think it will be that dominant when the prevalence is 1 in 6.

PANEL DISCUSSION:

Alex Vieira (Pittsburgh): I would like to feedback on the question just made about molar hypomineralisation. I just published a case in *Caries Research* that it is indeed a genetic defect and I truly think it behaves as a complex disease. The frequency of MIH is as high as cardiovascular diseases, as high as asthma, as high as cancer. It has all the features of a complex disease that has, in all likelihood, an environmental input. I think that's the way we should start thinking about this problem. It's not surprising to me that nobody has identified a causal or environmental factor. I still get confused when, for example, Sylvie lists asthma and things like that as possible environmental factors, because asthma is actually a genetic disease. It is a complex disease and multiple genes are involved in all likelihood. The environment may play a role and it might be less important to actually worry too much about it because then we can go ahead and try to address it (genetically).

Sylvie Babajko (Paris): So it is really a complex question and that's why maybe my answer will be also difficult to understand, but I hope that I will be quite clear. Probably MIH is a complex disease and it probably is associated with SNPs. It has been published that a SNP in enamelin affects susceptibility to dental decay so it's also possible here that there are such SNPs in enamelin or maybe other genes. But also concerning the 1 in 6 prevalence and environmental factors, maybe there are different causes also. Concerning the endocrine disruptors or pollutants in general, there are many things to consider. First, the body's detoxifying system is not major in children, explaining the fact that young children are more susceptible to environmental pollutants than are adults. And also concerning genetics, we are not all exposed to environmental pollutants to the same extent. We talk about teeth, but in general, all these molecules also affect other cells. I spoke about the detoxifying system, but other genes more generally expressed can also be involved in the pathology. That's why the prevalence is high because of the different causal factors. I proposed the steroid axis because there are many molecules involved, including vitamin D and vitamin E. And so probably the association between these factors, as in the study here of fluoride and bisphenol A, is one point. But earlier reports addressed associations with antibiotics and fluoride and also dioxin. That's why it is probable that there are different causal factors included in MIH as well as our genetic background with SNPs in enamelin or other genes that can explain such prevalence.

Per Ahlberg (Uppsala): First of all, coming into this as something of a layman, I'm horrified to encounter a problem that I wasn't really aware of but *is* obviously of major importance. A key thing here is to get a picture of what the historical levels of prevalence have been. We're all aware we are living in a pollution landscape that has shifted radically over recent decades and become more complex. It really is going to be important to see when the rise in this condition started and exactly by how much, because historical association with changes in the pollution landscape is going to be critical to understanding what's going on today.

Mike Hubbard (Melbourne): We agree.

Bernhard Ganss (Toronto): My question is for Sylvie and probably for Mike. In your global map of MIH prevalence; I've noticed there's some significant heterogeneity in there. Is there something that

we can learn from that, something that we can ask questions about or start knocking at the doors of our dental public health folks? What do we do in North America that we don't do in Europe or the other way around?

Mike Hubbard (Melbourne): Yes, everything you said is pertinent and I think there's a growing number of North Americans interested to get some answers. Indeed there's one about to ask a question.

Yong-Hee Chun (Texas): Definitely MIH is also prevalent in North America. In Texas, we've just completed a study on the prevalence. But my understanding is that the awareness started out in Europe and Scandinavia, and it's been dramatically increasing. So I think that's one of the issues that we've been facing in the clinics- that the awareness hasn't been there yet. It's also my understanding that, clinically, MIH is classified in four categories by the European Academy of Paediatric Dentistry and I think that's the most accepted classification at this point. So the mildest form that you mentioned involves chalky defects that are demarcated by sound enamel. So to me that's really the hallmark of it, where it all starts, and then from there it deteriorates through porosity of the enamel surface and progression into more clinically relevant forms. Now I'm really happy to see that we're having some animal models mimicking MIH, and I was wondering if you could comment on to what extent demarcation of defects and porosities are reflected in your animal model?

Sylvie Babajko (Paris): Your question is about the porosity of enamel?

Yong-Hee Chun (Texas): Yes about porosity and demarcations.

Vidal Perez (Melbourne): I'm a paediatric dentist and the condition is defined based on the molar being the most-affected tooth. So a second question is, in your animal model, how do you generate demarcated opacities in molars?

Sylvie Babajko (Paris): Of course there are different things that have been discussed and questions raised about the heterogeneous properties of the original pathology and the animal model in rodents. Of course rodents don't have premolars and it is very important that premolars are almost never, if not never, affected. They are the last teeth to mineralise, and it is really important to take into account that point. So it is an animal model and it hasn't reproduced everything. But molars in rats are also affected, as we have shown in our 2013 paper in the American Journal of Pathology.

Pamela Den Besten (San Francisco): I was really struck just now by the description of molar hypomineralisation as potentially being a combination of genetic and environmental effects and I would say the same thing goes for fluorosis. There is a very big range of susceptibility to fluorosis. It's clearly caused by environmental effects and it is clearly affected by other environmental factors and I think it's time in enamel biology or dentistry we think of these things as multi-causal and elevate their importance, as Mike said.

Raquel Gerlach (Sao Paulo): I just wanted to say that many diseases cause fever and cell stress that will result in enamel defects that sometimes are not seen. It has to be very carefully taken into account when people describe prevalence of genetic diseases that may cause caries. First of all, the prevalence of these other defects and the stress and all that has to be taken into account. I think that's very important.

Mike Hubbard (Melbourne): I agree.

Ali Menteş (Marmara): I'm not that old, I graduated in 1985, but I've since seen many children in Turkey with that kind of defect, especially in first molars. And I've always thought, before 2001 when MIH was first described, that this is because of fevers that happened just after birth. Many children, especially in my country, get some kind of infection just after birth. So we always thought this is the same time that first molars get mineralised so this is the right time. We always ask parents if their child had something wrong in the first year of life and they always say yes. And pollution in Turkey is probably quite late maybe. I'm thinking that infection is also probably a huge impact with the MIH defects. We are trying to get infected pregnant rats and get the same thing, but it's quite difficult I think. We are also looking to affect only the molars, not the incisors, because it's quite different and the enamel is quite different. So I think the environmental factor is quite important in MIH rather than genetics. This is my opinion.

Iris Frasheri (Munich): Professor Kunis from our University has been studying more than 300 teeth from the 12th, the 16th and the 18th centuries and they have seen involvement of MIH around in about 3.1 %. So it's much lower than today's percentage. So it might be there are actual conditions in the modern ages that are also involved. But from the genetics, how can this 3% explain what was previously there?

Mike Hubbard (Melbourne): That's a good question.

Henry Margolis (Forsyth): How is prevalence monitored? What are the conditions for prevalence? So my recollection is that a lot of the caries prevalence data involves decayed missing and filled surfaces. So white spots, which may be comparable to MH, are not counted. So if you *did* count white spots in caries prevalence data that number may be much higher. The prevalence of 1 in 6 is really shocking, so I just want to know, in the prevalence data that you've quoted from 59 different locations, how is that done? What is the basis?

Vidal Perez (Melbourne): Usually the caries white spots are located in the approximal surfaces between the teeth, is that your question?

Henry Margolis (Forsyth): No, I'm asking when you do MH prevalence, and I accept that you can distinguish a white spot in MH from a caries white spot, when you count that, what happens? You

showed some images with the enamel surface being completely destroyed so I'm just wondering when we compare MH prevalence to caries prevalence that doesn't count white spots, what would happen to those numbers in terms of our understanding of MH? You're catching it at a very incipient level, would that be correct? But then it may be susceptible to caries?

Vidal Perez (Melbourne): There are two manifestations that are really clear. One is the demarcated opacity with no broken enamel and then there's the post-eruptive breakdown that could be confused with caries. I think that is the distinction we make, we see if it's broken or not. And usually the location of the opacity is far from the gingiva, in other words the cusp is usually affected in MH.

Henry Margolis (Forsyth): But do you count both?

Mike Hubbard (Melbourne): No, not in MH. I think the problem is when you are dealing with DMFT, which is the public health standard round the world, it takes no account of molar hypomineralisation. But the molar hypomineralisation prevalence distinguishes caries – two different assessments. So the projection from that is, if DMFT actually accounted for molar hypomineralisation, which is basically immune to fluoride protection, the efficacy of fluoridation would be seen to go up. So it's something that should be fixed at public health level.

Ariane Berdal (Paris): The increase of enamel defects as a whole is not an isolated process in health. There is a modern birth of disease – diabetes, obesity and so on – and the relationships between pollution and endocrine disruptors and the way we live now is clearly established for male infertility. How did it happen? In France we have a national action about health problems and endocrine disruptors and also other factors. So in that light, all these enamel defects may just be a public health defect of many other systems. And then the question will become, since we have little awareness that fluoride may have general effects, and if we consider the data on fluoride and androgen receptors, what do you think would be the level of fluoride that may be affecting other systems besides ameloblasts? Or do you think that the ameloblasts probably are extremely sensitive? What would be your opinion about the general prevention that we could still do now, just taking fluoride, and does it actually contribute to the pollution atmosphere we all live in today. What is your opinion, should we change our practice?

Michael Le (San Francisco): So if I understand the question correctly, with some of the things we are finding with fluoride, how do we use fluoride as a tool? How can we best use it in dentistry today, is that the kind of thing?

Ariane Berdal (Paris): Yes

Michael Le (San Francisco): My personal opinion is that we need a lot more studies to figure out what it's doing. When it comes to fluoride and teeth, it's the most obvious thing that we can see. But more studies need to be done on fluoride's other systemic effects since we are involving it in the steroid axis with testosterone and such. Is there an effect on reproduction, is there an effect on kidney

function, is there an effect on liver? More studies need to be done to understand if that's the case. I think, that said, it's up to the public to figure out how they feel about the value of this and what fluoride is trying to fight. Is fluoride helping certain individuals in the population? I think so. Is it hurting other people at the same time? I think yes to that too. We also need to figure out who is going to be more adversely affected than others and try to understand these mechanisms of how fluoride works and identify the individuals that will, or will not benefit. I think that's what we need to elucidate much better and I think some of the discussions we have, especially with MIH, is that there's certain individuals that will get this and what seems an exactly equivalent individual will not. What are those factors and what are those things requires, perhaps a much more balanced approach than the candidate gene approach we tend to focus on. And using the full data that we have outside, not just the snapshots we can get through high throughput sequencing and such, and comparing that and using it collectively to then figure out what's happening overall.

Stephen Hsu (Singapore): It looks like there may be a specific timing issue regarding MIH. Has anyone collected perinatal data because those developments occur during a specific time-window period? For those countries that have very good perinatal data covering how they treat people, the delivery systems used and the kind of medications that can be given, has any of this perinatal data been collected and considered as potential factors that could contribute to this.

Mike Hubbard (Melbourne): I think that's a good idea that we should talk about later. Have we got some questions on maybe the toxicology side of things?

Brian Clarkson (Michigan): Could you explain to me how systemic pollution and/or genetics affect just one tooth?

Mike Hubbard (Melbourne): You've asked my favourite question! That is a highly salient question and we have some answers on the way – but I can say now, I think it's inappropriate to describe MH as a systemic disorder.

Raquel Gerlach (Sao Paulo): We have co-exposed rats to fluoride plus lead and the levels of lead tripled. Lead is a neurotoxin, it's actually the best known neurotoxin unfortunately. This has been known since the Roman times, when England was invaded because they needed lead. The levels tripled in blood, dentine, bone and in the enamel. And the co-exposed animals were worse. They didn't get fluorosis as it's classically seen in incisors. They had other kinds of lesions that reminded us of hypomineralisation. We looked into the fluorosis literature over the years in Europe and what was described as fluorosis with pits and other kinds of things, looks to us like multiple agents are contributing to an enamel defect that's not just a defect caused by fluoride. Even this morning people were co-exposing animals. Lead is also a known cause of endocrine disruption. So it's becoming very difficult to defend using fluoride in the water. It's impossible to defend that idea so I challenge you to read the literature, because fluoride is already linked to decreased IQ in children around the world – ten points is a lot – and because it increases lead, which is a known neurotoxin. I think we should be very careful about suggesting putting such agents in the water.

Mike Hubbard (Melbourne): Thank you Raquel.

Agnès Bloch-Zupan (Strasbourg): This is more of a comment because I'm sure many of you are aware, there is a similar disease in the primary dentition with second molars being affected with some kind of hypomineralisation. So that may add to or complicate the understanding of what's happening.

Mike Hubbard (Melbourne): An important point.

Alex Vieira (Pittsburgh): Let me comment on how one tooth can be affected by an overall exposure to an individual. Dentitions are actually a very good example on how to figure those things out. Our teeth don't look the same, and in all likelihood, it's because they show a different pattern of gene expression. There's also a timing difference during their development. So when you put all this together in the equation with exposure that is constricted to a window, you can actually argue that there might be specific organs that are affected but not others. Also, when you comment that genetics may be less important, I wouldn't think that way. I would actually think there is a genetic background that is impacted by the exposure. So a 1 in 6 frequency means that a lot of people have been exposed, but not everybody is demonstrating the disease because of their specific genetic background, that means they are more susceptible than the next one and so forth. And that's kind of the framework I think it would be more relevant for us to work on. DNA would play less importance, don't worry about all those genes because we may never find them. But let's work on how to address the problem. That's kind of how I think about it.

Mike Hubbard (Melbourne): We need to draw the session to a close but I would like to solicit questions on how we are approaching the field. We used to have just fluoride, and now we've got all these other things coming in and we're thinking about genetics etc. The question was raised about where does 5 micrograms per kilogram per day come from, which is a very small amount when you think of the weight of a mouse compared to a kilogram. So where do those numbers come from? Historically, a lot of fluoride research has been done without very much thought as to what the *in vivo* exposure is. The amount of fluoride in our blood, or in our tissue fluids, is nothing like the millimolar concentrations that people are throwing into cell culture dishes. So what do people think about the need to maybe get a bit more aware of bringing in the physiological, or pathophysiological, element to this field?

Per Ahlberg (Uppsala): I just wanted to add a final response to the last point that was taken up here. If you find suddenly now that a substantial part of the human population is responding to some environmental provocation, which would appear to be the case, this is the sort of thing that has the absolute hallmarks of a new environmental problem having appeared. It is making visible a genetic polymorphism that in the past was of no significance. Yes of course there is the genetic basis, but what it's actually showing is that there is a new environmental problem that's popped up. I think it's very important to grasp that point.

Session 6: Amelogenesis Imperfecta

Moderator: Agnès Bloch-Zupan, University of Strasbourg, France

#38 AMELOGENESIS IMPERFECTA: LESSONS FROM NGS

PRASAD, MK, MANIERE, MC, DE LA DURE-MOLLA, M, HUCKERT, M, DOLLFUS, H, BERDAL, A and BLOCH-ZUPAN, A*

#39 AMELOGENESIS IMPERFECTA: GENETIC ETIOLOGY AND DISTINGUISHING ISOLATED FROM SYNDROMIC CASES

ZHANG, H and HU, JC-C*

#40 AMELOGENESIS IMPERFECTA GENETIC INFORMATION – WHAT USE TO PATIENT CARE?

SMITH CEL, POULTER JA, INGLEHEARN CF and MIGHELL AJ*

#41 FUNCTIONAL AND ESTHETIC REHABILITATION OF A PATIENT WITH ODDD SYNDROME

FRASHER, I*, PALME, S, MANHART, J

#42 CRYSTALLOGRAPHY AND MICROSTRUCTURE OF ENAMEL AFFECTED BY HYPOMATURATION AMELOGENESIS IMPERFECTA

SIDDIQUI, S and AL-JAWAD, M*

#43 NOVEL *KLK4* VARIANT ASSOCIATED WITH ENAMEL HYPOMINERALISATION AND STRUCTURAL ABNORMALITIES

SMITH, CEL*, BROOKES, SJ, DAY PF, POULTER JA, INGLEHEARN, CF, MIGHELL, AJ and KIRKHAM, J.

#44 *MMP20* MUTATIONS CAUSING ENAMEL HYPOMATURATION IN TWO FAMILIES

KIM, YJ, KANG, J, SEYMEN, F, KORUYUCU, M, GENCAY, K, HU, JC, SIMMER, JP, KIM, J-W

#45 FAM20A GENE MUTATION: AMELOGENESIS OR ECTOPIC MINERALIZATION?

LIGNON, G*, BERES, F, ROUZIÈRE, S, MOUTON, L, DE LA DURE-MOLLA, M, DESSOMBZ, A and BERDAL, A

#46 ER STRESS AS AN AETIOLOGICAL FACTOR IN AMELOGENESIS IMPERFECTA

BROOKES, SJ*, BARRON, MJ, SMITH, CEL, POULTER, JA, INGLEHEARN, CF, MIGHELL, AJ, DIXON, MJ AND KIRKHAM, J

#38 AMELOGENESIS IMPERFECTA: LESSONS FROM NGS

PRASAD, MK[1], MANIERE, MC[2 3], DE LA DURE-MOLLA, M[4 5], HUCKERT, M[1 2 3], DOLLFUS, H[1], BERDAL, A[4 5] and BLOCH-ZUPAN, A[2 3 6 7 8]*

[1]Laboratoire de Génétique Médicale, INSERM U1112, Fédération de Médecine Translationnelle de Strasbourg (FMTS), Université de Strasbourg, France; [2]Université de Strasbourg, Faculté de Chirurgie Dentaire, Strasbourg, France; [3]Hôpitaux Universitaires de Strasbourg (HUS), Pôle de Médecine et Chirurgie Bucco-dentaires, Centre de Référence des Manifestations; Odontologiques des Maladies Rares O RARES, Strasbourg, France; [4]Laboratoire de Physiopathologie Orale Moléculaire INSERM UMR S1138 Centre de Recherche des Cordeliers, Universités Paris-Diderot et Paris-Descartes, Paris, France; [5]Centre de Référence des Malformations Rares de la Face et de la Cavité Buccale MAFACE, Hôpital Rothschild, Pôle d'Odontologie, Paris, France; [6]Université de Strasbourg, Institut d'Etudes Avancées, USIAS, Strasbourg, France; [7]Institut de Génétique et de Biologie Moléculaire and Cellulaire-Centre Européen de Recherche en Biologie et en Médecine, Université de Strasbourg, IGBMC-CERBM CNRS UMR7104, INSERM U964, Illkirch, France; [8]Eastman Dental Institute, UCL, London, UK

Email: agnes.bloch-zupan@unistra.fr

Amelogenesis Imperfecta (AI) is a clinically and genetically heterogeneous group of diseases with enamel defects. To date, mutations in >20 genes have been implicated in either isolated or syndromic AI.

OBJECTIVES: We investigated the genetic aetiology responsible for the various phenotypes presented by a cohort of AI patients.

METHODS: Patients treated within the French network "FILIERE TETECOUDENT" and Reference and Competence Centres for Oral Rare Diseases were phenotyped using D4/phenodent. We used a next-generation sequencing (NGS) panel "Genodent" targeting 585 known or candidate genes in dental disorders.

RESULTS: We were able to identify the molecular defect underlying the patients' phenotypes in 17 different conditions. Interestingly, mutations in *COL17A1* were the most frequent cause of isolated AI, accounting for 8 % of all patients. Furthermore, NGS-based screening of multiple AI genes allowed us to identify a rare case of digenic inheritance in AI, with unlinked heterozygous mutations in *COL17A1* and *LAMA3* modifying the severity of the phenotype. Recognizing syndromic AI is critical for overall patient management. Orodental clinical and radiographic features of Enamel Renal Syndrome caused by *FAM20A* mutations are pathognomonic of this condition. Sequencing DNA from a seven-year-old patient presenting with isolated AI revealed a homozygous missense mutation in *CNNM4* encountered in Jalili syndrome; ophthalmological investigation subsequently confirmed this diagnosis.

CONCLUSION: Therefore, we demonstrate genetic heterogeneity in AI. Many patients were undiagnosed, suggesting that additional genes mutation have yet to be identified. Genotype/phenotype correlations might be used to reclassify the current clinical classification. The dental clinic hence serves as a gateway for the diagnosis and management of rare genetic disorders.

ACKNOWLEDGEMENTS: This work was funded through: PHRC 2008 N°4266 Amelogenesis Imperfecta, the EU-funded projects: (ERDF) A27 'Oro-dental manifestations of rare diseases, the

RMT-TMO Offensive Sciences initiative, INTERREG IV (www.genosmile.eu) and by the INTERREG V Upper Rhine program N° 1.7 “RARENET” project.

DISCUSSION:

Jennifer Kirkham (Leeds): I have been looking at information from the last Enamel conference. One of the comments there in the summary was that within the next 5 years - at the next Enamel conference - to 10 years maximum, we will have discovered all of the genes associated with AI. I wondered if you would like to comment upon how the future’s going to look, now we are here, 6 years later.

Agnès Bloch-Zupan (Strasbourg): I think this is happening and many more genes are to be discovered and each will go quickly now and even more quickly if we manage to collaborate.

#39 AMELOGENESIS IMPERFECTA: GENETIC ETIOLOGY AND DISTINGUISHING ISOLATED FROM SYNDROMIC CASES

ZHANG, H[1] and HU, JC-C[1]*

[1]Department of Biologic and Materials Sciences, University of Michigan School of Dentistry, 1210 Eisenhower Place, Ann Arbor, MI 48108

Email: janhu@umich.edu

Isolated (non-syndromic) enamel defects known as Amelogenesis Imperfecta (AI) can be autosomal dominant (caused by *ENAM*, *FAM83H, LAMA3, LAMB3, LAMC2*, *COL17A1*, or *DLX3*), autosomal recessive (caused by *ENAM, AMBN, MMP20, KLK4, SLC24A4, WDR72, ITGB6, C4orf26*), or X-linked (*AMELX*). Dominant cases caused by mutations in *LAMA3, LAMB3, LAMC2*, and *COL17A1* represent heterozygous carriers of junctional epidermolysis bullosa, a syndromic condition that is manifested when both alleles are defective. Other syndromic AI conditions, such as Immunodeficiency 9 (*ORAI1*), Immunodeficiency 10 (*STIM1*), Enamel Renal Syndrome (*FAM20A*), Cone-Rod Dystrophy and AI (*CNNM4*), and Nance Horan Syndrome (*NHS*), exhibit enamel malformations that anticipate subsequent systemic phenotypes.

OBJECTIVES: To refine exome sequence analyses of patients with inherited enamel defects of unknown etiology to a) identify mutations in proven candidate genes and b) discover new AI candidate genes/mutations.

METHOD: Genomic DNA was isolated from saliva samples collected from participating family members and characterized by whole exome sequencing. Raw sequences were aligned to a human genome assembly (GRCh37) with further deduplication. Variants were called, annotated and filtered considering allele frequency, predicted effects on protein expression and function, pedigree analysis and pattern of inheritance, etc. Known AI candidate genes were scrutinized first, then other deleterious gene variants.

RESULTS: We recruited 38 additional AI families with no obvious non-dental phenotypes. Most cases were recessive. Only rarely were variants found in the known AI candidate genes. Novel disease-causing mutations in *AMBN*; *MMP20*; and *WDR72* (3 families) were identified. Several new candidate genes are under further investigation.

CONCLUSION: Despite the large number of AI candidate genes, many more genes involved in the etiology of AI await discovery. Data sharing among separate laboratories with collections of recruited AI families will hasten the pace of discovery.

ACKNOWLEDGEMENTS: This study was supported by NIDCR/NIH grants DE-015846 and DE-012769.

DISCUSSION:

Agnès Bloch-Zupan (Strasbourg): A comment on your title of the abstract because you are distinguishing isolated from syndromic cases, what would be your thought about that?

Jan Hu (Michigan): Very briefly, in the early stage (of amelogenesis) as we know, the basement membrane component and hemi-desmosome component single allele defects will result in isolated, pitted hypoplastic AI while when there are biallelic defects of the genes, the result would be skin defects - junctional epidermolysis bullosa- plus very severe hypoplastic AI. We need to really understand the aetiology in order to be able to discern whether it is an isolated or syndromic form. In the maturation stage, as we know, many of these molecules important for ameloblast function are also utilised by other systems. For example, sodium proton exchanger, NHE (SLC9A1), is also utilised by the kidney. Therefore when this gene is mutated the result is not going be just isolated enamel defects, there would be kidney problems as well.

#40 AMELOGENESIS IMPERFECTA GENETIC INFORMATION – WHAT USE TO PATIENT CARE?

SMITH CEL[1 2], POULTER JA[2], INGLEHEARN CF[2] and MIGHELL AJ[1 2]*

[1]School of Dentistry, University of Leeds, Leeds, LS2 9LU, UK; [2]Leeds Institute of Biomedical and Clinical Sciences, St James's University Hospital, University of Leeds, LS9 7TF, UK

Email: a.j.mighell@leeds.ac.uk

OVERVIEW: Following the landmark achievement of the Human Genome Project in 2000, subsequent advances in DNA sequencing and computer sciences have revolutionised the ability to generate and interpret genetic information about individuals. How this information is used to transform human healthcare is a major challenge.

EVIDENCE: In the last 10 years, genetic discoveries arising from investigation of people with Amelogenesis Imperfecta (AI) have led to a more complete understanding of specific genetic variants with an adverse impact on amelogenesis. Some discoveries have involved candidate genes. However, genes not previously been implicated in amelogenesis have also been described, sometimes with no understanding of their function. It is clear that there are further gene variants to discover in Mendelian-inherited forms of AI.

There is improved understanding of core and more variable clinical phenotypic features for given genotypes. Hypomorphic variants add additional complexity. Advances in human teeth analyses, particularly via nano-CT, are adding further insight into the impact of specific genotypes on the resultant enamel. These ultrastructural data can be difficult to interpret within the context of other data, including that generated from animal models.

There is a drive in the UK to increase genetic testing in mainstream NHS healthcare. This change is welcomed by clinicians and offers many possibilities for improving patient care and resource use. However, the evidence-base for using AI genetic-testing to add value to care is extremely limited with respect to the impact on patients and clinicians, as well as healthcare commissioners and how patient pathways and services are organised.

CONCLUSION: Understanding of AI genetics will continue to develop, but as genetic testing is introduced to the point of care, there is a need to define through research how this will lead to effective transformation of patient care.

<u>DISCUSSION:</u>

Agnès Bloch-Zupan (Strasbourg): You're talking about genetic testing but you didn't quite discuss ethics or problems arising with the testing. Can you comment a little bit upon those?

Alan Mighell (Leeds): If it's a clinical test it's got to be a test where when you've requested the test, you are going to be able to deal with the results. One of the worries about genetic testing is, is it going to throw up something that you don't really want to know about? So the way this is being dealt with for us is, it's a test with limits, so we only test for a selective number of genes and we don't go looking for other things. This isn't research, this is about clinical care, so if it's one of the genes that's in the test panel it may just come back with a positive finding, but equally in about half of

cases it will just come back as “no mutation in any of the genes”. So that’s how I deal with that. It’s no different to having a chest X-ray if you’re a smoker, as you don’t know what you’re going to find.

Olivier Duverger (Bethesda): Most of the mutations that have been associated with AI so far are missense mutations or deletions found in the coding region of genes. Do you expect to find also a lot of mutations in regulatory regions, in intergenic and enhancer regions that we would have missed because they are more complex and is the strategy you’re using only focussing on exome sequencing or whole genome sequencing (WGS)?

Alan Mighell (Leeds): Yes, there are other more obscure mutations, you can find them. At the moment, when it comes to chairside or clinical care, this is just exome sequencing, but it (WGS) will come.

#41 FUNCTIONAL AND ESTHETIC REHABILITATION OF A PATIENT WITH ODDD SYNDROME

FRASHER, I*, PALME, S, MANHART, J

Department of Restorative Dentistry and Periodontology, Ludwig Maximilians University, Munich, Germany

Email: frasheri@dent.med.uni-muenchen.de

OVERVIEW: Etiopathogenesis**:** OculoDentoDigital Dysplasia is a rare genetic disorder due to a mutation of the *GJA1* gene on chromosome 6q 22-23. This results in misfolded connexin 43 (Cx43), a protein located in cellular hemichannels. Thus, GAP-junctions present an altered exchange of molecules. In particular, in enamel there is an abnormal accumulation of amelogenin, with a subsequent hypoplasia and reduction of thickness of enamel layer. Less than 500 cases worldwide are known.

Principal clinical aspects:

- Characteristic facial expression (microphthalmia, prominent epicanthic folds, hypoplastic alae nasi)
- Ocular manifestations: glaucoma, cataract, microcornea
- Digital anomalies: camptodactyly, clinodactyly, syndactyly
- Other comorbidities: cardiac malformations, neurologic dysfunctions
- Oral manifestations: possible partial anodontia, microdontia, enamel hypoplasia.

Dental treatment in ODDD cases has been barely reported and limited to stainless steel crowns and full coverage composite restorations.

EVIDENCE: Even if Cx43 protein has also been observed in odontoblasts[1-4], the dentin appears not to be directly involved. Histologically, only enamel seems to be involved[5]. Clinically, there is a deep bite due to microdontia and loss of vertical dimension of occlusion, consequence of abrasion and attrition (enamel chipping). Pugach et al. have analysed in animal models the shear bond strength in enamel and dentin. They concluded that dentinal bonding is more preferable than the bonding to hypoplastic or hypomature enamel[6]. Improvements in dentin bonding allow restoring the defective enamel using ceramic “table-tops” and crowns to reestablish a correct vertical dimension of occlusion and to prevent further loss of occlusal tooth structure.

CONCLUSION: Further studies need to value the correlation of mutations with a specific phenotype. Clinically, as a multisystem disorder, treatment should involve a multidisciplinary team. Considering the rapid progression of abrasion / attrition, early dental treatment, as soon as the eruption of permanent dentition is completed, with frequent follow-up is advisable.

REFERENCES: [1]Joao SM, Arana-Chavez VE. Histochem Cell Biol. 2003;119(1):21-6. doi:10.1007/s00418-002-0482-3. [2]Fried K, Mitsiadis TA, Guerrier A, Haegerstrand A, Meister B. Int J Dev Biol. 1996;40(5):985-95. [3]Muramatsu T, Hashimoto S, Shibukawa Y, Yuasa K, Furusawa M, Shimono M. Microsc Res Tech. 2013;76(10):988-91. doi:10.1002/jemt.22271. [4]Liu X, Yu L, Wang Q, Pelletier J, Fausther M, Sevigny J et al. J Dent Res. 2012;91(3):261-7. doi: 10.1177/0022034511431582; [5]Toth K, Shao Q, Lorentz R, Laird DW. J Cell Physiol.

2010;223(3):601-9. doi:10.1002/jcp.22046; [6]Pugach MK, Ozer F, Mulmadgi R, Li Y, Suggs C, Wright JT et al.. Pediatr Dent. 2014;36(5):130-6.

DISCUSSION:

Jim Simmer (Michigan): When you're doing the crown prep. into dentine, at what age do you think it is best to do that to make sure you don't get the pulps too big, so that they might get exposed?

Iris Frasher (Munich): We started this treatment as provisional treatment, so the idea was to wait until the patient was 18 or 20 years old for the definitive treatment. However, there was an urgent need because she had sensitivity in this area, so we had to do some kind of filling, but the filling was always chipping because the condition of the enamel was not so good and also because of progression of erosion of the occlusal surface under occlusal forces. We would suggest to do it as soon as all the permanent teeth have erupted because we know that it will progress towards caries and it will go towards chipping, so why wait until there is a problem or a joint problem?

Jim Simmer (Michigan): How old was this patient?

Iris Frasher (Munich): She's now 15 years old, she went through 6 months of bites for elevation of the occlusal dimension and now we're sure that there were no joint problems.

#42 CRYSTALLOGRAPHY AND MICROSTRUCTURE OF ENAMEL AFFECTED BY HYPOMATURATION AMELOGENESIS IMPERFECTA

SIDDIQUI, S[1] and AL-JAWAD, M[2]*

[1]Interdisciplinary Nanoscience Center (iNANO), Building 1590, Gustav Wieds Vej 14, Aarhus 8000, Denmark; [2]Dental Physical Sciences Unit, Institute of Dentistry, Barts and The London School of Medicine and Dentistry, London, E1 4NS

Email: m.al-jawad@qmul.ac.uk

OBJECTIVES: To characterise the spatial distribution of crystallographic preferred orientation and mineral concentration in human dental enamel clinically diagnosed with pathological hypomaturation Amelogenesis Imperfecta (AI). Understanding structural defects that occur during enamel maturation can give insights into principles underlying normal enamel biomineralisation, alongside informing clinical treatments.

METHOD: Intact human maxillary premolars, two clinically diagnosed with hypomaturation AI, one type-matched sound sample were used. Whole tooth samples were scanned using x-ray microtomography (XMT), then sectioned into 60 µm slices for 2D synchrotron x-ray diffraction (2D-SXRD) at the XMaS beamline, ESRF, and SEM imaging.

RESULTS: From XMT, hypomature enamel exhibited lower mineral concentration at the crown surface with a low-mineral density (1.7 $g.cm^{-3}$) uniform layer approximately 0.8 mm thick, present around the entire enamel surface, forming a halo-like outer layer. In contrast the healthy enamel had close to uniform mineral density distribution. From 2D-SXRD, texture distribution maps of the two AI hypomature samples showed low texture values in the cusps, outer surfaces, cervical regions and fissure, forming the same band-like appearance. In contrast, the control sample had highest texture in cuspal regions, with texture decreasing towards the EDJ and cervical regions. SEM images showed short, thick, tablet-like structures at the surface of the hypomature enamel, whereas a more prismatic, organised structure was observed in the bulk, with a distinct sharp boundary between these two morphologies.

CONCLUSION: The hypomature AI enamel had disruption in mineral concentration, crystallite organisation and microstructure predominantly in a surface layer ~0.8 mm thick. Additionally, on average, the texture distribution was low throughout the whole crown as compared to healthy enamel. This suggests that hypomaturation AI affects the nano- and subnano-scale structures of the outer surface most severely, but also affects the bulk enamel, an important finding for the understanding of spatially and temporal progression of enamel maturation.

DISCUSSION:

Agnès Bloch-Zupan (Strasbourg): So you mentioned that these teeth and the patient that they are coming from do not have a genetic diagnosis. Do you think this microscopic approach to phenotyping could help us understand, or could lead to, good genotype/phenotype correlation at this microscopic scale?

Maisoon Al-Jawad (QMU): We have published work previously where we've known the genetic underlying mutation, in *ENAM* in particular. I think the power lies in combining these two, in both knowing the genotype and combining that with the careful understanding of the physical phenotype and how that manifests as clearly there's a question of severity as well that you can assess with these techniques, that you can't assess just by knowing the genetic mutation.

#43 NOVEL *KLK4* VARIANT ASSOCIATED WITH ENAMEL HYPOMINERALISATION AND STRUCTURAL ABNORMALITIES

SMITH, CEL[1,2*], BROOKES, SJ[1], DAY PF[3], POULTER JA[2], INGLEHEARN, CF[2], MIGHELL, AJ[2,3] and KIRKHAM, J[1].

[1]Department of Oral Biology, School of Dentistry, St James's University Hospital, University of Leeds, Leeds, UK. LS9 7TF; [2]Section of Ophthalmology and Neuroscience, St James's University Hospital, University of Leeds, Leeds, UK. LS9 7TF; [3] School of Dentistry, University of Leeds, Leeds, UK. LS2 9LU

Email: c.e.l.smith@leeds.ac.uk

OBJECTIVES: To characterise the genotype and enamel phenotype in a case of hypomaturation type amelogenesis imperfecta (AI).

METHODS: DNA from a patient with hypomaturation type AI was subjected to whole exome sequencing (WES). Micro-computerised X-ray tomography (µCT), scanning electron microscopy (SEM), microhardness testing (MH) and energy-dispersive X-ray spectroscopy (EDX) were used to characterise the enamel phenotype for deciduous teeth from one individual. Results were compared to matched controls.

RESULTS: WES revealed the presence of a previously unreported homozygous frameshift variant in kallikrein related peptidase 4 (*KLK4*), c.632delT, p.(L211Rfs*37). The mutant transcript is predicted to escape nonsense-mediated decay and the catalytic triad of residues, essential to the function of all KLKs, remains present. For affected teeth, the majority of the crown was covered by enamel, but enamel was absent in localised areas. Analysis by µCT and MH revealed that affected enamel appeared to consist of two layers of distinct mineral density and hardness; the outer enamel was of a similar density and hardness to that of control enamel, but the inner enamel was hypomineralised and was much softer. SEM showed there to be a clear demarcation line between inner and outer enamel. EDX showed that the inner enamel layer contained elevated nitrogen and carbon and less calcium and phosphorus compared with control teeth, suggesting the presence of organic material within the tissue.

CONCLUSION: This report details the fourth *KLK4* variant to be identified in AI patients. The finding of a structurally distinct, demarcation line between outer and inner enamel is surprising. Analysis of the phenotype suggests that loss of enamel may be associated with the mineralisation pattern observed and the effect of this on enamel mechanical properties.

DISCUSSION:

Jim Simmer (Michigan): Did you examine the patient to see if there were any other systemic phenotypes besides the dental condition?

Claire Smith (Leeds): Alan may be better placed to answer this.

Alan Mighell (Leeds): The patient wasn't formally assessed but there were no obvious, recognised associated illnesses, but we didn't go back and formally go over them again. I think it is a point that is very important with all these patients.

Claire Smith (Leeds): Can I ask Jim why you asked that?

Jim Simmer (Michigan): It's the same for us, we've never seen any non-dental conditions in the *KLK4* patients or mice.

Henry Margolis (Forsyth): Do you have independent evidence that the mutated KLK4 enzyme cleaves enamel proteins?

Claire Smith (Leeds): I have not yet modelled the mutation, these are just the preliminary findings, so no I don't.

Agnès Bloch-Zupan (Strasbourg): Do you have an idea why there is such a clear cut between the two different layers at the ultra-structural level?

Claire Smith (Leeds): So because this is softer it may be that when it's acid etched and prepared (for SEM) maybe it's worn away more. There could also be residual action of any MMP20 (in the outer enamel) as the ameloblasts are up there and any more endocytosis in this layer might explain the observation. Finally, if there's any KLK4 activity at all, then any KLK4 that does make it, would be found more near the surface.

Alex Vieira (Pittsburgh): I haven't seen work done at this level for enamel, it's so elegant and nice. I've seen it in dentine. In dentine diseases, normal dentine becomes disorganised, so this feels exactly the same to me.

#44 *MMP20* MUTATIONS CAUSING ENAMEL HYPOMATURATION IN TWO FAMILIES

KIM, YJ[1], KANG, J[1], SEYMEN, F[2], KORUYUCU, M[2], GENCAY, K[2], HU, JC[3], SIMMER, JP[3], KIM, J-W[1 4]

[1]Department of Molecular Genetics & Dental Research Institute, School of Dentistry, Seoul National University, Seoul, Korea; [2]Department of Pedodontics, Faculty of Dentistry, Istanbul University, Istanbul, Turkey; [3]Department of Biologic and Materials Sciences, University of Michigan, Ann Arbor, MI, USA; [4]Department of Pediatric Dentistry & Dental Research Institute, School of Dentistry, Seoul National University, Seoul, Korea

Email: pedoman@snu.ac.kr

OBJECTIVES: Amelogenesis Imperfecta is a collection of rare inherited disorders that affect tooth enamel formation, in quantity and/or in quality. The aim of this study was to identify the genetic etiologies of two families presenting with hypomaturation Amelogenesis Imperfecta.

METHODS: DNA was isolated from peripheral blood samples of participating family members. Whole exome sequencing was performed using DNA samples from the two probands. Sequencing data was aligned to the NCBI human reference genome (NCBI build 37.2, hg19) and sequence variations were annotated with the dbSNP build 138.

RESULTS: Mutations in *MMP20* gene were identified in both probands. A homozygous missense mutation (c.678T>A; p.His226Gln) was identified in the consanguineous Family 1. Compound heterozygous *MMP20* mutations (c.540T>A, p.Tyr180* and c.389C>T, p.Thr130Ile) were identified in the non-consanguineous Family 2. Affected persons in the family 1 showed hypomaturation AI with dark brown discoloration, which is similar to the clinical phenotype in a previous report with the same mutation. However, the dentition of the proband in the family 2 exhibited slight yellowish discoloration with reduced transparency.

CONCLUSION: These results expand the mutational spectrum of the *MMP20* and broaden our understanding of genotype-phenotype correlations in Amelogenesis Imperfecta.

DISCUSSION:

Agnès Bloch-Zupan (Strasbourg): You're relating the severity of the phenotype to the remaining possible activity of the enzyme. Do you think that kind of event could explain other variability in the phenotype for other types of AI?

Jung-Wook Kim (Seoul): Yes I think so. All the mutations and assays there are bad mutations / mild mutations in my patients, but within the specific type of the AI we can get some kind of genotype phenotype correlation.

Agnès Bloch-Zupan (Strasbourg): It's probably a lot of work to do in that field to better understand the disease.

#45 FAM20A GENE MUTATION: AMELOGENESIS OR ECTOPIC MINERALIZATION?

LIGNON, G[1]*, BERES, F[1], ROUZIÈRE, S[2], MOUTON, L[3], DE LA DURE-MOLLA, M[4], DESSOMBZ, A[1] and BERDAL, A[1 4]

[1]Molecular Oral Pathophysiology, Cordeliers Research Center, UMRS 1138 INSERM. Paris-Descartes, Pierre-et-Marie-Curie, Paris-Diderot Universities, 15 rue de l'Ecole de Médecine, 75006 Paris, France ; [2]Laboratoire de Physique des Solides. UMR 8502 CNRS. Paris-Sud University, 1 rue Nicolas Appert, 91405 Orsay CEDEX, France ; [3]ITODYS, UMR 7086 CNRS. Paris-Diderot University, Sorbonne Paris Cité, 15 rue Jean-Antoine de Baïf, 75205 Paris CEDEX France ; [4]Reference Center of Rare Buccal and Facial Malformations MAFACE-Rothschild Hospital, APHP, Paris, France

Email: guilhem.lignon@crc.jussieu.fr

BACKGROUND AND OBJECTIVE: *FAM20A* gene mutations result in Amelogenesis Imperfecta (AI) and associated ectopic mineralization in soft tissues (enamel renal syndrome ERS OMIM# 204690). Here, we characterized the tissue covering coronal dentin in affected patients carrying 8 different mutations.

METHODS: Clinical and genetic data, radiographs and unerupted teeth from healthy donors and ERS patients were collected under ethical and regulatory rules. Sections were analyzed by scanning electron microscopy (SEM), energy dispersive spectroscopy (EDS), X-ray diffraction and X-ray fluorescence (XRF).

RESULTS: Clinical and radiographical appearance of affected enamel varied depending on the *FAM20A* gene mutation and among patients, individual teeth, albeit with a systematic reduction in thickness. Prisms were rarely observed with SEM and restricted to the inner most zones. The bulk of the mineralized material covering the crown was formed by layers with changing electron-densities, organized into lamellae and micronodules. Here, XRD evidenced a crystalline structure with loss of anisotropy (vs. sound enamel) and XRF revealed some compositional alterations. Porosity progressively increased at the periphery ending with loose nanonodules adjoining the enamel organ. Enamel organ and all soft dental tissues contained ectopic nano/micronodules with a similar organization.

CONCLUSION: The structure covering dentin in all ERS patients (except true enamel at the dentinoenamel junction) displayed an ultrastructural pattern reminiscent of ectopic mineralization, evidenced here in the gingiva, periodontal ligament and enamel organ. This study, and previous data in KO mice, suggests that the bulk of ERS tissue covering coronal dentin is the result of ectopic mineralization. *In vitro* studies have shown that FAM20A is able to phosphorylate several mineralization regulators that might inhibit mineralization in soft tissues while stimulating mineralized tissue formation. Genotype/phenotype correlations and *in vitro* modelling with human cells should delineate this putative dual role for FAM20A in ERS.

DISCUSSION:

Agnès Bloch-Zupan (Strasbourg): If I understand correctly, you are saying that these calcified structures are actually not so much enamel, but a sign of this ectopic mineralisation process. Did you analyse some of the other ectopic calcification in other locations, do they look alike?

Guillaume Lignon (Paris): We analysed the patient for ectopic mineralisation in the enamel organ but we have not yet found the property of this one.

Agnès Bloch-Zupan (Strasbourg): And can you comment on why the teeth are not erupting in these diseases, do we have ideas?

Guillaume Lignon (Paris): No.

#46 ER STRESS AS AN AETIOLOGICAL FACTOR IN AMELOGENESIS IMPERFECTA

BROOKES, SJ[1]*, BARRON, MJ[2], SMITH, CEL[3], POULTER, JA[3], INGLEHEARN, CF[3], MIGHELL, AJ[1], DIXON, MJ[2] AND KIRKHAM, J[1]

[1]School of Dentistry, University of Leeds, Leeds LS2 9LU, UK; [2]Faculty of Life Sciences and School of Dentistry, Manchester Academic Health Sciences Centre, University of Manchester, Michael Smith Building, Oxford Road, Manchester M13 9PT, UK; [3]Leeds Institute of Biomedical and Clinical Sciences, St James's University Hospital, University of Leeds, Leeds LS9 7TF, UK

Email: s.j.brookes@leeds.ac.uk

OVERVIEW: Amelogenesis imperfecta (AI) has been attributed to secretion of dysfunctional enamel proteins affecting extracellular nucleation, organisation and modulation of enamel crystal growth. However, a number of human diseases (proteopathies) are caused by the intracellular aggregation of mutated proteins in the secretory pathway affecting cell function via ER stress and the unfolded protein response (UPR) - a complex interlinked set of intracellular signalling pathways that attempt to ameliorate ER stress but, *in extremis*, trigger apoptosis. We hypothesise that some examples of AI are proteopathies.

EVIDENCE: Mice heterozygous for p.Y64H *Amelx* and p.S55I *Enam* mutations exhibited intracellular characteristics of a classic ER stress response and UPR activation. Phenotypically, these mutations were associated with a structurally normal initial enamel layer and an abnormal outer enamel layer. A similar phenotype was also seen in a human heterozygous p.L31R *ENAM* mutation. We suggest that this is due to the UPR initially operating in pro-survival mode allowing ameloblasts to function normally. With chronic stress, the UPR reduces protein synthesis and ultimately triggers ameloblast apoptosis. Furthermore, the UPR inhibitor 4-phenylbutyrate rescued the heterozygous p.Y64H *Amelx* phenotype suggesting that pharmacological intervention may rescue proteopathic AI. Ameloblasts in *Fam20a*$^{-/-}$ mice also exhibited UPR activation but no structurally normal enamel was produced in these animals.

CONCLUSION: We hypothesise that AI can be driven by the UPR operating in ameloblasts. However, the enamel matrix may still support enamel production, providing the UPR can be manipulated to promote ameloblast survival. Further work, using *in vitro* ameloblast-like cell models expressing specific mutated proteins associated with AI, will screen for therapeutics that favourably modulate the UPR and thus identify which AIs may be amenable to intervention. Finally, we note that the UPR is activated by other factors such as fluoride-induced oxidative stress, indicating a commonality between some inherited and non-inherited enamel disorders.

DISCUSSION:

Jim Simmer (Michigan): For the condition where you were showing the recovery of the phenotype, what was the genotype of that mouse, was it a male?

Steve Brookes (Leeds): The heterozygous female.

Jim Simmer (Michigan): If it’s a heterozygous female, because of the lyonisation, how would you know what it would look like without the treatment, because you can get heterozygous females that have completely normal looking enamel just by random.

Steve Brookes (Leeds): Every heterozygous female that we saw was affected. There wasn't one that didn't exhibit some phenotype or other.

Jim Simmer (Michigan): You never saw heterozygous female mice that appeared to be unaffected? That should be random because when we are looking at the *Amelx* null mouse we find heterozygous females where the enamel can go right through to the normal range.

PANEL DISCUSSION:

Alex Vieira (Pittsburgh): I have a comment and a question for anybody who wants to reply. The comment is - and I appreciate that a couple of you mentioned that you are very optimistic that you are going to have identified a 100% of the mutations for amelogenesis imperfecta (AI) in a few years - I'm more sceptical than you are. Just based on looking at other diseases that I'm familiar with, I think there are a number of challenges. This question relates to a couple of those. One is, there are genes that are yet to be found, and even though you have the ability to screen for all of those, it is still quite hard to tease out that information from the bioinformatic data. Even if you manage that, I think there are things that are in sequences that are not coding for proteins and then we don't have perfect models or perfect cells to tease those out. Even if you can sequence the whole genome, you still have to work on a gene by gene level. So honestly, I believe that, not even in my lifetime are we are going to see all of the mutations described. That's just one general comment but the question is a relevant one. I am with you (agree that) in relation to your concerns about "syndromic" versus "non-syndromic" and the need to move to a molecular classification. Do you feel that's the direction we should go towards, particularly for (those of) you interested in genetic counselling for example? Should we stop using (general terminology) such as syndromic / isolated etc and be more specific in characterising diseases such as AI that's associated with kidney versus AI that is not associated and then it looks more severe or less severe based on what the mutation is etc? Should we use that language instead of just talking about all the syndromes that we deal with? To call it syndromic / non-syndromic is not super helpful when it comes back to the families for example. To be more precise in our descriptions, is that the direction that we should be going in?

Alan Mighell (Leeds): Thank you – we certainly will not have all the mutations (discovered and described) because obviously all the mutations will just go on and on, but I think we'll have a pretty good idea of which genes are affected when you're talking about a single gene condition; it depends how you define success. It's never going to be 100% of all mutations and that's for sure. Of course, we are making the assumption here that AI as we recognise it at the moment, is (associated with) single genes (monogenetic). For sure over time, we'll start to recognise more complex patterns as well. I don't know if that clarifies the issue, but certainly we now have the technology to be able to really crack on through the rest of the single genes, it's the will to do that collectively that will determine how quickly we reach the point, in my opinion. In terms of your second question, I don't worry about "syndromic" versus "non-syndromic". These are just descriptive terms. We now have really quite good insight into how you can have a variant at this point in the gene and get a completely different phenotype than if you had it at a different place. "Syndromic" versus "non-syndromic" suited us at the time but we will move away from that, I suspect, as time goes on.

Agnès Bloch-Zupan (Strasbourg): Just a few comments to the same question. There are other levels of regulation that are not genetic and that we will not tackle with those approaches, that's for sure. On the syndromic / non-syndromic issue, it's not a question of wording or semantics, it's for the patient. You have to look for all the signs, you have to make sure that the AI is not associated to other symptoms, because if it is, it changes patients' overall health management, that's what my comment is on that.

Jim Simmer (Michigan): Can I also ask about this same issue? I see that for this advance where we're getting all these mutations defined, that as that turns into normal clinical practice, then the people that have these patients are going to be observing other phenotypes and then we're going to learn what other phenotypes are possible. At the current time we have *WDR72, SLC24A4* and *FAM83H* which are three genes that we know are selectively preserved even when teeth have been lost during evolution, so we know that there's other things that those genes are being selected for. We therefore know that we are missing systemic conditions inside these patients and I think that once this just simply advances to be a clinical thing, that these things will be known and that's all going to be so much better for everybody because of that advance, so I think it's important that we are going to be learning more about syndromic conditions; that we are going to learn about what else comes with this from the advances that you guys are describing.

Jan Hu (Michigan): This is just a comment for Alex's question. In terms of how many more years, whether in our lifetime we will be able to really fully solve the complex collection of genes that result in AI. I believe that primarily, we are all using exome sequencing at this time, focusing on these single genes for AI. However, outside of the enamel field, in the basic developmental biology field, enhancers, suppressors and other genetic informations are being rapidly decoded and mapped by whole genome sequencing. I think as technology continues to advance, we will be able to move into that area quickly and hopefully there will be relevant information for us to know as well, in terms of the enhancers and the suppressors of these critical genes for AI and that would facilitate our discovery toward solving the remainder of cases.

Kseniya Shuturminska (QMU): I was wondering in terms of the material side of enamel, when the enamel is affected, you said we lose the orientation of the crystals but no one really mentioned about the size of the crystals, do they stay the same? You know in sound enamel that the crystal sizes are very well defined, does it change when it's affected by AI?

Maisoon Al-Jawad (QMU): Certainly we see differences in the anisotropy, so the shape of crystals is different (in AI). We haven't seen any evidence that the thickness in cross section is different. That looks pretty similar, at least under the SEM observations that we've done, but certainly the (in the case of the) length, you get disruption in that; that continual length and that needle-like shape. So, in terms of dimensions, they are slightly different and you can measure that either qualitatively with the microscopy imagining or you can apply some models to anisotropic particle sizes with diffraction data so you can observe that.

Tom Diekwisch (Dallas): Steve, I was very impressed with your rescue studies and I'm just wondering if ER stress seems to be in all of these situations, perhaps something that certainly contributes to the phenotypes. I'm wondering whether you have considered any sort of gene therapy with your drug (4-phenylbutyrate) or some alternative because this ER seems to be very sensitive.

Steve Brookes (Leeds): That's true; I think the ER is uniquely sensitive to any sort of stress but wonder whether gene therapy would ever be economically viable for AI, especially if you've got such a cheap drug option as phenylbutyrate. I mean, it's cheaper than aspirin!

Sherif Elsharkawy (QMU): I have a question for Dr Hu and everyone, regarding the mutations for AI. Usually we look at single mutations right, so it's just one protein that's affected, but we don't really know how this protein, when affected, is going to affect other proteins, so we start to mis-understand some processes that could be important. How can we define these protein-protein interactions and how can we get rid of any confusion? This is one thing, the other thing is that, would we be able to, at some stage, to save the permanent teeth with gene therapy with better understanding, keeping in mind this protein-protein interaction issue as well, would we be able to save the permanent teeth?

Jan Hu (Michigan): I think that's a very good question that you asked. The current limitation is that we are not able to get the developing teeth from the affected (human) individual and conduct studies while the majority of the mouse models that we generated are essentially gene absence models. So it doesn't really allow us to understand in cases where a mutant protein is present how it interacts and affects other proteins that are critical for enamel formation. In my opinion, I feel that the next important step for the field is really to generate mutation-specific models like Tomas Wald presented earlier and it is with that type of model that would allow us to see the protein-protein interactions and the subsequent pathology, as well as to devise intervention strategies.

Agnès Bloch-Zupan (Strasbourg): Just a comment about gene therapy. Why do you do gene or diagnostic testing? You do that for diagnosis, for early pregnancy termination in case of very rare and very severe diseases, you do that for genetic counselling for the same kind of issues and for the moment I do not think AI as a, let's say, a lone manifestation, would fit in the criteria for this kind of issues and investigations.

Jan Hu (Michigan): And to follow Agnès's comment, we can also learn from other conditions such as cystic fibrosis and understanding the different classification of a disease pathology. There are different strategies to correct or potentiate mutant protein functions, instead of totally replacing the defective gene. There are small molecules that can be used to potentiate the secretion of a mutant protein, or to correct partial function of the mutant proteins, but the key is to understand exactly which gene is mutated and how does the mutation impact the cell function. It is through the understanding of the pathology that we will be able to utilise the already known approaches to manage other genetic disorders and utilise those strategies to correct the problem.

Mike Hubbard (Melbourne): The literature has very widely varying suggestions of the prevalence of AI so I'm wondering whether the panel has any updates? Dr Kim, do you have an idea of prevalence in Korea? For Steve Brookes, the chemical rescue I agree is fantastic. Have you got an idea of what percentage of cases might be amenable to that approach? Could there be other rescues besides the phenylbutyrate? So, prevalence? 1 in 14,000 or 1 in 700 is kind of a big range!

Jung-Wook Kim (Seoul): Actually we don't have such specific data but based on my short experience I think it's about 1 in 10,000.

Mike Hubbard (Melbourne): That's an easy number to remember!

Alan Mighell (Leeds): I will reiterate the conversation we had earlier, which is I don't think it (quoted AI prevalence) really matters too much. What I know is that there's plenty of it around and although we keep working with the same population, there's just more and more families coming out so it's not a rare condition in the true sense of some conditions that, for example, Agnès may deal with, that are truly rare. "Straightforward" AI, for want of a better word, isn't that uncommon and it does very much depend where you draw the line as to what is AI and what is another type of developmental enamel defect.

Jan Hu (Michigan): I would like to corroborate what Alan said. We really need to consider all the (AI) subtypes together and when we look at them aggregated, I feel that the frequency is not less than dentinogenesis imperfecta, which is set at about 1 in 8,000, and so I think Jung-Wook's number is very close if we look at all the subtypes together.

Agnès Bloch-Zupan (Strasbourg): Just one similar comment. If I look at the patients from the rare disease reference centre, I have many more patients with AI than I have with dentinogenesis imperfecta, so I think it's actually the other way round.

Jung-Wook Kim (Seoul): We had the debate about MIH and whether it is influenced by genetic factors or environmental factors. Maybe if Alex (Vieira) can find such kind of genetic influence, MIH should be included in the AI category. If so, then maybe the prevalence will be much higher than we think now.

Mike Hubbard (Melbourne): Returning to the misfolded percentage question Steve?

Steve Brookes (Leeds): I've forgotten what the question was now! "Does this work in every case," was that the question?

Mike Hubbard (Melbourne): You wouldn't suggest that the pathology is due to protein mis-folding in every case, so have we got an idea of what percentage of AI cases are due to mis-folding?

Steve Brookes (Leeds): The answer to that is, I don't know. There seems very little point in restoring the secretory pathway of a mutated protein that's still not functional once it hits the matrix, so rescuing the secretory pathway in that case probably wouldn't do much good. In p.Y64H *Amelx* mice, it's just a case of keeping the affected ameloblasts alive, so they're not contributing to the enamel, but neither are they having any detrimental effect. You just keep them alive, keeping the ameloblasts there intact, until the programme is finished. If you restore the secretory pathway of an affected protein but it's still non-functional in the matrix, then it's not going to rescue the phenotype.

Mike Hubbard (Melbourne): I guess I'm leading to the question, because phenylbutyrate is so well clinically-trialled, are there people out there that a clinical trial could be done on now?

Steve Brookes (Leeds): I mean in this p.Y64H *Amelx* mouse model, the phenylbutyrate doesn't restore the secretory pathway at all. It's just anti-apoptotic. In this sense, it's not acting as a chaperone, or restoring secretion.

Mike Hubbard (Melbourne): Thank you for the clarification

Tom Diekwisch (Dallas): I was impressed by your connexin phenotyping. I think that it gives us an idea of how much these non-classical enamel proteins also can contribute to AI. What do those connexins do in amelogenesis, or what does yours (Cx43) do particularly?

Iris Frasher (Munich): Actually this was the main question when we had the genetic results, at what level is this connexin influencing, is it just enamel or dentine? Actually it was found that it is both in enamel and in dentine, but in enamel there is this kind of disruption of the organisation of the ameloblasts and there is accumulation of amelogenin in a disorganised way, whereas in dentine there is no alteration. The dentine is thicker in these cases because of the production of secondary dentine because of occlusal forces, there is more production of dentine, this is what has been found. What we do not know yet is what's actually the pathogenesis, what is the whole pathway, how does this connexin influence? Is it a wrong passage of amelogenin among the cells or are there ions involved, we do not know yet.

Tom Diekwisch (Dallas): Fascinating, thank you.

Agnès Bloch-Zupan (Strasbourg): We know that dentine and enamel development are so intermingled, so AI patients have dentine anomalies and DI patients have enamel anomalies so these are also things to look at.

Ariane Berdal (Paris): Yes. Steve, I would just like to point to the papers that you have generated and what was interesting in your data was the differences on the poster of Jim Simmer with electron microscopy in these new mutants for enamel peptides. So, the first layer (of enamel) is normal and then you have a problem and when you look at mice (because in humans it's difficult to do this) you can often see separation of the (cell) polarity. Do you think that there is a relationship between the process of a particular model of stress and the loss of polarity because that is really present in many different situations? Would you link the processes?

Steve Brookes (Leeds): Yes, the effect you get in the enamel organ and the ameloblast layer is that it is very difficult to see where the polarity is. The cells are so disturbed, and the monolayer is so disturbed, that you can't really see cell polarity anymore. It looks as if someone has just gone in there

and scrambled the enamel organ basically, so it's difficult to say if the polarity is being affected because you cannot recognise polarity anymore at the histological level.

Mohammed Al-Mosawi (QMU): My question is to Dr Brookes. You mentioned that one of the treatments will inhibit apoptosis. What will be the fate of the ameloblasts if they do not undergo apoptosis? Can this be controlled so as to inhibit apoptosis at certain times and what will be the fate of the ameloblasts?

Steve Brookes (Leeds): All ameloblasts will undergo apoptosis eventually. Are you asking whether the drug will stop that natural fate?

Mohammed Al-Mosawi (QMU): Yes. So the ameloblasts won't die eventually?

Steve Brookes (Leeds): I think eventually, if they erupt out, they will die if the blood supply is cut off as the enamel organ goes. As the tooth erupts, the vascularity will go and it will just erupt out and they will still die in that sense. Certainly when we treated with phenylbutyrate, the enamel organs seemed to look normal, almost like a control enamel organ, so there's nothing that is saying that these ameloblasts are going to persist and stay alive forever and erupt out, we didn't see that.

Agnès Bloch-Zupan (Strasbourg): I would like to thank all the contributors for their action in the field and as you realise, there are more questions than answers and I look forward to the years to come, thank you.

Session 7: Animal Models

Moderator: John Bartlett, Ohio State University, USA

#47 ENAMEL NANOHARDNESS IN WILD-TYPE, *AMELX*, *WDR72*, *FAM83H*, AND *KLK4* MICE

HU, Y*, DONNELLY, LA-J, ZENG, C, SIMMER, JP and HU, JC-C

#48 INTRAVESICULAR PHOSPHATASE PHOSPHO1 FUNCTION IN ENAMEL MINERALIZATION AND PRISM FORMATION

ROSENE, L*, PANDYA, M, FOSTER, B, MILLÁN, JL, and DIEKWISCH, TGH

#49 ENAMEL DEFECTS IN *FAM83H* TRUNCATION KNOCK-IN MICE

WANG, S-K*, HU, Y, SMITH, CE, ZENG, C, HU, JC-C and SIMMER, JP

#50 ENAMEL STRUCTURE IN A NOVEL *FAM20A* MUTATION RECAPITULATES *Fam20a*$^{-/-}$ PHENOTYPE

KIRKHAM, J*, MIGHELL, AJ, SMITH, CEL, POULTER JA, INGLEHEARN, CF, DIXON, MJ, BARRON, MJ, KLETA, R, HIMMERKUS, N, BLEICH, M, AL-BAHLANI, S and BROOKES, SJ

#51 DOSE-DEPENDENT RESCUE OF KO AMELOGENIN ENAMEL BY TRANSGENES *IN VIVO*

XIA, Y, BIDLACK, F, PUGACH, MK*

#52 MMP20 OVEREXPRESSION DISRUPTS AMELOBLAST CELL POLARITY

SHIN, M and BARTLETT, JD*

#53 AMELOBLASTIN OVEREXPRESSION IS LINKED TO ENAMEL HYPOMINERALIZATION

CHUN, YP*

#54 ROLE OF TIGHT JUNCTION CLAUDINS DURING AMELOGENESIS

BARDET, C*, WU, Y, DIALLO, MT, RIBES, S, FIGUERES, ML, BREIDERHOFF, T, MULLER, D, HOUILLIER, P and CHAUSSAIN, C

#47 ENAMEL NANOHARDNESS IN WILD-TYPE, *AMELX*, *WDR72*, *FAM83H*, AND *KLK4* MICE

HU, Y[1]*, DONNELLY, LA-J[1], ZENG, C[1], SIMMER, JP[1] and HU, JC-C[1]

[1]Department of Biologic and Materials Sciences, University of Michigan School of Dentistry, 1210 Eisenhower Place, Ann Arbor, MI 48108

Email: yyhu@umich.edu

AMELX, *WDR72*, *FAM83H*, and *KLK4* mutations cause Amelogenesis Imperfecta in humans.

OBJECTIVES: to measure and compare the enamel nanohardnesses of wild-type, *Amelx*, *Wdr72*, *Fam83h* and *Klk4* null or knockin mice that phenocopy enamel defects in humans.

METHODS: Left and right hemi-mandibles (at 7 weeks) were dissected free of soft tissue, dehydrated, embedded in epoxy, cut transversely at the level of the labial alveolar crest, and re-embedded in Castolite AC. The incisor cross-sections were polished and nanohardness-tested at spaced intervals, with separate measurements obtained for the inner, middle, and outer enamel when the enamel layer was sufficiently thick to allow it. Nanohardness testing was performed using a Hysitron 950 Triboindenter with a nanoDMA transducer and Berkovich probe. The nano-indentations were analyzed using the Triboscan 9 software.

RESULTS: The average nanohardness values for $Amelx^{+/+}$ $Amelx^{+/-}$, and $Amelx^{-/-}$ enamel were 3.63 ± 0.75, 3.46 ± 0.91, and 1.61 ± 0.80 gigaPascal (GPa), respectively. The *Amelx* null enamel was softer away from the cervical margins (1.22 ± 0.59 GPa). Thus the overall $Amelx^{-/-}$ enamel hardness score was only half that of the wild-type, while the hardness score of the enamel away from the cervical margins was only one third that of the wild-type. The enamel nanohardness were measured in similar detail in all genotypes and sorted in the following order: [highest] Wild-type > *Fam83h* > *Amelx* > *Klk4* > *Wdr72* [lowest].

CONCLUSION: The nanohardness of wild-type enamel is not uniform throughout, but is hardest in the outer enamel, near the surface. The enamel is particularly soft in the *Klk4* and *Wdr72* null mice, which highlights the importance of matrix degradation and resorption, respectively, for maturation of the enamel layer. Nanohardness testing detected even normal variations in wild-type enamel and is a sensitive means of detecting subtle differences between samples. This study was supported by NIDCR/NIH grants DE-015846 and DE-012769.

DISCUSSION:

John Bartlett (Ohio): What makes the enamel harder at the outer surface in the *Klk4* ablated mice?

Jan Hu (Michigan): Ameloblasts likely endocytose enamel matrix proteins, such as amelogenin, that are near the enamel surface. This occurs for cleaved enamel matrix proteins when KLK4 is present.

John Bartlett (Ohio): So, in the *Klk4* ablated mice, the bigger, uncleaved proteins from the deep enamel cannot rise to the surface to be endocytosed?

Jan Hu (Michigan): Yes.

#48 INTRAVESICULAR PHOSPHATASE PHOSPHO1 FUNCTION IN ENAMEL MINERALIZATION AND PRISM FORMATION

ROSENE, L[1,2]*, PANDYA, M[1,2], FOSTER, B[3], MILLÁN, JL[4], and DIEKWISCH, TGH[1,2]

[1,2]Department of Periodontics and Center for Craniofacial Research and Diagnosis, Texas A&M College of Dentistry, 3302 Gaston Avenue, Dallas, TX 75206, USA; [3]Division of Biosciences, Ohio State University College of Dentistry, 305 W 12th Avenue, 4163 Postle Hall, Columbus, OH 43210, USA; [4]Sanford Children's Health Research Center, Sanford-Burnham Medical Research Institute, 10901 N Torrey Pines Rd, La Jolla, CA 92037, La Jolla, CA, USA

E-mail: diekwisch@tamhsc.edu

Amelogenesis relies on the transport of large amounts of mineral ions from the adjacent blood vessels to the developing enamel layer. It has long been thought that in ameloblasts, the transport and early enamel biomineralization occurs without the involvement of matrix vesicles. In previous studies, PHOSPHO1 has been identified as a matrix vesicle phosphatase that releases phosphate from membrane-associated phosphocholine and phosphoethanolamine during the early mineralization of bone, dentin, and cementum.

OBJECTIVES: We seek to determine the function of PHOSPHO1 in amelogenesis.

METHODS: PHOSPHO1 function during amelogenesis was compared between *Phospho1*$^{-/-}$ mutant mice and wild-type controls. Fourteen days old, same stage first mandibular molars were formalin fixed and compared between both groups under identical conditions using immunohistochemistry, X-ray analysis and micro-computed tomography. For scanning electron microscopy and elemental analysis, samples were dehydrated and sectioned in sagittal direction.

RESULTS: Immunoreactions demonstrated PHOSPHO1 staining in wild-type ameloblasts, while mutant mice revealed densely stained deposits in the stratum intermedium. Wild-type EDX elemental analysis revealed a significant 7.3 % reduction in phosphate content in *Phospho1*$^{-/-}$ mutant mice when compared to wild-type controls ($p < 0.05$) while the difference in calcium was not statistically significant. Scanning electron microscopy of EDTA-etched enamel surface revealed classic picket fence patterns in wild-type controls, while the prism structure of mutant mice was obscured, in part because of a 1.56 fold increase in enamel prism width in *Phospho1*$^{-/-}$ mutant mice ($p < 0.0001$). Moreover, individual prisms were less separated from each other as a result of a 45.3 % reduced mineral-free inter-prism space between individual prisms in *Phospho1*$^{-/-}$ mutant mice compared to controls.

CONCLUSION: Together, these data indicate that the matrix vesicle-associated phosphatase PHOSPHO1 is essential for physiological enamel mineralization. Our findings prompt us to speculate that matrix vesicle-like organelles may not only be involved in the formation of mesenchymal mineralized tissues but also in amelogenesis.

DISCUSSION:

Mike Hubbard (Melbourne): What's the substrate of the phosphatase? Are you thinking it may be a protein phosphate?

Lauren Rosene (Dallas): The substrate of the phosphatase?

Mike Hubbard (Melbourne): Yes, what is the natural target of the phosphatase?

Lauren Rosene (Dallas): I think it's the phospholipids associated with secretory vesicle membranes such as phosphocholine and phosphoethanolamine. We think the phosphatase is snipping the phosphates off of phospholipids. We think it focuses on the phospholipids.

Mike Hubbard (Melbourne): If that's the case, what part of the process would be disrupted in the absence of the phosphatase?

Lauren Rosene (Dallas): We believe it's the initial mineralisation part, but we did find there is expression throughout amelogenesis, so it definitely affects the later maturation stage.

Mike Hubbard (Melbourne): A cellular effect or an extracellular effect?

Lauren Rosene (Dallas): We found it is an extracellular effect in the enamel.

Janet Moradian-Oldak (Southern California): Maybe it's a small detail that I missed, but you showed vesicles that may contain some mineral?

Lauren Rosene (Dallas): Yes.

Janet Moradian-Oldak (Southern California): So, in your sample preparation, do you demineralise the samples for H&E staining? Do you have to demineralise them?

Lauren Rosene (Dallas): Yes we do

Janet Moradian-Oldak (Southern California): So, how can you keep the mineral if you demineralise the samples? Maybe it's a technical thing I missed, but it's surprising that you can detect mineral in the sections when the process includes demineralization.

Lauren Rosene (Dallas): I showed the remnants of vesicle staining where you can see a little bit and definitely not all of it is still there.

Ariane Berdal (Paris): Do you know the (optimum) pH and do you have any evidence of the biochemical activity of the enzyme depending on the pH?

Lauren Rosene (Dallas): No we didn't look into that.

#49 ENAMEL DEFECTS IN *FAM83H* TRUNCATION KNOCK-IN MICE

WANG, S-K[1 2]*, HU, Y[1], SMITH, CE[3], ZENG, C[1], HU, JC-C[1] and SIMMER, JP[1]

[1]Department of Biologic and Materials Sciences, University of Michigan School of Dentistry, 1210 Eisenhower Pl., Ann Arbor, MI USA 48108; [2]School of Dentistry, National Taiwan University, No. 1, Chang-Te St., Taipei 10048, Taiwan, R.O.C; [3]Facility for Electron Microscopy Research, Department of Anatomy and Cell Biology and Faculty of Dentistry, McGill University, 3640 University Street, Montreal, Quebec H3A 0C7, Canada

Email: shihkaiw@umich.edu

Truncating *FAM83H* mutations cause autosomal dominant hypocalcified amelogenesis imperfecta (ADHCAI). No evident enamel defects in *Fam83h* null mice indicate a neomorphic mechanism for the disease.

OBJECTIVES: To investigate the pathogenesis of human ADHCAI.

METHODS: We generated and characterized a mouse model ($Fam83h^{Tr/Tr}$) expressing a truncated FAM83H protein (amino acids 1-296), which recapitulated the ADHCAI-causing human *FAM83H* p.Tyr297* mutation. Mouse molars and incisors were characterized using dissecting microscopy, backscattered SEM, histology, and molar protein extract analysis.

RESULTS: Homozygous $Fam83h^{Tr/Tr}$ mice were viable and fertile, and appeared grossly normal, except for a sparse and scruffy coat. Day 14 and 7 week $Fam83h^{Tr/Tr}$ molars exhibited rough enamel surfaces and slender cusps. On cross-sections, the lateral third of the enamel layer of $Fam83h^{Tr/Tr}$ incisor was thinner, with surface roughness and altered enamel rod orientation, suggesting disturbed enamel matrix secretion. No significant differences were observed in the electron densities of enamel in mandibular incisors among three genotypes indicating normal progression of mineralization during enamel maturation of $Fam83h^{Tr/Tr}$ incisors. However, shorter molar cusps with flat cusp tips at 7 weeks in $Fam83h^{Tr/Tr}$ mice was characteristic of post-eruption attrition. Histologically, the $Fam83h^{Tr/Tr}$ enamel organ, including ameloblasts, and enamel matrices at sequential stages of amelogenesis exhibited comparable morphology without overt abnormalities compared to those of $Fam83h^{+/+}$and $Fam83h^{+/Tr}$ mice. Crude protein extracts from first molars of Day 5, Day 11, and Day 14 mice analyzed by SDS-PAGE and amelogenin immunoblotting demonstrated a potentially reduced secretion of enamel matrix proteins during the secretory stage and delayed protein resorption during maturation stage in $Fam83h^{Tr/Tr}$ first molars.

CONCLUSION: Whereas depletion of FAM83H did not cause enamel phenotypes, enamel malformations of $Fam83h^{Tr/Tr}$ mice demonstrated that a truncated FAM83H protein, p.Tyr297*, could disturb normal amelogenesis, which underlays the pathogenesis of human ADHCAI. This study was supported by NIDCR grants DE019622 and DE015846.

DISCUSSION:

Ariane Berdal (Paris): Was there a reverse relationship between MSX2 and DLX2 expression and enamel thickness? Did you check that?

Shih-Kai Wang (Michigan): We didn't check that.

Ariane Berdal (Paris): Maybe it could be a developmental defect?

Shih-Kai Wang (Michigan): We can look into it. Thank you for your suggestion.

Agnès Bloch-Zupan (Strasbourg): Certainly there seems to be a difference between the enamel formed in the medial part of the incisor and that formed on the lateral part and few mouse models actually show this. That is something to think about. I don't know what it means, but it certainly exists.

Shih-Kai Wang (Michigan): It was actually a really interesting phenotype that we didn't expect to see. There's a difference between the medial side and the lateral side of the incisor enamel. So, we will definitely look at that.

#50 ENAMEL STRUCTURE IN A NOVEL *FAM20A* MUTATION RECAPITULATES *Fam20a$^{-/-}$* PHENOTYPE

KIRKHAM, J[1]*, MIGHELL, AJ[2], SMITH, CEL[1,3], POULTER JA[3], INGLEHEARN, CF[3], DIXON, MJ[4], BARRON, MJ[4], KLETA, R[5], HIMMERKUS, N[6], BLEICH, M[6], AL-BAHLANI, S[7] and BROOKES, SJ[1]

[1]Department of Oral Biology, School of Dentistry, St James's University Hospital, University of Leeds, Leeds, UK ; [2]School of Dentistry, University of Leeds, Leeds, UK; [3]Section of Ophthalmology and Neuroscience, St James's University Hospital, University of Leeds, Leeds, UK; [4]Faculty of Life Sciences, University of Manchester, Michael Smith Building, Oxford Road, Manchester, UK ; [5]Centre for Nephrology, University College London, London, UK; [6]Department of Physiology, University of Keil, Germany; [7]Al-Nahda Hospital, P.O. Box 937, Muscat, P.C. 111, Sultanate of Oman

Email: J.Kirkham@leeds.ac.uk

OBJECTIVES: To characterise genotype and enamel phenotype in a family with hypolastic Amelogeneisis Imperfecta (AI) and compare this with enamel phenotype in a relevant animal model.

METHODS: DNA from an Omani family with hypoplastic AI was subjected to whole exome sequencing (WES). Enamel phenotyping in human affected teeth was carried out using microCT and SEM and compared with matched controls. Incisors from *Fam20a$^{-/-}$* mice were characterised using histology, immunofluorescence, SEM and microCT.

RESULTS: WES revealed the presence of a homozygous c.907_908del; p.S303Cfs*76 variant in *FAM20A* as previously reported. SEM appearance of *FAM20A* p.S303Cfs*76 and *Fam20a$^{-/-}$* enamel both showed an initial enamel layer that was similar to, but less well ordered than, control enamel with a highly disorganised outer layer that was lost on eruption in the mice. *Fam20a$^{-/-}$* mouse incisors were chalky-white with roughened surfaces. Histologically, *Fam20a$^{-/-}$* secretory ameloblasts initially secreted extracellular matrix (ECM) proteins but rapidly detached from the underlying matrix, becoming highly disorganised; immunofluorescence showed intracellular retention of amelogenin, indicating a failure of the secretory pathway.

CONCLUSIONS: These phenotypes closely resembled those associated with a previously described *Amelx* mutation where protein export was affected, resulting in ER stress and ultimately a pro-apoptotic unfolded protein response (UPR). FAM20A is a pseudo-kinase via allosteric control of FAM20C, a true kinase that phosphorylates proteins within the ER including ECM proteins such as enamelin and others involved in protein folding/ trafficking. Failure of FAM20A to activate FAM20C could result in failure to phosphorylate 1) enamel ECM proteins, increasing their tendency to aggregate in the ER and 2) other ER proteins essential for protein trafficking, leading to ER stress. As ER stress builds after initial secretion, the UPR would switch to pro-apoptotic mode triggering ameloblast cell death. Secreted enamel ECM would fail to mineralise, resulting in the phenotype we see here.

ACKNOWLEDGEMENT: This work was supported by Wellcome Trust grant number 075945.

DISCUSSION:

Olivier Durverger (Bethesda): Do you see defects in molar eruption in this model like in humans?

Jennifer Kirkham (Leeds): We haven't looked at the molars yet. We only looked at incisor teeth. Of course there have been other papers which have looked at FAM20C, for example, and that have also looked at other animal models of FAM20A. Ariane, I don't know if you want to say anything about that.

Ariane Berdal (Paris): You showed an ectopic position of amelogenin which is like what we saw in human cases. Would you say that the enamel organ is affected? Is there evidence of another pathway that would involve cells which should not be involved in mineralisation? Do you see the same balance between ectopic and true amelogenesis processes?

Jennifer Kirkham (Leeds): Yes, there is a huge disruption (of the enamel organ). So you've got enamel proteins that are in the wrong place, you've also got cells which are dying and dead and you've got phospholipid membranes and we know that necrotic tissues often calcify. I agree with you, I think it's partly that you've got enamel protein involvement which might cause ectopic calcification and also you've got ectopic calcification, which is a result of that disruption and death that involves cell membranes.

Ariane Berdal (Paris): What would also happen like in the soft tissue, you know BSP and osteopontin depending on their phosphorylation state? I think that's the way we have to discuss this.

Jennifer Kirkham (Leeds): I believe there are other ways of FAM20C becoming activated. It may be within this system, which is a highly stressed system, that we get a more pronounced effect because it is already sensitised.

#51 DOSE-DEPENDENT RESCUE OF KO AMELOGENIN ENAMEL BY TRANSGENES *IN VIVO*

XIA, Y[1], BIDLACK, F[1 2], PUGACH, MK[1 2]*

[1]Department of Mineralized Tissue Biology, The Forsyth Institute, Cambridge, MA, USA; [2]Department of Developmental Biology, Harvard School of Dental Medicine, Boston, MA, USA

Email: mpugach@forsyth.org

Mice lacking amelogenin (KO) have hypoplastic enamel. Although overexpression of the most abundant amelogenin splice variant (M180 and LRAP) transgenes can substantially improve KO enamel, only ~40 % of the incisor thickness is recovered and the prisms are not as tightly woven as in WT enamel. This suggests that the enamel phenotype of KO mice can be improved by optimal expression levels of amelogenin transgenes, representing both the most abundant splice variants (M180 and LRAP) and cleavage product (CTRNC) at protein levels similar to that of WT.

OBJECTIVES: To understand dosage effects of amelogenin transgenes (*Tg*) representing the major splice variants and cleavage product on enamel properties.

METHODS: Amelogenin KO mice were mated with M180*Tg*, CTRNC*Tg* and LRAP*Tg* mice to generate M180*Tg* and CTRNC*Tg* double transgene and M180*Tg*, CTRNC*Tg*, LRAP*Tg* triple transgene mice with transgene hemizygosity (on one allelle) or homozygosity (on both alleles). Transgene homo- versus hemizygosity was determined by qPCR and relative transgene expression confirmed by Western blot. Enamel volume and mineral density were analyzed by microCT, thickness and structure by SEM, and mechanical properties by Vickers microhardness testing.

RESULTS: There were no differences in incisor enamel thickness between amelogenin KO mice with three or two different transgenes, but mice homozygous for a given transgene had significantly thicker enamel than mice hemizygous for the transgene (p <0.001). The presence of the LRAP*Tg* did not improve the phenotype of M180*Tg*/CTRNC*Tg*/KO enamel.

CONCLUSION: In the absence of endogenous amelogenin, the addition of amelogenin transgenes, representing the most abundant splice variants and cleavage product, can rescue abnormal enamel properties and structure, but only up to a maximum of ~80 % that of molar and ~40 % that of incisor wild-type enamel. Supported by NIH/NIDCR grant R00DE022624 (MKP).

DISCUSSION:

Derk Joester (Northwestern): Coming from the engineering side, I really appreciate the systematic approach that you are trying to do here and my question is in relation to the previous talks where accumulation of amelogenin might cause cellular stress and whether you considered that in your analysis here?

Megan Pugach (Forsyth): Yes, I mean that's where we're going with this. There is clearly some sort of perturbation in the interaction between the ameloblasts and the matrix and by adding too many transgenes, we may be interfering with the intracellular processes. So, I assume that it has to do with that, but we haven't looked into it yet. That's definitely where we are going though.

Olivier Durverger (Bethesda): Which promoter to do you use? Is it the amelogenin promoter?

Megan Pugach (Forsyth): Yes.

Olivier Durverger (Bethesda): Is it a portion of the promoter that you know is recapitulating the normal expression pattern?

Megan Pugach (Forsyth): Yes.

Mike Hubbard (Melbourne): I would like to congratulate you on your beautiful comparison of incisors and molars. I'd like to encourage more people to do that to show how practical it is.

Megan Pugach (Forsyth): It can be tricky, but we did it.

#52 MMP20 OVEREXPRESSION DISRUPTS AMELOBLAST CELL POLARITY

SHIN, M[1] and BARTLETT, JD[2]*

[1]Fukuoka Dental College, 2-15-1 Tamura, Sawara-ku, Fukuoka 814-0193, Japan; [2]The Ohio State University, College of Dentistry, 305 W. 12th Ave. Columbus, OH 43210, USA

Email: bartlett.196@osu.edu

MMP20 is a matrix metalloproteinase expressed by ameloblasts present within the enamel organ. We demonstrated before that MMP20 cleaves both enamel matrix proteins and cadherins, which are responsible for cell-cell adhesion. Previously, we engineered transgenic mice that overexpress active MMP20. Enamel from these mice was softer than normal.

OBJECTIVES: Here we examine enamel organs and ameloblasts from mouse molars to determine if tissue and/or cell-specific pathologies can be identified that may contribute to abnormal enamel formation in MMP20 overexpressing mice.

METHODS: Tissue sections were assessed for molar morphology and for keratin-14, amelogenin and MMP20 expression. Immunoblots were performed to detect both phosphorylated and non-phosphorylated COFILIN.

RESULTS: Molars from MMP20 overexpressing mice sometimes had ectopic mineral deposits within their enamel organs and the ameloblasts did not maintain their columnar morphology. Molars from wild-type mice had well defined areas of keratin 14, amelogenin and MMP20 expression whereas molars from the overexpressing mice had a more diffuse pattern of expression that could extend into the tissue layers adjacent to the ameloblasts. We found examples from overexpressing mice with striking dentin molar cusp tip abnormalities suggesting that MMP20 may influence cusp development. COFILIN maintains cell polarity, but its inhibited phosphorylated form does not. Immunoblots demonstrated that increased levels of phosphorylated COFILIN were present within enamel organs from overexpressing mice. This suggests that too much MMP20 activity inactivates COFILIN resulting in the loss of ameloblast cell polarity.

CONCLUSIONS: MMP20 overexpression likely disrupts cell-cell contacts, which allows depolarized ameloblasts to migrate into adjacent tissues of the enamel organ resulting in abnormal mineral deposits and ameloblast gene expression in these areas. The mechanism of how increased MMP20 activity leads to COFILIN inactivation remains to be determined. However, we conclude that the pathologically depolarized ameloblasts likely contribute to the abnormally soft enamel present in MMP20 overexpressing mice.

ACKNOWLEDGEMENT: This research was supported by National Institute of Dental and Craniofacial Research of the National Institutes of Health under award number R01DE016276 (JDB).

<u>DISCUSSION:</u>

Bernhard Ganss (Toronto): You showed that the ameloblasts overexpressing MMP20 initially form more or less properly and when they transition into the maturation stage they deposit a basement

membrane. Could it be that overexpression of MMP20 degrades this basement membrane and that this leads to the detachment of the ameloblasts?

John Bartlett (Ohio): That’s a possibility I hadn’t entertained, could happen, but they are also detaching from each other as they migrate into the stratum intermedium/stellate reticulum. But, that’s a possibility that I hadn’t really thought of, so that’s something I’ve got to put some thought into. Thank you.

Raquel Gerlach (Sao Paulo): I just wanted to tell you that we recently found that MMPs degrade cadherins in the heart. So, it was interesting to find that. It is very nice that you are pursuing this because it looks to be very important.

John Bartlett (Ohio): We knew that other MMPs can cleave cadherins which is why we tested whether MMP20 could also do that.

Colin Robinson (Leeds): Will this not also have destroyed any contact with the matrix as it is quickly secreted so that any attachment or any association with the matrix would be lost?

John Bartlett (Ohio): Yes, here I showed you MMP20 overexpression in molars, but we’ve recently published results from incisors that overexpress MMP20. What happens with the incisors is that ameloblasts and other nearby cells, such as fibroblasts that surround capillaries present in the papillary layer, all migrate into the enamel space. This disrupts the whole enamel maturation process and the enamel becomes very soft, with a thin layer of enamel over the dentine and a thin layer of enamel atop where the cells migrated. So, in the incisor where eruption happens faster, the migration happens faster and takes over.

#53 AMELOBLASTIN OVEREXPRESSION IS LINKED TO ENAMEL HYPOMINERALIZATION

CHUN, YP*

Department of Periodontics, School of Dentistry, University of Texas Health Science Center at San Antonio, USA

Email: chuny@uthscsa.edu

Enamel hypomineralization is highly prevalent in children of many populations worldwide. Enamel defects with reduced mineral content are sharply demarcated against sound enamel of permanent molars and incisors and were termed molar-incisor hypomineralization (MIH) by the European Academy of Pediatric Dentistry (2001). The etiology of MIH is not known. Ameloblastin (Ambn) is one of the enamel proteins that are important for enamel formation.

OBJECTIVE: The goal was to characterize enamel defects caused by overexpressed ameloblastin in a mouse model.

METHODS: Transgenic *Ambn* was expressed in C57BL/6 mice from the 4.6 kb amelogenin promoter. *Ambn* was overexpressed in 4 different mouse lines in increasing concentrations. The enamel of mandibular molars and incisors was analyzed at secretory and maturation stages by microCT, scanning electron microscopy, Western Blot analysis and histology. Cells of the enamel organ were analyzed by histology and RNA-seq.

RESULTS: With an increase in AMBN concentration demarcated defects were found in molars and incisors. The defects became larger in size as the AMBN concentration increased. At the highest concentration the enamel surface was porous and delaminated from dentin. The defect contained less mineral throughout the thickness of the enamel layer compared to sound enamel. During maturation stages, enamel matrix was retained and cysts formed within the enamel organ. Genes of enamel proteins and endocytosis pathways were dysregulated.

CONCLUSIONS: The expression of *Ambn* is highly regulated to provide the correct concentration in enamel formation. Enamel defects caused by the overexpression of *Ambn* is consistent with MIH-like defects that are sharply demarcated, reduced in mineral content and contain retained enamel matrix. This study was supported by NIDCR grant K08 DE022800 (YPC).

DISCUSSION:

Sylvie Babajko (Paris): It's very interesting to see that you have a completely different model from us and that you get quite similar modulation of enamel matrix protein and protease expression, especially in the maturation stage. So, what is your comment? Do you feel that there is some similar way of regulation? I observed that there is a down regulation of amelotin, as well as a down regulation of KLK4, so what is the relation, what is your comment about that?

Yong-Hee Chun (San Antonio): I think that the dysregulation in our model has already started at the secretory stage because with the amelogenin promoter, we are targeting the secretory stage, and then at the maturation stage the problem becomes augmented. So, it could be a secondary effect, but I

think it starts at the secretory stage, and I think it's not clear. The direct and indirect effects are not clear at this point.

Sylvie Babajko (Paris): Yes, and you have an inverse relation when you compare maturation and secretion, so there are probably many things to discuss.

#54 ROLE OF TIGHT JUNCTION CLAUDINS DURING AMELOGENESIS

BARDET, C[1]*, WU, Y[1 2], DIALLO, MT[1], RIBES, S[1], FIGUERES, ML[3], BREIDERHOFF, T[4], MULLER, D[4], HOUILLIER, P[3 5] and CHAUSSAIN, C[1 6]

[1]EA 2496, Laboratory Orofacial Pathologies, Imaging and Biotherapies, School of Dentistry, Université Paris Descartes, Sorbonne Paris Cité, France; [2]Department of Oral and Cranio-maxillofacial Science, Ninth People's Hospital, Shanghai Jiao Tong University School of Medicine, Shanghai Key Laboratory of Stomatology, Shanghai, China; [3]Sorbonne Universités, UPMC Université Paris 06, INSERM, Université Paris Descartes, Sorbonne Paris Cité, UMR S1138, Centre de Recherche des Cordeliers, CNRS ERL 8228, Paris, France ; [4]Department of Pediatric Nephrology, Charité University School of Medicine, Berlin, Germany; [5]AP-HP, Department of Physiology, and reference center of children and adult renal hereditary diseases (MARHEA), European hospital Georges Pompidou, Paris, France; [6]AP-HP, Odontology Department, and Reference Center for rare diseases of the metabolism of calcium and phosphorus, Nord Val de Seine Hospital (Bretonneau), France

Email: claire.bardet@parisdescartes.fr

BACKGROUND: Loss-of-function mutations in the claudin 16 (*CLDN16*) gene cause Amelogenesis Imperfecta (AI) (Bardet *et al.*, 2016) associated to Familial Hypomagnesemia with Hypercalciuria and Nephrocalcinosis (FHHNC), a rare disease characterized by renal Ca^{2+} and Mg^{2+} wasting, nephrocalcinosis and early renal failure. Similarly, Yamaguti *et al.* showed that claudin 19 deficiency resulted in AI in patients with FHHNC due to *CLDN19* mutations.

OBJECTIVES: We aim to understand the function of claudins at tight junctions (TJ) of secretory ameloblast.

METHODS: The enamel phenotype of *Cldn16*$^{-/-}$ mice, the murine model of FHHNC, was analyzed. We measured enamel matrix pH using BCECF and analyzed its protein content. Ion transporter expression was studied in secretory ameloblasts by transcriptomic analyses and immunohistochemistry. The thick ascending limb of Henle's loop (TALH) was used as a model to measure paracellular permeability to H^+ in *Cldn16*$^{-/-}$ mice.

RESULTS: We reported the expression of Cldn16 in the murine tooth germ located at the distal end TJ of secretory ameloblasts and showed that the absence of Cldn16 strongly impaired TJ organization. The *Cldn16*$^{-/-}$ forming enamel matrix displayed a significantly lower pH value associated with decreased MMP20 activity, and accumulation of enamel matrix proteins. The paracellular permeability to H^+ was similar in the TALH of *Cldn16*$^{-/-}$ and *Cldn16*$^{+/+}$ mice. We are currently studying, in *Cldn16*$^{-/-}$ mice, the potential alteration of ion transporter expression involved in the pH cycling necessary for normal enamel formation. In parallel, we observed that *Cldn3*$^{-/-}$ mice displayed a dental phenotype similar to that of *Cldn16*$^{-/-}$ mice

CONCLUSION: Our preliminary results indicate that the lack of CLDN16 at the TJ results in lower pH value in the apical compartment, likely because transcellular net acid transport is impaired. Our data highlight the importance of TJ proteins in tooth formation.

Bardet *et al.* J Bone Miner Res, 2016 Mar; 31(3):498-513 ; Yamaguti *et al.* (submitted)

DISCUSSION

John Bartlett (Ohio): There are some theories that tight junctions are released to allow protein secretion and that tight junctions in the maturation stage are released to allow protons to come out of the enamel matrix. Have you done any studies on proteins, or looked at how the ameloblasts modulate through the maturation stage?

Claire Bardet (Paris): We focused on secretory stage ameloblasts because claudin 6 is not expressed in the maturation stage. So, we found a rescue of the mineralisation growth phase.

John Bartlett (Ohio): I wonder if you might see differences from the normal protein content in the enamel. Did you check that?

Claire Bardet (Paris): Yes, we checked that in the secretory stage, but not in the maturation stage, and all enamel matrix proteins were increased.

Rozita Jalali (Amsterdam): I was wondering if you had any H&E or histological sections from your knock out mice. What do they look like? What does the ameloblast layer look like?

Claire Bardet (Paris): We didn't check that.

PANEL DISCUSSION:

Elia Beniash (Pittsburgh): I have a question to everybody, to all the panellists, to everybody in the audience. It seems that there is a theme regarding these matrix protein knockouts like ameloblastin, or over expression modifications, and when enamelin is knocked down the cells lose polarity. So, we don't know if the reason enamel does not form (in these cases) is due to this (ER) stress or due to the effects of the matrix. How can we distinguish this because a lot of the cells are stressed and basically putting additional stress on them by over-expressing or under-expressing and by changing the type of interactions between these proteins leads probably to making the cells sick? You can see it just by looking at them under the microscope. But the original point of knocking down these proteins was to try to understand the function of the matrix. Can we really do this work? Do we need to look into the cell first and then see what's going on in the matrix?

Jennifer Kirkham (Leeds): Thank you Elia. I agree with a great deal of what you said. I do think we need to raise our consciousness of what's happening in the cellular compartment because it is going to potentially have very big effects on the extracellular matrix and consequently biomineralisation. It's already been published previously that ameloblasts are stressed during normal amelogenesis. They are highly stressed cells and indeed they share that in common with other secretory cells that do a lot of work. If you look at any other highly secretory cells, they are also stressed and they are undergoing the unfolded protein response (UPR). So, they are going to be very susceptible if we start to make them do even more work. The UPR is already initiated during normal amelogenesis, so we can push them very easily into becoming cells that are apoptotic. We need to check whether the UPR is initiated and it's easy to do. We can check on apoptosis, check on things like ER volume for those who are looking at histology. We also need to look at PCR results for all of the different (UPR) markers. These are easy things to do but we need to check them if we are then going to be able to interpret our models. We did this with COS7 cells after transfecting them with our mutant amelogenin, which we know in ameloblasts causes them to go into stress and die. But the COS7 cells didn't die until we started to transfect them with extra over-expressing proteins. As soon as we included ameloblastin, the protein load tipped them into apoptosis. My plea would be to say, let's take this into account. It doesn't invalidate what we're doing, it's an additional consideration.

Tom Diekwisch (Dallas): Two comments, one comment to Janet's earlier question, we do regular demineralisation of paraffin sections and all mineralised tissues are preserved, including dentine and enamel and also the vesicles in the stratum intermedium/stellate reticulum interface and actually we don't find them by using exactly the same procedure in the wild type mice. My question is, five years ago, in the previous enamel meeting, we presented an over-expresser, an ameloblastin over expresser and we crossed this over-expresser with an amelogenin knockout. So, we had matrix that was just ameloblastin and that matrix was characterised by short and highly mineralised crystals close to those that occur early in evolution and I was just wondering what you were thinking in your model about whether you had observed a similar scenario of thickening of crystals and shortening of crystals.

Yong-Hee Chun (Texas): Thank you for the question. So, I think there are a few differences between the models in terms of the promoter. I think in your mouse model you've used the *Krt14* promoter, is that correct?

Tom Diekwisch (Dallas): Yes

Yong-Hee Chun (Texas): In our model it's the amelogenin promoter. At this point we have not analysed crystal formation in our model so I am not able to really compare directly and comment on it.

Janet Moradian-Oldak (Southern California): This is a small detail, but very important that I would like to reinforce, especially because we started with an introduction with three major proteins and the conclusion based on knockout animals. Specifically, I refer to the presentation by Hu, about nanohardness, beautiful images, beautiful work and I appreciate how much effort it takes to work with animals and obtain information from them. However, we really have to be extremely careful when we analyse mineral phases in those animals. For those of you who are not familiar, it is extremely hard to distinguish between apatite and OCP. The only way you can do it is with small angle X-ray scattering and there's a specific diffraction pattern. I won't get too technical, but the X-ray you showed is really not conclusive that the mineral is OCP. So, it's just a comment to be careful when you make those statements. It's obvious that the mineral is very different. Maybe it's just an accumulation of pathological mineral formation, which could be a mixture of many calcium phosphate phases and it's a strong indication that when we don't have amelogenin, things just go wrong. It could affect influx of ions, if the cells are not stressed, it can affect local super saturation and the physical processes of crystal formation. So, the strong statements that we make, such as this protein does not do this and this protein does do this, we have to be a little bit more cautious about those interpretations and take things into account.

Jim Simmer (Michigan): I don't think that there was any problem with the lack of caution at all. We've clearly shown what we did and what we discovered and we understand, as well as anybody, that pathology is part of what you see. We clearly discussed in our published paper the items that you are talking about, where things that are secondary could be going on. Calcium and phosphate concentrations could be going up, pH could be going down and there are many factors that lead to aberrant mineralisation. But, what we're more impressed with, for instance, is that the ribbons form in the amelogenin null mouse and there are certain things that you can say, especially when you are looking at very early formation before there's a lot of pathology developed, that offer constraints on how you want to interpret the roles of these proteins and I think those are perfectly valid. We have been very careful about the way we express what's going on and have not been trying to say these are necessarily direct effects. But, it's clear that the mineral phase is very different in the amelogenin null mouse and we are not saying amelogenin is controlling that, but that is a possibility. It explains why the mineral is different. The mineral is not apatitic and this needs to be explained with further work, but I don't think there are strong conclusions that aren't warranted here.

Yuanyuan Hu (Michigan): I would like to comment on Dr Oldak's question about x-ray diffraction. I don't understand very much about this technique, so we are working with an expert at the Argonne National Lab in Chicago who has worked with x-ray diffraction for over thirty years. In terms of sample preparation, we are fully aware about how sensitive the mineral phase can be. So, what we did was freeze dry the samples and then sectioned through the layer. The person at the national lab is

particular. He said you cannot use any glass and you cannot embed the samples. To interpret the results, we used a double blind procedure because I don't know much about x-ray diffraction and he does not know much about enamel. So, we asked him to analyse the data. He analysed the mineral phase and concluded that amelogenin null enamel is composed of octacalcium phosphate.

Ariane Berdal (Paris): So, going back to the cells, and I would like Professor Harada to react, don't you think that in your MMP20 overexpression mice you may also get changes in the differentiation pathway of the enamel organ cells? When you look at odontogenic tumours, some have mutated ameloblastin and Professor Harada, some years ago, could switch HAT7 differentiation by manipulations, which means that the enamel matrix could be signalling the cells which are behind the ameloblasts to stimulate the differentiation process and ectopic differentiation processes. What would you say?

John Bartlett (Ohio): Yes, that's part of it. We do see pathology in the secretory stage that progressively gets worse the closer you get to the maturation stage. It seems like MMP20 is not really turned off, but you don't really know where you are in the developmental stage, at least with the incisors, because everything becomes so pathological. In molars it's more subtle because the enamel is forming, but it could have to do with the developmental stage and what Bernhard said might have a big effect too. The basement membrane is supposed to form, and if it can't be laid down properly, that could cause the pathology. Does that answer your question?

Ariane Berdal (Paris): Yes, revisiting, for instance, Wnt pathway, BMPs and distribution and so on, because for instance, amelogenin is linked to the Wnt pathways so it could be like abnormal activation of the differentiation process.

John Bartlett (Ohio): Yes, exactly and that's what we are trying to prove. Right now we know for a fact that MMP20 can cleave cadherins. We can cleave them *in vitro* and precipitate the cleaved cadherins. We are almost certain cadherins are cleaved *in vivo*, because there's so much circumstantial evidence. The only thing we haven't absolutely proven is that B-catenin goes to the nucleus and activates its canonical transcription factor site, which is the LEF/TCF binding site, and we're working on that.

Alex Vieira (Pittsburgh): Very, very nice sections. As someone who is mostly dealing with humans, it's very exciting to see the teasing out of the pathogenesis of those molecules and so I have a couple of questions. One is the language. I think Dr Wang mentioned that *FAM83H* is a candidate gene responsible for a form of amelogenesis imperfecta (AI). When you say "a candidate gene", it makes me think that you still do not believe that the gene is responsible for the pathology and I wanted to confirm this. I wonder if you are working on trying to conclusively prove that this gene is really responsible for the pathogenesis, or if you are happy with the human data and the mutation analysis linkage that was done. Do you just want to figure out the pathogenesis of it? The data kind of threw me off as something that I still don't know 100 % for sure if it is this gene. And, one of you showed a very nice measurement of the amelogenin knockout that produces 66 % of the protein instead of 100

%. Do you believe that you could have levels of expression with no clinical effect at 75 %, 85 % or 90 % and would that create a gradient for susceptibility for enamel pathology?

Shih-Kai Wang (Michigan): So the term "candidate gene" means that when you do mutation analysis, if you see patients with the hypocalcified AI phenotype, the first gene that you would test is *FAM83H* and that is what I think "candidate" means. Also, there is very strong evidence that *FAM83H* mutations causes this specific type of AI. There are more than 20, I think right now it is 23, different disease-causing mutations in this specific gene and there's very high mutation homogeneity, so the human data is very solid.

Alex Vieira (Pittsburgh): So, the candidate genes are to identify the cause of AI, but the disease is known to be caused by candidate gene mutations?

Shih-Kai Wang (Michigan): Yes.

Megan Pugach (Forsyth): My presentation did not show the 66 % protein expression, but we have also found that the hardness of the (enamel in) amelogenin knockout mice is not that bad actually. So, I think absolutely there could be some sort of gradient in a patient who has a mutation in amelogenin that may not be causing AI, but could cause caries susceptibility for example.

Stephen Hsu (Singapore): I have a technical question for Claire Bardet. I'm very curious how the pH measurement was done and do you think it could be done in dentine tissue?

Claire Bardet (Paris): We used BCECF (2'-7'-bis(carboxyethyl)-5(6)-carboxyfluorescein) and we directly put the incisor in the BCECF solution and then we measured the pH using a specific microscope.

Stephen Hsu (Singapore): Is it a fluorescent dye?

Claire Bardet (Paris): Yes, it is a fluorescent dye.

Elia Beniash (Pittsburgh): My comment is to Megan Pugach and everybody, regarding hardness differences in amelogenin knockouts or other mutants. It's not surprising the stiffness and hardness are primarily determined by the mineral content. You should do fracture testing for this type of measurement if you are expecting to see differences. So, it's not surprising, that's all.

Henry Margolis (Foryth): I just want to make a general comment. I think today we've heard a lot about the limitations of analysing knock-in / knockout models, because we don't know what's going on in the cell and we don't know everything that is going on in the matrix, but I would like to tell you

how I look at it. What happens to mineralisation is a reflection of all that, and we have extracellular mineralisation. What's going on there is that we have phase changes and we should be able to explain that in chemical terms or protein terms, what they might be doing. So, it's a dynamic situation so when we look at very early mineralisation in a knock-in or knockout and/or wild type we say they are similar. I would like to propose that perhaps it is similar, there may be factors in amelogenin expression that at early stages might be quite low at that point and whenever we see certain things that might be similar so I think it's also good to put together a time course to see if things are changing. If things are similar initially, but very different just a few nanometres down the road, I think that we have to conclude that protein that's missing there, and when we know what proteins are there and what's missing, we should then identify that. It's just knocking out something and saying this is a knockout that's not there, if we have the information it should be right there saying what we know or what we don't know what it is, because it is extracellular matrix mineralisation, it's all regulated by the cells. Whatever's going on there is a reflection of all what you have presented there so I just want to point that out and I'm happy to hear your comments.

Yuanyuan Hu (Michigan): One thing we checked with the focused ion beam is the onset of enamel formation. I don't think there is much difference depending on whether amelogenin is expressed at this early stage. So, it is important to know the stage of enamel development for protein function.

Mike Hubbard (Melbourne): I think today is a red letter day for the molar hypomineralization field. From every talk I could get something that was relevant to the field. Endorsing what I said yesterday, I think people can think of their work for the topic and perhaps what Alex said, can think in terms of your own topic, but also about how it might apply outside the field. So, comparative analysis of normal enamel and the various defects I think will give us all a lot of progress. And, I did make comment about the comparison between molars and incisors and you weren't the only one, but that's great, so I think in the history of our field there's quite a few examples of rodent incisors that have given results that are a little bit different and we can learn from them being different. But, just because incisors may be easier, I think we should make that comparison. And then, Yong Hee has given me so much pleasure. We've put the world map up from 2013 with the 59 (reported) studies (on molar hypomineralization) and every talk I've given since, and there's been a few comments "how come America's not on the map"? We've known from the literature that molar hypominerization is manifest in America. The fact that a paper hadn't been published about it has caused a lot of questioning and it has been useful to say that because it's sort of brought the education issue to the fore. So, the fact that there's going to be a publication out of the States is very pleasing and I think the other comment I'd make is, we look at molar hypomineralization as a mineralisation problem, so we think maturation stage, but one of the people who really got this field going scientifically is Grace Suckling. In the 80s she predicted that hypomineralization could actually come from a defect in the secretory stage. Yong Hee's studies give us reason to think more about that as well and I think, having discussed with her this morning, we could all think about how various procedures we could use to now narrow down, and do what I see as micro-proteomics. Can we get to those demarcated opacities in a mouse incisor? So, we are getting ultra-micro, how can we go down there and ask what's different about that opacity versus the seemingly normal enamel beside it. A real technical challenge that multiple people could address.

Kaushik Mukerhjee (Southern California): I have a question for Dr Pugach. For the LRAP studies we have seen that LRAP alone cannot rescue enamel in animal models and yet it plays a very important role, along with amelogenin, in restoring microstructure, thickness and mechanical properties. So how would you explain the functional role of LRAP *in vivo* and what role do you think it plays?

Megan Pugach (Forsyth): We've found the same thing as you. LRAP on its own doesn't rescue and doesn't improve structure. Full length amelogenin on its own can give pretty close to normal thickness, but LRAP we think is more involved in structure and improving the decussation pattern. Mechanistically we don't know how it works. We haven't really looked into that, but it clearly is doing something. M180, as Malcolm Snead showed, is almost sufficient to rescue most of the hardness, but you still lack some structure.

Session 8: Enamel Mineral

Moderator: Elia Beniash, University of Pittsburgh, USA

#55 BIOMINERALISATION IN DOMESTIC PIG MOLARS

SOVA, S*, KALLONEN, A, JERNVALL, J and HEIKKILÄ, P

#56 MAMMALIAN ENAMEL MATURATION: CRYSTALLOGRAPHIC CHANGES PRIOR TO TOOTH ERUPTION

KALLISTOVA, A*, HORACEK, I, ŠLOUF, M, SKALA, R and FRIDRICHOVA, M

#57 ANALYSIS AND ROLE OF AMORPHOUS INTERPHASES IN DENTAL ENAMEL

GORDON, LM, COHEN, M, FREE, R, DEROCHER, K, and JOESTER, D*

#58 WITHDRAWN

#59 PROPERTIES OF THE AMORPHOUS INTERGRANULAR PHASE IN HUMAN DENTAL ENAMEL

DEROCHER, KA* and JOESTER, D

#60 ENAMEL DISSOLUTION: NOVEL KINETIC METHOD TO MEASURE APPARENT ENAMEL SOLUBILITY

HASSANALI, L, LYNCH, R, ANDERSON, P*

#61 HOW TO OBTAIN THE BEST POSSIBLE INFORMATION FROM DENTAL ENAMEL

GERLACH RF

#62 SUPERFICIAL ENAMEL TRACE ELEMENTS CHANGED OVER TEN THOUSAND YEARS (Pb, Mn, Zn, Cu, Cu, Sr, Cd, Ba, and Ag)

RAMOS, J*, BILAL AS, TOLEDO, TCI, VEIGA MAMS, MARANHAO, TA, RAMOS, JC, LINE, SRP AND GERLACH, RF

#76 INVESTIGATING WDR72'S FUNCTION IN PROTEIN REMOVAL DURING ENAMEL MATURATION STAGE

KATSURA, KA*, ZHU, J, NAKANO, Y, ZHANG, Y, AND DEN BESTEN, P

#55 BIOMINERALISATION IN DOMESTIC PIG MOLARS

SOVA, S[1 2]*, KALLONEN, A[3], JERNVALL, J[1] and HEIKKILÄ, P[2]

[1]Institute of Biotechnology, University of Helsinki, Helsinki, Finland [2]Department of Geoscience and Geography, University of Helsinki, Helsinki, Finland [3]Department of Physics, University of Helsinki, Helsinki, Finland

Email: Susanna.Sova@helsinki.fi

OBJECTIVES: The domestic pig (*Sus scrofa domesticus*) is a plant-dominated omnivore with large, bunodont molars. The relatively fast tooth development, together with their large size, makes pig teeth a suitable model for developmental studies and for comparisons of different methods. The aim of this work is to compare three-dimensional computational x-ray microtomography (microCT) reconstructions, Vickers hardness measurements and photomicrographs of polarized light microscopy of pig molars at different stages of maturation.

METHODS: Domestic pig molars were scanned with mineral standards using custom-built microCT system and 3D-electron density models were constructed using ImageJ software. Electron density values of the microCT models were compared with photomicrographs of polished thin sections of the teeth and Vickers hardness measurements of epoxy mounts. Polished thin sections were examined with a polarised light microscope.

RESULTS: In the maturing enamel, the electron density values of the microCT reconstructions reflected the hardness measurements. The electron density values and hardness of dentin were comparable among all the teeth. Compared to enamel, dentin was harder than the electron density values would indicate. Enamel matrix secretion was completed before its electron density values reached the values in dentin. The enamel electron density values continued to increase in erupted teeth. The optical properties of thin sections largely followed microCT and hardness data. However, the neonatal line had more coherent crystal orientation than the surrounding enamel.

CONCLUSIONS: Despite the relatively large size and thick enamel of pig molars, the enamel maturation begins only after the matrix secretion is completed. The electron density values and hardness measurements from enamel and dentin appeared comparable within each tissue, but not across the tissues. MicroCT imaging allows detailed characterization of the development of the large and rapidly forming pig molars.

ACKNOWLEDGEMENTS: Supported by the Academy of Finland and University of Helsinki

DISCUSSION:

Colin Robinson (Leeds): Just to talk about pigs for a moment. We did some work on (the teeth of) Danish Landrace pigs some years ago and we found out that they never seem to mature. The enamel mineral levels got to about 50 or 60 percent but then it erupted. I wondered whether you found a similar thing here, or is this peculiar to Danish pigs and would you like to comment on that. The teeth worked perfectly well by the way, because one of them chased me and bit me. It wasn't funny at the time. But if you could comment on that I would be grateful.

Susanna Sova (Helsinki): I think I read one of your papers where you say that the percentage of the mineral goes further (increases) after tooth eruption, but I don't know how much after. These teeth were from 6 month old pigs so the first molar was just erupted, but I have some other pigs that are 11 months old and that's one of the things that I will compare next when I go home.

In humans, if I understood right, it takes 6 months to continue mineralisation (of enamel) in the mouth after the eruption.

Colin Robinson (Leeds): Are these Finnish pigs?

Susanna Sova (Helsinki): Yes, they are from a Finnish sausage factory!

Jan Hu (Michigan): I found it very interesting that in a previous slide you indicated the micro CT. Compared to enamel, dentine is higher in electron density value as reflected by the method. I wonder whether you can elaborate as to why that happened?

Susanna Sova (Helsinki): That's an interesting question, and first I thought I did some mistake but then I realised actually that in dentine the mineral matter it has a bigger role in the structure and actually the collagen fibres, they form before the mineralisation starts and they are a big part of the structure, so in that way it made sense because mineral matter has always higher electron density than organic matter.

Steve Brookes (Leeds): Can I just ask a technical question? How did you calibrate the CT scans?

Susanna Sova (Helsinki): I calibrated them with three different minerals to make sure that always the highest point and the lowest point is the same. I had siderite ($FeCO_3$) for the highest point, that has the highest electron density and then I had fluorapatite ($Ca_5(PO_4)_3F$), which is more common in stones than hydroxyapatite. I had that to be on the same (density) level as the enamel and then I had quartz (SiO_2) for the lowest point, because quartz electron density is actually quite near to the dentine density.

Steve Brookes (Leeds): And the geometry and size?

Susanna Sova (Helsinki): They are always inside the scan, so inside all the scans I had the same three standards and then I calibrated the sample scans compared to those minerals.

#56 MAMMALIAN ENAMEL MATURATION: CRYSTALLOGRAPHIC CHANGES PRIOR TO TOOTH ERUPTION

KALLISTOVA, A[1 2]*, HORACEK, I[3], ŠLOUF, M[4], SKALA, R[1 2] and FRIDRICHOVA, M[2]

[1]Institute of Geochemistry, Mineralogy and Mineral Resources, Faculty of Science, Charles University in Prague, Albertov 6, Prague 2, CZE; [2]Institute of Geology of the CAS, v.v.i., Rozvojová 269, Prague 6, CZE; [3]Department of Zoology, Faculty of Science, Charles University in Prague, Viničná 7, CZE; [4]Institute of Macromolecular Chemistry of CAS, v.v.i., Heyrovského náměstí 2, Prague 6, CZE

Email: kallistova.anna@gmail.com

OBJECTIVES: We analyzed crystallographic characteristics of enamel apatite at different stages of pre-eruptional development and at different tooth location (mesial (Mp) / distal (Dp) parts; inner (Ip) / outer (Op) enamel of talonid) using the lower distal molar of the miniature pig as a model.

METHODS: Enamel samples underwent following analyzes: 1) X-ray powder diffraction to get information about microstructural characteristics of apatite; 2) microindentation hardness testing to obtain indentation hardness H_{IT}, indentation modulus E_{IT}, indentation creep C_{IT}, and the elastic part of indentation work η_{IT}; and 3) scanning electron microscopy to trace changes in spatial aspects of enamel architecture.

RESULTS: We observed (i) a gradual growth of crystallite thickness (Fig. a) with a mean monthly increment of about 3.15/3.44 nm at Ip/Op and 4.4/3.8 nm at Mp/Dp, continuous throughout the pre-eruptional period, combined with (ii) an abrupt decrease in the extent of lattice imperfections (Fig. b) and (iii) a rapid switch in enamel mechanical properties (significant decrease in H_{IT}, E_{IT}, η_{IT} and increase in C_{IT}; Fig. c,d). (iv) The Ip-Mp part was about 4 months ahead in embryonic development compared to Op-Dp. (v) The SEM images of embryonic enamel showed the fully developed rods, even for the youngest individuals, going from EDJ toward the surface area. The deepest enamel zones (at 16 -17 months) exhibited the adult-like appearance with mineralized infill (Fig. e) while the Op had incomplete development (Fig. f) of interrod matrix (IRM).

CONCLUSION: Our results suggest that enamel formation of less derived enamel types (contrary to human or rodent enamel) is characterized by two distinct processes: (a) the early establishment of an enamel scaffold formed by loose radial rods with a gradual growth of crystallite thickness, and (b) the mass crystallization of the IRM at the perieruptional stage, by which the mature enamel attains its final hardening.

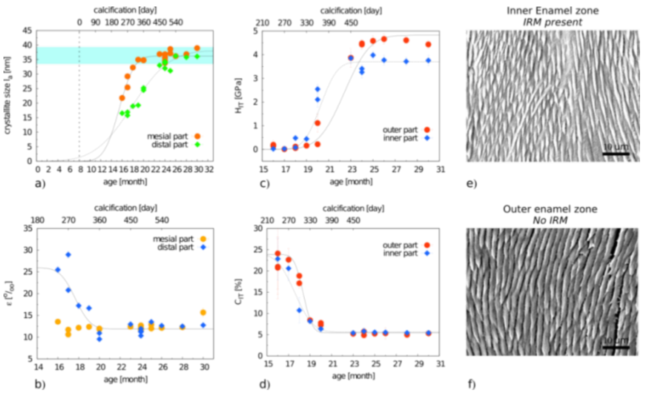

DISCUSSION:

Steve Brookes (Leeds): The delay occurring between the initial appearance of the rod enamel and the inter-rod enamel; is that because of the morphology of the Tomes' process?

Anna Kalistova (Prague): According to our methods, we are not able to say that because I didn't do the histological sections or anything like that.

Steve Brookes (Leeds): Do you have any idea what the delay is? In either distance or time?

Anna Kalistova (Prague): Yes of course. We had different ages of pigs. Let's say there was a band which was between the compact enamel and the one without the interrod enamel and the transitional zone where the interrod enamel starts to form, so we were able to see how it moved through the surface with the age of the animal.

Steve Brookes (Leeds): The rod enamel is formed at the end of the Tomes' process while the interrod enamel is from the walls of the Tomes' process. Is this related to your findings?

Anna Kalistova (Prague): I don't know, we didn't look at the ameloblasts so I really don't know.

Elia Beniash (Pittsburgh): Pigs and humans are omnivores so our physiologies are very similar or so I've heard. Can you speculate on the difference between humans and pigs, in terms of how the teeth mineralise? One can assume, since the function is similar, the structure and mineralisation processes are the same?

Anna Kalistova (Prague): I can say that if I compare the crystals or the crystal by itself, it is the same, so the mechanism of the crystal formation is probably similar. The only difference is with the delaying of the interrod enamel.

Elia Beniash (Pittsburgh): But does it have any functional relevance, or it is just random evolutionary differences that don't count?

Anna Kalistova (Prague): I don't know.

#57 ANALYSIS AND ROLE OF AMORPHOUS INTERPHASES IN DENTAL ENAMEL

GORDON, LM, COHEN, M, FREE, R, DEROCHER, K, and JOESTER, D*

Materials Science and Engineering, Northwestern University, Evanston, IL 60208, USA

OBJECTIVES: Improve the resolution of current models of the phase composition and chemical structure of dental enamel to the nanoscale.

METHODS: Atom Probe Tomography (APT), is a destructive mass-spectrometric imaging technique in which atoms are evaporated and ionized, one by one, from a sharp needle-shaped specimen, and their mass-to-charge-state ratio is determined by a time-of-flight experiment. At the same time, the original location of the atom is determined with sub-nanometer resolution. APT was used in conjunction with a range of spectroscopic techniques, nano-indentation, and analysis of enamel dissolution rates.

RESULTS: At simple grain boundaries (GB) in regular enamel, Mg^{2+} is enriched ~20 times over the solubility limit. In pigmented enamel, Mg^{2+} is replaced by iron at even higher concentration.[1-2] At multiple GB, Mg is enriched ~60 fold and Fe is enriched at least 80 fold. Spectroscopic analyses indicate that the local structure around Mg and Fe is disordered. Carbonate ion, residual organics, and water are all elevated at multiple, but not at simple GB. In enamel treated topically with fluoride, APT indicates that F^- and Mg^{2+} co-localize. Pigmented enamel is significantly harder and more resistant to dissolution than regular enamel.

CONCLUSION: APT is a powerful tool to investigate compositional gradients at the smallest length scales in enamel. In combination with correlative spectroscopy techniques, APT provides strong evidence for the presence Mg-substituted amorphous calcium phosphate (Mg-ACP) as an intergranular 'glue' between hydroxyapatite crystallites in regular enamel, and of Ca-substituted amorphous ferric phosphate (Ca-AFP) in pigmented enamel. The presence of Mg and Fe has a dramatic impact on mechanical properties, resistance to acid dissolution, and diffusion of fluoride.

[1]L. M. Gordon, D. Joester, *Frontiers in Physiology* 2015, *6*.; [2]L. M. Gordon, M. J. Cohen, K. W. MacRenaris, J. D. Pasteris, T. Seda, D. Joester, *Science* 2015, *347*, 746-750.

DISCUSSION:

Wendy Shaw (Pacific Northwest National Laboratory): This is really beautiful work. My question is, do you think that you would have the same resolution for full proteins, not just carbon, nitrogen, oxygen, but really looking at the proteins?

Derk Joester (Northwestern): Yes, the field evaporation process will break most things down to smaller fragments, so for example, in the chitin work, the organic fibrils (which are a mix of chitin and proteins), we only saw relatively small fragments. If we have larger fragments, they might form but we might lose them in the noise and if we only really have one hit, over a certain size it would be very difficult. People have also looked at ferritin and things like that, so for now we see mostly small fragments. That's a limitation of the technique that helps us get higher resolution essentially.

Henry Margolis (Forsyth): I thought I saw on your slide; did you say the solution contains 250 ppm fluoride?

Derk Joester (Northwestern): I didn't have time to talk about all of this. This was a rabbit incisor with a window that dissolved under 250 mM lactic acid, pH 4. We compared that in our first experiment with beaver enamel that's pigmented under the same conditions. What we did, we took the beaver enamel and we just polished off the pigmentations so we ended up here (pointing to the slide). [It is] just showing that it's only the pigmented layer that has a low dissolution rate. Then we took this and treated it very crudely with sodium fluoride, just to see what would happen, and when we do that, we end up here (pointing to the slide). So we depressed the solubility again. That was the process that I didn't have time to talk about.

#58 WITHDRAWN

#59 PROPERTIES OF THE AMORPHOUS INTERGRANULAR PHASE IN HUMAN DENTAL ENAMEL

DEROCHER, KA* and JOESTER, D

Department of Materials Science and Engineering, Northwestern University, Evanston, IL 60208, USA

Email: karenderocher2018@u.northwestern.edu

OBJECTIVES: We aim to show that human enamel structure and chemistry is similar to that of rodent model systems, and that the intergranular phase region between hydroxyapatite (OHAp) crystallites plays an integral role in determining its susceptibility to etching.

METHODS: Using an artificial sub-surface lesion (ASSL) model, we can simulate the early stages of caries formation *in vitro*. Additionally, exposing sound enamel to solutions containing key trace elements (such as fluorine) prior to etching allows us to investigate the effect of composition on the solubility of the intergranular phase. Atom probe tomography (APT), a technique with sub-nanometer spatial resolution and high chemical sensitivity, allows us to determine the nanostructure and ion distribution of the enamel as a lesion forms and progresses, and lets us visualize the location of trace elements in the overall enamel structure.

RESULTS: Our previous work on rodent enamel established that untreated enamel, where the interphase region has been identified as Mg-substituted amorphous calcium phosphate (Mg-ACP), is mechanically weaker and more susceptible to acid dissolution than pigmented enamel, where the interphase may be comprised of Ca-substituted amorphous ferric phosphate (Ca-AFP). Preliminary experiments show that Mg-ACP also exists in human enamel. In addition, APT analysis shows that fluoride ions rapidly diffuse in Mg-rich ACP, co-localize with Mg^{2+} ions, and slow the dissolution of the amorphous intergranular phase. We will also discuss chemical and structural changes in the interphase and in OHAp nanowires that occur over longer periods of time in an *in vitro* ASSL model.

CONCLUSION: Preliminary results suggest that the dissolution behavior or rat enamel is consistent with that of human enamel. Looking forward, we will analyze the effect of fluoride and iron ions on dissolution and precipitation in enamel. A deep understanding of these processes may eventually lead to better detection and treatment of caries.

DISCUSSION:

Jim Simmer (Michigan): Elia showed in his introduction that when the human ribbons are growing, their cross sectional dimension is like 15 angstroms by 150 angstroms. You are getting the very interesting finding that the core of these crystals, later, is magnesium rich. I'm wondering, so you are taking the cross section of these crystals. What kind of dimension of the area would the magnesium rich (material) be?

Karen DeRocher (Northwestern): You can see here, this is 20 nanometres and so it's probably about 40 nanometres wide, 40 to 50, in this area here.

Jim Simmer (Michigan): 40 by 40 nanometres?

Karen DeRocher (Northwestern): The thickness I guess is probably closer to a couple of nanometres or maybe 5 or so nanometres thick.

Jim Simmer (Michigan): 40 wide?

Karen Derocher (Northwestern): Yes

Colin Robinson (Leeds): We talked earlier in the posters about the fact that what we think is happening is that the enamel crystals are formed by the fusion of a number of sub-units and suggested that at that junction you have a crystalline discontinuity, so in fact it might not be apatite in the sense that it's a perfect apatite. Could you comment on whether or not that could accommodate the amount of magnesium inside the crystal? That, in fact, you've got some kind of discontinuity? The second question is to do with re-crystallisation because, as these units form, magnesium and carbonate will be pushed to the outside, so that's where the interface will be. Your amorphous phase, is it totally separate or part of the surface of the crystal that becomes so disorganised that it looks like a totally separate entity?

Karen DeRocher (Northwestern): I do not know for sure if it is a completely separate phase or if it's just a disordered outer shell around crystallites. I don't have data to say one way or the other. As for the magnesium distribution and your work with the particles coming together, it is believable that maybe, if you do have these particles coming together, that impurities would segregate to the centre as well as the outside, but again we don't really have evidence to support that right now.

Paul Anderson (QMU): Fascinating techniques. As a topographic technique are you able to depth profile some of these elemental distributions?

Karen DeRocher (Northwestern): The lift-outs are taken from the surface to a depth of about 2 microns, but this would have been the surface of the lift out. You can see that we get a depth of about 120 nanometres. If we wanted to get significantly more than that we would probably have to take a lift out from various depths in a different tooth.

Elia Beniash (Pittsburgh): How do you base your localisation of the crystals? Do you have a secondary technique where you actually look at the surface of your cone before mass spectrometry?

Karen DeRocher (Northwestern): We don't, because we're worried that any beam damage to the tip would cause it to not run in the atom probe so, in order to get lift outs from the area we want, what we can do is just a light etching. If we have the sample before and then mark where we want to take our lift out from and then use that as our region of interest that we are going to sample.

Elia Beniash (Pittsburgh): But this isn't a microscope, how do you know that these are crystallites, or is it because of the distribution of different ions?

Karen Derocher (Northwestern): So right now it's mostly based on if the size is correct and the ion distribution makes sense. As I said, we are looking at going into TEM techniques and such to correlate with these results.

Derk Joester (Northwestern): Lyle has done correlative microscopy in rodent enamel where you actually look at the atom probe with TEM, and he was able to align the grain boundaries of individual crystallites on TEM. We haven't done that in human enamel as Karen pointed out.

#60 ENAMEL DISSOLUTION: NOVEL KINETIC METHOD TO MEASURE APPARENT ENAMEL SOLUBILITY

HASSANALI, L[1], LYNCH, R[2], ANDERSON, P[1]*

[1]Dental Physical Sciences Unit, Institute of Dentistry, Barts and The London School of Medicine and Dentistry, QMUL, London, E1 4NS, UK; [2]GSK Oral Healthcare, Weybridge, UK

Email: p.anderson@qmul.ac.uk

OBJECTIVES: Enamel dissolution rate is significantly influenced by its solubility. However, there remains significant debate within the literature about the precise value of enamel solubility. Is it similar to the solubility of hydroxyapatite, or, influenced by chemical inclusions in enamel, or, is there is a range of values? Further, bulk hydroxyapatite itself has deceptive solubility properties, which depend on; surface energy, pH, *etc*. Different methodologies have been described which purport to measure enamel (and /or hydroxyapatite) solubility product (expressed as pKsp), with values are of the order of 110 for enamel, whereas, values of 118-126 have been reported for hydroxyapatite.

METHODS: In this study we report a kinetics method to measure the "apparent" enamel solubility in which scanning microradiography was used to measure the rate of enamel dissolution (RD_{enamel}) as a function of calcium (and phosphate) concentration in the demineralising solution. Enamel slabs were exposed to buffered acetic acid at pH 4.0 for 48 h and RD_{enamel} measured. This was repeated at a series of increasing calcium (and phosphate) concentrations (steps of 3.00 mmol l^{-1} Ca^{2+}) until the demineralisation rate reduced to zero, and then beyond. It is assumed that the concentration at which RD_{enamel} is zero, enamel is in thermodynamic equilibrium with the demineralising solution. A chemical speciation program was used to calculate the calcium concentration at which the degree of saturation is 1 for hydroxyapatite at a range of pKsp values.

RESULTS: RD_{enamel} decreased from 5.22×10^{-4} (SE 6.83×10^{-5}) g cm^{-2} h^{-1} with no calcium, to zero at 31.5 mmol l^{-1} calcium. The calculated solubility product for which a calcium concentration of 31.5 mmol l^{-1} would yield a degree of saturation of 1 was 121.

CONCLUSIONS: The "apparent" solubility product of bulk enamel under caries-like conditions measured using a kinetic method is considerably lower than those previously reported, and much closer to pKsp values reported for hydroxyapatite.

DISCUSSION:

Derk Joester (Northwestern): That was really interesting. I really liked this *in situ* type dissolution. I have a technical question: Do you think, or have any evidence that, the continuous X-ray irradiation might influence the outcomes?

Paul Anderson (QMU): I have no idea. These are X-ray techniques but the X-ray dose is not that high.

Derk Joester (Northwestern): The second is more of a comment. Enamel, even at the individual crystallite level, seems to be very heterogeneous, as Karen has just shown, so your 121 pKsp must be

an average of different kinds of materials, I wonder whether you have any idea of how to see whether this might be locally varying, for example, in teeth.

Paul Anderson (QMU): No, I think you're absolutely right. All the early measurements on the solubility product of enamel, when you see them, where they actually came from, are very hard to interpret. Was the surface behaving as the bulk? [There are] phase transformations on the surface that complicate the issue. What we are aiming at here is to try and see above that, so we get a bulk value, as opposed to a crystallite value which is affected by surface energy, solubility, water complexation at surface (etc). It's a complex issue indeed.

Wendy Shaw (Pacific Northwest National Laboratory): Related to that last question, are you trying to deconvolute the kinds of structures that you are getting there or do you have any way to do that, in terms of is it apatite or ACP, are you able to do that at all?

Paul Anderson (QMU): Not with this technique, no. Essentially what we are trying to get is the coupled, as opposed to the decoupled, value, that is the effective value, but we understand that there's a whole set of other mechanisms beneath.

Henry Margolis (Forsyth): In your Ksp estimations, you assume a stoichiometry of apatite. We know that's not the case. I wonder if you've considered the inclusion of other ions and how they may have an impact determining driving forces?

Paul Anderson (QMU): That's interesting, as people have measured before, including Colin (Robinson). There are gradients in the levels of fluoride, obviously, at the surface, also carbonate and magnesium. The bulk values don't seem to vary that much in terms of the bulk solubility product, but yes, obviously, the individual values are very difficult to put into a speciation programme to obtain the actual solubility product at a single point bearing on the different stoichiometry at those points, but point taken.

#61 HOW TO OBTAIN THE BEST POSSIBLE INFORMATION FROM DENTAL ENAMEL

GERLACH RF[1]

[1]Department of Morphology, Physiology, and Basic Pathology, FORP/USP, University of Sao Paulo at Ribeirao Preto, SP, Brazil

Email: rfgerlach@forp.usp.br

OVERVIEW: Dental enamel is a "rock" that is secreted by cells that die out. Dental enamel is laid down by highly sensitive cells that will register any minor changes in metabolism, shortage of food or diseases. Additionally, enamel accumulates certain metals on its surface to very high levels, such as lead, fluoride and zinc. It becomes more and more evident that the anthropocene is a time with radical changes in our environment. One of the most well-known examples is the huge contamination of the planet with lead. Lead in outer enamel does correlate with the environmental status, as it does with dentine, and blood lead levels.

EVIDENCE: Most data on lead in the enamel could not be understood, because mineralization of the enamel was not understood, and it was mainly treated as a harder dentine. Accumulated evidence of metals on the outer enamel makes sense, and it does also help us understand enamel mineralization. Secondly, very important biomolecules: peptides, were recovered safely from the outer enamel (yes, it does not need to be from the inner enamel), and they allow sexing of mummies, for instance, since the Y-chromosome specific peptides were obtained.

Evidence from the enamel will probably be key to solve such issues as the natural levels of various metals on earth and in human tissues. For determination of natural lead levels, evidence of natural lead levels in the 1970s and 1980s was obtained from ice in Antarctica and the Arctic. Natural lead level results, similar to the ones obtained in ice cores of the poles, can now be obtained from the enamel of contemporary people versus people that lived before lead-smelting had started.

CONCLUSION: Enamel can be now seen as a personal register from our time, from the environment where we grew.

No questions

#62 SUPERFICIAL ENAMEL TRACE ELEMENTS CHANGED OVER TEN THOUSAND YEARS (Pb, Mn, Zn, Cu, Cu, Sr, Cd, Ba, and Ag)

RAMOS, J[1]*, BILAL AS[1], TOLEDO, TCI[1], VEIGA MAMS[2], MARANHAO, TA[3], RAMOS, JC[3], LINE, SRP[4] AND GERLACH, RF[1]

[1]Department of Morphology, Physiology, and Basic Pathology, FORP/USP, University of Sao Paulo at Ribeirao Preto, SP, Brazil; [2]Department of Chemistry, FFCLRP/USP, University of Sao Paulo at Ribeirao Preto, SP, Brazil; [3]Department of Chemistry, Federal University of Santa Catarina, UFSC, Florianopolis, SC, Brazil; [4]Department of Morphology, FOP/UNICAMP, Piracicaba, SP, Brazil

Email: juniaramos@forp.usp.br

Some elements accumulate in the outer enamel of teeth: Pb, F and Mn. It is well-established that a few elements accumulated in the soil and sea as a consequence of anthropic activity, but the impact of this recent accumulation is difficult to study. We hypothesized that the trend towards accumulation in the outer enamel might happen with other elements as well.

OBJECTIVES: This study compares the concentrations of the trace elements Pb, Mn, Zn, Cu, Sr, Cd, Ba, and Ag in the superficial enamel of contemporary teeth with that of ancient teeth, as well as with non-hominine primate teeth.

METHODS: The studied sample included the following groups of teeth: ancient humans: 22 teeth of 13 humans older than 10 thousand years; Non-human primates: a set of 20 teeth of 20 different individuals of several species of non-human primates; Contemporary humans: 23 teeth of 13 individuals. The teeth were submitted to two successive superficial etches using ultrapure diluted HCl for 30 seconds each. The samples were determined by Inductively Coupled Plasma Mass Spectrometry (ICP-MS). Statistical analyses were performed.

RESULTS: The most abundant of the trace elements found was Zn, which accounted for about 3/4 of the total amount of trace elements found, with no significant different between the groups. Pb showed no statistical difference, although 10% of the contemporary humans and the non-human primates had high Pb in the outer enamel. Ba was significant higher in ancient teeth in comparison to contemporary ones ($p = 0.012$), with a 6 X decrease in contemporary humans. A similar trend was found for Mn, which was 10 X higher in ancient teeth ($p = 0.00007$ *versus* contemporary teeth). An inverse trend was observed in Sr levels.

CONCLUSION: Microelements changed over the last 5 thousand years, and superficial enamel may help us to understand how primates' metabolism dealt with the recent changes.

No questions

#76 INVESTIGATING WDR72'S FUNCTION IN PROTEIN REMOVAL DURING ENAMEL MATURATION STAGE

KATSURA, KA*, ZHU, J, NAKANO, Y, ZHANG, Y, AND DENBESTEN, P

School of Dentistry and Department of Oral and Craniofacial Sciences, University of California at San Francisco, San Francisco, CA 94143, US

Email: Kei.Katsura@ucsf.edu

OBJECTIVES: Our overall aim is to determine the function of WDR72 in enamel mineralization. We previously identified a human *WDR72* mutation and characterized a *Wdr72* knockout mouse model, both showing hypomineralized and hypomaturation enamel phenotypes of Amelogenesis Imperfecta. Ours and other published works have demonstrated that loss of WDR72 leads to retained protein in the enamel matrix, which prevents the continuous growth of the enamel crystals. This retention of matrix proteins in the enamel space could be the result of either a defect in matrix protein hydrolysis or a defect in protein removal from the mineralizing matrix. To further direct our studies of the role of WDR72 in enamel formation, we investigated both of these possibilities.

METHODS: Expression of *Klk4* mRNA in *Wdr72*$^{+/+}$, *Wdr72*$^{+/-}$ and *Wdr72*$^{-/-}$ micro-dissected maturation stage ameloblasts was compared by qPCR. Whole mandibular incisors and P14 molars were standardized by weight and demineralized in 0.5 M acetic acid overnight following homogenization. Samples were assayed for KLK4 proteolytic activity using a KLK4-specific quencher peptide and analyzed using a fluorescent plate reader.

To determine the potential role of WDR72 in ameloblast-mediated endocytosis of proteins from the enamel matrix, mice were injected with HRP and relative uptake into *Wdr72*$^{+/+}$ *and Wdr72*$^{-/-}$ maturation stage ameloblasts was compared using confocal microscopy.

RESULTS: *Klk4* mRNA expression and proteinase activity were not significantly different among *Wdr72*$^{+/+}$, *Wdr72*$^{+/-}$ and *Wdr72*$^{-/-}$ mice. HRP uptake was reduced at the apical ends of *Wdr72*$^{-/-}$ maturation-stage ameloblasts, with no obvious effect in secretory ameloblasts.

CONCLUSIONS: Our findings support WDR72 as a regulator of enamel matrix removal and further suggest a role in endocytic processing of enamel matrix proteins. Reduced HRP uptake in *Wdr72*$^{-/-}$ maturation stage ameloblasts, coupled with no significant difference in KLK4 activity, point to WDR72's function in reabsorption of enamel matrix proteins through endocytosis.

DISCUSSION:

Jim Simmer (Michigan): You say that the maturation stage ameloblasts are shorter. That's a difficult conclusion to make in these cells because they get shorter as time progresses and also plane of section can affect the apparent height of the ameloblasts. How did you control for those factors to line up exactly where you were taking your sections and plane of sections? Did you take a lot of sections and take measurements? How did you decide that the ameloblasts were actually shorter?

Kei Katsura (San Francisco): We actually cut a lot of sections but we did it based on, I believe, Charlie Smith and Antonio Nanci's original landmarking based on the molar landmarks. We were

very diligent about demarcating, using the first molar as the mark, as well as the distil portion of the first molar and mesial portion of the second molar.

Jim Simmer (Michigan): So about how much shorter were they?

Kei Katsura (San Francisco): We didn't actually quantify that. It was just a quality observation but it would be interesting to measure it, I suppose.

John Bartlett (Ohio): For the endocytosis, do you know anything about the mechanism of how it may do that, are clathrin-coated pits involved at all?

Kei Katsura (San Francisco): Actually Jim Simmer's group have published that WDR72 seems to associate with clathrin. Typical clathrin proteins involve vesicle trafficking so that would be really interesting to also do co-localisation staining.

PANEL DISCUSSION:

Jason Wan (Bethesda): Dr Gerlach, I enjoyed your talk. A lot of these metals are bound to proteins or there is a sink of proteins in the body that may link to, for example, zinc and lead. How do you think these ions or metals are going into the enamel? Are they bound to proteins and coming into the ameloblasts and then somehow getting out there, or are there active transporters for these metals?

Raquel Gerlach (Sao Paulo): I think it's just like something that happens through precipitation, like what happens with fluoride has also happened with lead and with zinc in the outer enamel. I don't think this is mediated by proteins, or that they (the ions) are bound to proteins.

Mohammed Al-Mosawi (Barts & the London): My question is to Sova et al. Why do you think that mineral concentration is less near the surface, is less than the EDJ in developing enamel, even though it is mineralising the mineralising front? The ameloblasts are closer to the surface at that point in maturation.

Susanna Sova (Helsinki): So still mineralising?

Mohammed Al-Mosawi (Barts & the London): So developing and still mineralising the tooth.

Susanna Sova (Helsinki): Well I see that the mineralising process goes in the same direction as the secretion process and ….

Mohammed Al-Mosawi (Barts & the London): ……but the secretion on the mineralising front actually stays next to the EDJ, so the ameloblasts are next to the EDJ and that's how they produce the enamel matrix proteins, but then at the end, when it goes near the surface of the enamel, do the ions go through the surface of the EDJ and start mineralising from there again?

Raquel Gerlach (Sao Paulo): It's actually very nice what you showed, because those pig results resemble very much the results that Alba published years ago with monkeys (Alba et al. 2010. Proc Biol Sci 227 (1691): 2237-2245). Even in the textbooks with histology you actually have four phases of mineralisation with the enamel. It's well known that it starts from the inner to the upper layers. So it's well known, what she showed resembles the result in monkeys.

Mohammed Al-Mosawi (Barts & the London): I have the same result but I'm just seeing what she thinks. Why is that the reason? Another question for DeRocher-

Elia Beniash (Pittsburgh): May I just comment in the discussion. Basically, in terms of the diffusion, it's really good that it (enamel) does not mineralise at the surface (adjacent to) the ameloblast

otherwise it will never be fully mineralised. We know a little bit more about the secretory phase than the maturation phase and it must be some inhibitors that are distributed throughout enamel in a gradient and this gradient changes over time to allow this mineralisation to happen in this sequential way and we have no clue what they are and how they work and probably somebody here might be interested in looking at this.

Mohammed Al-Mosawi (Barts & the London): Yes. Professor Colin Robinson's idea is that if it mineralises near the surface, it will kind of form a non-permeable layer that ions will not be able to diffuse to the EDJ, but I just wanted to see if you guys have another explanation to that, thank you. DeRocher et al, I think, in my opinion, that there's two ways why we find magnesium and hydroxyapatite or maybe in the other phase. Either in the beginning there's a precursor phase that contains magnesium, like the one that Maisoon Al-Jawad has shown could have magnesium containing whitlokites in AI teeth or it can be at the later stage as an inhibitor to further crystal growth or thickness, I'm not sure about the magnesium having [an effect on the] mechanical properties, can you please elaborate on that?

Karen DeRocher (Northwestern): So that statement was just to say that, because there is so much magnesium incorporation and because it's a smaller ion than calcium, it would cause some strains in the lattice and just seeing how the magnesium substitution affects those lattice strains and seeing if that affects the overall properties of the crystallite.

Derk Joester (Northwestern): I could maybe specify this a little bit more. When you look at these cone shell structures, where you have a magnesium rich core and the magnesium poor periphery of the crystal, then you would expect that the periphery of the crystal is loaded in compression and this is a technique that material scientists use to toughen, for example, your unbreakable dinnerware or the glass that's in your iPhones, because the compression at the surface that's loaded in compression will stop cracks from growing and so it might be an additional toughening mechanism in enamel that hasn't been known before.

Kseniya Shuturminska (QMU): I also have a question regarding the atomic tomography. How do you distinguish the calcium fluoride and other fluorine containing phases, because I saw that you had an outline of calcium fluoride and fluorine in the middle of the crystals.

Karen DeRocher (Northwestern): So in those reconstructions they were just separated based on mass to charge ratio. We fluorinated the sample with sodium fluoride and we believe that the fluoride atoms in the calcium fluoride are in the granular phases due to that treatment, whereas the fluorine inside the crystallites itself was probably native to the tooth before we treated it.

Stephen Hsu (Singapore): I'm just curious about the pigment. Is there any analysis, elemental or chemical analysis, of the pigment? Actually have you found the crystalline structure that caused the result that you have presented?

Derk Joester (Northwestern): So we think that we have fairly good evidence that it's an amorphous ferric phosphate and the structure is not crystalline. If, as I personally think, it is similar to something that is found in soil chemistry, then there is something known about the basic structural units and that will be a ferrihydrite-like core, where we have an iron centre and you have oxygen in tetrahedral arrangement and you have one or two, or maybe three, of these octahedra that are linked but then are phosphate capped and the surface of that is calcium bound. You have a little bit of short range order but no long range order, but the colour is simply the colour from the iron-3 centres in that structure.

Steven Hsu (Singapore): So you are confident that almost no organic matrix is found in the colour?

Derk Joester (Northwestern): For the pigmented enamel we looked at, we saw no evidence of organics. As I recall, we saw traces of organics in the magnesium-rich interface, but as I recall we didn't see any (in the pigmented enamel). I would have to look at that again, but if there was any it was so little that I forgot about it.

Elia Beniash (Pittsburgh): Thank you and anyway there is so little organic in (mature) enamel.

Colin Robinson (Leeds): I would just like to make a comment about the uptake of extraneous ions into the enamel and not get hung up about all the transporting mechanisms which have been talked about which are undoubtedly important. At transition, in early (enamel) maturation, there is clearly a free diffusion pathway. If you look at fluoride, it can go up and it goes down, radioactive phosphate follows radioactive phosphate in the serum so lead, zinc, anything else that goes up will simply diffuse into that stage. It's a very porous stage and a lot of exposed crystallite surfaces, so that's where you going to get a lot of uptake. Equally of course, protons can diffuse out easily.

Raquel Gerlach (Sao Paulo): We have been thinking that maturation stage enamel in the mouth and in different places might be actually a good way to capture what's going on in the environment.

Session 9: Caries

Moderator: Henry Margolis, Forsyth Institute, Boston, USA

#63 BASIC TRANSCRIPTION FACTOR 3 (*BTF3*) GENETIC VARIATION MODULATES ACID-MEDIATED ENAMEL LOSS

VIEIRA, AR

#64 ENAMEL DEFECTS DUE TO MUTATIONS IN PACHYONYCHIA CONGENITA-ASSOCIATED KERATINS

DUVERGER, O*, SHAFFER, JR, CARLSON, JC, MARAZITA, ML and MORASSO, MI

#65 BIODEGRADABLE NANOPARTICLES FOR FLUORESCENCE-AIDED IMAGING OF ACTIVE CARIES LESIONS

JONES, NA*, JANKOVIC, A, SHERBEL, J, TROSKE, WJ, CHANG, S, CLARKSON, BA and LAHANN, J

#66 THERMAL CHARACTERISATION OF ENAMEL AND DENTINE

LANCASTER PE*, BRETTLE DS, CARMICHAEL FA and CLEREHUGH V

#67 VISUALIZING SURFACE ZONES IN RODENT CARIES LESIONS

FREE, RD*, DEROCHER, KA, STOCK, SR, KEANE, DT and JOESTER, D

#68 MULTI-LEVEL MODELLING TO FURTHER UNDERSTANDING OF MICRO-CT DATA

KANG, J*, DAVIES, RP, BROOKES SJ and KIRKHAM J

#63 BASIC TRANSCRIPTION FACTOR 3 (*BTF3*) GENETIC VARIATION MODULATES ACID-MEDIATED ENAMEL LOSS

VIEIRA, AR

School of Dental Medicine, University of Pittsburgh, Pittsburgh, PA, USA

Email: arv11@pitt.edu

OBJECTIVE: We have previously shown that *BTF3* genetic variation, and *BTF3* expression in whole saliva, are associated with caries experience, suggesting that *BTF3* may have functional role in protecting against caries. To further explore these results, we tested if the *BTF3* rs6862039 variant is associated with subclinical dental enamel mineral loss.

METHODS: DNA and enamel samples were obtained from 53 individuals. Enamel samples were analysed for Knoop hardness indentation length of sound enamel, integrated mineral loss after subclinical carious lesion creation, and change in integrated mineral loss after remineralization. DNA samples were genotyped for the rs6862039 marker using TaqMan chemistry. Chi-square and Fisher's exact tests were used to compare individuals above and below the mean enamel microhardness (i.e. mineral loss) of the cohort with alpha of 0.05.

RESULTS: The A allele of *BTF3* rs6862039 appears to be associated with harder enamel at baseline (p= 0.09), enamel more resistant to demineralization (p= 0.01), and enamel that more efficiently reuptake mineral and remineralize (p= 0.04).

CONCLUSION: *BTF3* genetic variation influences the initial subclinical stages of caries lesion formation in the subsurface of enamel.

ACKNOWLEDGEMENTS: Merve Bayram and Figen Seymen (Instanbul University) facilitated DNA and sample collections, Kathleen Deeley (University of Pittsburgh) managed samples and generated genotypes, Frank Lippert (Indiana University) generated enamel microhardness measurements, and Regina Sencak (University of Pittsburgh) statistically analyzed the data.

DISCUSSION:

Henry Margolis (Forsyth): So Alex, I asked you this at your poster about if DMFT [Decayed Missing Filling Index] is not a good screening device, how do you envision screening patients for susceptibility?

Alex Vieira (Pittsburgh): Actually, DMTF has been a very decent device when you try to look at the severity of the diseased populations. The work that we've been doing with DMFT is trying to take advantage of the severity and when you do that analysis, it's not that you're saying to someone, "you are caries-free" and to someone else, "you have a number of lesions, 1 – 32", it actually creates a gradient in severity to analyse it in a more sophisticated way. I still think this is a consequence of the disease and not really how the disease got there. I think, as a paediatric dentist, we know that the best way to look at an individual's susceptibility of future disease, is their having had the disease previously and this is not really a good tool if you want to prevent a disease to begin with. We need a

better way. I don't know the answer, I'm hoping a genomic approach might be helpful but we don't have a good answer for that.

#64 ENAMEL DEFECTS DUE TO MUTATIONS IN PACHYONYCHIA CONGENITA-ASSOCIATED KERATINS

DUVERGER, O[1]*, SHAFFER, JR[2], CARLSON, JC[3], MARAZITA, ML[2 4] and MORASSO, MI[1]

[1]Laboratory of Skin Biology, National Institute of Arthritis and Musculoskeletal and Skin Diseases, National Institutes of Health, Bethesda, MD, USA; [2]Department of Human Genetics, University of Pittsburgh, Pittsburgh, PA, USA; [3]Department of Biostatistics, University of Pittsburgh, Pittsburgh, PA, USA; [4]Center for Craniofacial and Dental Genetics, University of Pittsburgh, Pittsburgh, PA, USA

Email: duvergero@mail.nih.gov

OBJECTIVES: Pachyonychia congenita (PC) is an ectodermal dysplasia primarily characterized by nail dystrophy and painful palmoplantar keratoderma. Additional features of the disease may include oral leukokeratosis, follicular keratosis, cysts, hyperhidrosis, and natal teeth. PC is caused by mutations in *KRT6A*, *KRT6B*, *KRT16*, and *KRT17*, a set of keratin genes known to be expressed in the nail bed, palmoplantar epidermis, oral mucosal epithelium, hair follicle and sweat gland. Despite the natal teeth phenotype observed in some patients with PC, and anecdotal reports suggesting that PC patients may be more susceptible to dental caries, the involvement of this set of keratins in dental development has not been studied.

METHODS AND RESULTS: RNA-seq analysis revealed that *Krt6a*, *Krt6b*, *Krt16* and *Krt17* are all expressed in the mouse enamel organ. We further demonstrated that these keratins are produced by ameloblasts and are all incorporated into mature human enamel where they exhibit distinct distributions. Structural analysis of teeth from patients with PC revealed disruption of enamel rod sheaths, resulting in altered rod shape and distribution. Moreover, using genetic and intraoral examination data from 574 adults and 496 children, we identified several missense polymorphisms in PC-associated keratins that lead to a higher risk for dental caries. Interestingly, these genetic variants are specifically located in regions of the proteins that are adjacent to, but not overlapping with, the regions mutated in PC.

CONCLUSIONS: These results identify a new set of keratins involved in tooth enamel formation, reveal additional clinical features of pachyonychia congenita, and distinguish novel susceptibility loci for tooth decay.

DISCUSSION:

Colin Robinson (Leeds): Could you tell me how soluble these things are? The reason I ask is that there's evidence that in the dark or positively birefringent zone of caries, which produces a lot of very small holes as well as larger ones, which develop because of continued crystal dissolution, that some of them are blocked up with organic material. I'm wondering if these things are released; do they move and re-precipitate maybe?

Oliver Duverger (Bethesda): No, they are extremely tough proteins that are almost indestructible and that's why, during the process of maturation, when they are part of the initial matrix, they are the only components that will not be degraded by all of the proteases involved in maturation. So we see it really as a mesh that remains within the crystals at the end of the maturation stage.

Sherif Elsharkawy (QMU): So, these keratins are really abundant at the DEJ, is that true?

Oliver Duverger (Bethesda): Yes

Sherif Elsharkawy (QMU): So you've said that their effect is to decrease caries, how can that location help in decreasing the extent of caries?

Oliver Duverger (Bethesda): I showed that keratin 6 is also strongly (present) on the DEJ. These keratins actually propagate throughout the thickness of the enamel in the very thin layer of the enamel rod sheath, so if there is an acid attack at the surface of the tooth these will be protective of the rods. If the stability of the enamel rod sheath is affected by the mutation of these keratins, it will make the progression of the carious lesion faster and more severe.

Sherif Elsharkawy (QMU): So the degradation happens more at the top surface. It's not easy to degrade them at the DEJ, is that helping the integration between the enamel and dentine?

Oliver Duverger (Bethesda): If there is a mutation that destabilizes those keratins it will make it easier to degrade throughout the enamel thickness. But, I thought your question was, are keratins also present at the surface to affect the initiation of the lesion formation? As soon as the initiation happens, what we showed in the keratin 75 story is that it seems to be that we have an easier progression through the thickness of the enamel, and we had very characteristic tubular carious lesions that were not visible on X-ray but only in high-resolution micro-CT , following bunches of rods throughout the thickness all the way to the dentine.

Elia Beniash (Pittsburgh): Even if these keratins don't go all the way through the surface, definitely they affect the enamel rod shape and most likely they affect the mechanical properties of enamel. If there is a lot of micro-cracking, they will assist bacterial invasion into the enamel as well.

#65 BIODEGRADABLE NANOPARTICLES FOR FLUORESCENCE-AIDED IMAGING OF ACTIVE CARIES LESIONS

JONES, NA[1]*, JANKOVIC, A[2], SHERBEL, J[2], TROSKE, WJ[3], CHANG, S[2], CLARKSON, BA[2] and LAHANN, J[1 3]

[1]Macromolecular Science and Engineering, the University of Michigan, Ann Arbor, 48109, MI, USA; [2]School of Dentistry, the University of Michigan, Ann Arbor, 48109, MI, USA; [3]Chemical Engineering, the University of Michigan, Ann Arbor, 48109, MI, USA

Email: najones@umich.edu

OBJECTIVES: Understanding the pathological changes of carious enamel can allow for rational design of targeted nanoparticles for improved imaging and diagnosis. We applied Scanning Probe Microsopy (SPM) to early carious lesions to characterize the enamel structure and surface properties. We then used these results to design a novel nanomaterial which can: target and adhere to active caries lesions by sub-micron size and cationic charge; fluoresce when illuminated by a dental curing light; and degrade in the oral cavity into non-toxic compounds. This nanoparticle offers a new targeted imaging probe useful for studying the architecture of carious enamel.

METHODS: We measured the roughness and surface potential of active and inactive carious lesions using SPM. We then prepared and characterized fluorescently-labeled starch nanoparticles, validated their degradation in saliva, and observed specific illumination of active caries lesions, both macroscopically, using computer image analysis, and microscopically, using two-photon microscopy.

RESULTS: SPM validated sub-micron porosity and negative surface charge of active carious lesions. Prepared nanoparticles were fluorescent, cationic (+5.8 ± 1.2 mV), degradable in saliva, and able to penetrate early caries lesions (101 ± 56 nm). Upon illumination with a dental curing light, these nanoparticles specifically illuminated active caries relative to anionic and uncharged controls (student's t-test, n=15, $p< 10^{-5}$), but did not illuminate remineralized (inactive) caries. Optical contrast was further improved using computer-aided image analysis to extract green-pixel images (student's t-test, n=15, $p< 10^{-6}$). Two-photon microscopy showed illumination of microscopic pores and may offer insight into the porous architecture of caries lesions.

CONCLUSIONS: Based on SPM studies, we were able to design nanoparticles that offer a new fluorescent probe for detecting and differentiating between active and inactive carious lesions on macroscopic and microscopic scales. In particular, two-photon microscopy of lesions illuminated by these particles highlight the porous enamel architecture of active caries.

DISCUSSION:

Unknown: Did you compare your method with the QLF or the Diagnodent or others?

Nathan Jones (Michigan): We haven't compared directly head-to-head with those. I think the key advantage of this method is that it can distinguish between active and inactive lesions, which has been a drawback with a lot of the technologies like Diagnodent. At the same time, the starch particles used in our method are fairly cheap to produce, so it may offer a cheaper alternative, but we would have to scale it up first.

Henry Margolis (Forsyth): How do you go about, if you are a clinician, finding these areas to apply this, or do you envision a whole-mouth application?

Nathan Jones (Michigan): I think there's potential for both, so in initial testing, it might depend on the cost point for the dentists as to what they would want to do. It could be applied to regions that look suspicious. If you see a white spot lesion and you're not sure if its active or inactive you can distinguish between those, but if you just apply it as a general rinse, then you might be able to see all of these lesions much more clearly in the mouth.

#66 THERMAL CHARACTERISATION OF ENAMEL AND DENTINE

LANCASTER PE[1]*, BRETTLE DS[2], CARMICHAEL FA[3] and CLEREHUGH V[1]

[1]School of Dentistry, University of Leeds, Leeds, LS2 9LU, UK; [2]Department of Medical Physics and Engineering, St. James's University Hospital, Leeds, UK; [3]Department of Dental and Maxillofacial Radiology, Leeds Dental School, UK

Email: p.e.lancaster@leeds.ac.uk

The mineralised tissues of a tooth have their own specific thermal signatures, as seen with thermal diffusivity and conductivity, which provides the opportunity to visually characterise tooth enamel and dentine. Variation in the homogeneity of these tissues may also be detected by associated changes in these thermal properties, leading to a diagnostic potential which is currently unused within the field of dentistry.

OBJECTIVES: The primary aim of this study was to visually characterise sound enamel and dentine from data collected with an infrared thermal imaging camera, from the unique perspective of thermal characteristic-time and heat-transfer rate. The secondary aim was to view carious enamel and dentine for diagnostic potential of this novel imaging technique.

METHODS: Two human molar teeth, one sound and one carious, were sliced bucco-lingually into 1 mm thick samples with an Accutom-5. Each slice was cooled and rewarmed in a stable macro- and micro-temperature-controlled aluminium thermal chamber. A FLIR SC305 Infrared Thermal Imaging Camera recorded the heat-transfer-sequence. Bespoke software processed the thermal data to generate unique thermal maps from the characteristic-time and heat-transfer rate for both sound and carious enamel and dentine.

RESULTS: The software successfully generated the first characteristic-time-maps and heat-transfer-rate-maps, which effectively differentiated enamel from dentine. Each of the maps also distinctly demarcated the areas of the carious lesion within enamel and dentine. The heat-transfer-rate-maps were produced in less than a second.

CONCLUSIONS: This stable thermal environment enabled examination of teeth-slices with an infrared thermal imaging camera, which is non-destructive, non-invasive, and non-ionising. The bespoke software undoubtedly identifies sound enamel and dentine in the novel thermal-maps. In addition, the carious lesion is clearly defined, showing promise as a future diagnostic tool.

DISCUSSION:

Iris Frasher (Munich): Could the same technique be used to identify the difference between dentine and the pulp and could it be used for example, for trauma patients or for identifying a difference in vital or non-vital pulp?

Paula Lancaster (Leeds): Yes, that is exactly what I'm working on at the minute. I've got a whole tooth model because these data presented here are obviously just slices of the teeth. The infrared is very much a surface effect but trauma to the tooth might lead to de-vitalisation with a reduction in blood flow, which will affect the temperature. So at the minute, with my tooth model, which is a little

bit unique, we have positive visualisation of re-warming with a flow rate of 0.5 mm, so I'm currently dropping the flow rate. There's a lot of controversy about what the actual flow rate of blood is into a tooth, so at 0.5 we've got equilibrium, which is what we would expect in the tooth to start with from the vitality point of view. People have done the vitality test previously and they've come to the conclusion that, no they can't measure it. With the improvement in the technology we have, certainly with the images that we're managing to generate from the maps, then yes, we can actually show differences. I can show you some images later on.

Sherif Elsharkawy (QMU): Great idea, great technique. What I'm wondering is about the thickness of the whole bulk tooth, because your data are from sections of around 1 mm thickness. How would the heat transfer be in the actual tooth? This is one thing. Another thing, what about the soft tissue? We had a discussion about the soft tissue and how it will transfer some heat that will overlap with whatever you have underneath.

Paula Lancaster (Leeds): Certainly the soft tissue is claimed to transfer some heat into the tooth. What I'm doing with the slices is actually cooling the slices down and then watching the tooth rewarm, which we can do in our human volunteers quite nicely, compared to actually heating the teeth. So the amount of heat that's transferred from the periodontal ligament and surrounding soft tissues is debatable. The early research by Shapiro said that transferred heat actually overpowered what was being contributed from the vital supply. Now, the information that I'm getting from what I'm doing currently, from the vitality point of view, is that that's not the case. We can actually pick up what's going on, so if that follows through, we should be able to see the demineralisation on the surface of the teeth. From the whole structure of the tooth, you're quite right, I haven't got slices there so the information that we've got, we've treated as a 1-dimensional heat transfer. In the full bulk of the tooth we're going to have 3-dimensional heat transfer. The infrared imaging technique is a surface technique. If we've got a change in the mineral content of the tooth tissue going through and you get the spreading along the ADJ for demineralisation, it would be hoped that, that change in transfer coming through, would be picked up on the surface.

Sherif Elsharkawy (QMU): Would you need 360 degree camera?

Paula Lancaster (Leeds): No, not at all. You could cool the slice down, you could cool the tooth down, the whole tooth *in situ* and the area of your interest. You could then watch the re-warming. Now, whether the re-warming purely comes from the pulp tissue, or whether the re-warming comes from the periodontal ligament, I don't actually mind so as long as we get re-warming and then we can see the different rate of change of the thermal conduction through the tooth.

#67 VISUALIZING SURFACE ZONES IN RODENT CARIES LESIONS

FREE, RD*[1], DEROCHER, KA[1], STOCK, SR[1], KEANE, DT[2] and JOESTER, D[1]

[1]Materials Science and Engineering, Northwestern University, Evanston, IL, 60208, USA; [2]DND-CAT, Advanced Photon Source, Argonne National Lab, Lemont, IL, 60439, USA

Email: RFree@u.northwestern.edu

OBJECTIVES: We aim to demonstrate that intact surface zones, a well-known and important feature of human enamel caries, also manifest during rodent caries.

METHODS: Carious lesions were generated in rats through established protocols that combine a sugar-rich diet and water with cariogenic *Streptococcus mutans* inoculations.

Synchrotron X-ray computed microtomography (μ-CT) with a 6 μm voxel resolution was performed on whole extracted M1 molars, yielding complete three-dimensional reconstructions of relative mineral density in a non-destructive manner. Z-slices of reconstructions were analyzed with ImageJ to identify lesions, quantify demineralization, and characterize intact surface zones with depth profiles.

RESULTS: A total of 130 lesions were identified across 30 scanned samples, and 95 (73 %) displayed intact surface zones. Surface zones were observed in 12 biological replicates, in multiple teeth from the same animal, and in multiple lesions within the same tooth. While the precise 'age' of any particular lesion is not known, the number and severity of lesions were generally much greater in molars from rats sacrificed at 49 days of age versus 35. At this later time point, many regions of demineralization had coalesced and the most severe lesions had cavitated, especially in sulcal regions. Considering that surface zones were observed in both mild and severe lesions from both time points, it is likely that the surface zone exists for a large fraction of any individual lesion's lifetime.

CONCLUSION: For the first time, we present definitive evidence that surface zones exist during rodent enamel caries. This confirmation supports the use of such animal models as a human analog to explore disease pathology and evaluate potential treatments. We next seek to leverage non-destructive imaging by μ-CT to target lesion zones for characterization via atom probe tomography.

DISCUSSION:

Colin Robinson (Leeds): It's a comment really, but I think it's in support of this. It's to mention the work that Alan Hallsworth did when he was dissecting translucent zones of lesions. They had only lost about 0.5 - 1 % of mineral. When he calculated the composition of what was lost, it couldn't conceivably have been apatite. It was very calcium carbonate and magnesium-rich and we can only assume, looking at your data, that's what that first zone is. It is the removal of what we thought may be just crystal surfaces and it fits quite nicely, I think.

Henry Margolis (Forsyth): Was that rodent enamel Dr. Robinson?

Colin Robinson (Leeds): Human natural caries.

Janet Moradian-Oldak (Southern California): Just a simple question, why didn't you start just creating those lesions in human extracted molars? Why use rodent teeth?

Robert Free (Northwestern): So we had a parallel study going on. Karen, my colleague, is working to create those sub-surface lesions in human extracted molars, but this, we wanted to sort of do in parallel with an *in vivo* caries model to see if we could first look at those amorphous intergranular phases in the actual full case. We are sort of coming at it from both sides; I'm on the rodent side.

Henry Margolis (Forsyth): Are there any limitations in working with a rodent model versus working with human extracted teeth?

Robert Free (Northwestern): I think that there are always limitations with the animal models. The underlying structures are slightly different in rodent enamel, but the fact that they do show this sub-surface structure I think further strengthens their use for particular studies and if you're interested in a surface zone effect, maybe a rat model is a good place to start, where you have a bit more control over the environment and what you're introducing.

#68 MULTI-LEVEL MODELLING TO FURTHER UNDERSTANDING OF MICRO-CT DATA

KANG, J*[1], DAVIES, RP[1], BROOKES SJ[1] and KIRKHAM J[1]

[1]School of Dentistry, University of Leeds, Leeds, LS2 9LU, UK

Email: j.kang@leeds.ac.uk

X-ray micro-computerised tomography ("micro-CT") is an important tool in enamel research but evaluating interventions based on "before and after" reconstructed image stacks is complex and use of over-simplified statistical tools may mask potentially important relationships.

OBJECTIVES: To compare and contrast outcomes obtained using multi-level modelling of data with simple statistical analyses for micro-CT data evaluation in a series of remineralisation experiments.

METHODS: Datasets were obtained from experiments evaluating the effects of 9 different interventions (a range of self-assembling peptides designed to nucleate hydroxyapatite, fluoride varnish and water control) on the remineralisation of artificial lesions in human enamel. Two to six lesions were generated per tooth and the mineral density of the whole lesion volume quantified from calibrated micro-CT image stacks. Lesion mineral density was quantified again following a given intervention and subsequent remineralisation for 7 days. Data was evaluated using either 1) ANOVA; 2) non-parametric Kruskal-Wallis test with post-hoc comparison or 3) multi-level modelling.

RESULTS: Simple statistical analyses, such as ANOVA or non-parametric Kruskal-Wallis test with post-hoc comparison, underestimated the effect of an intervention by ignoring the association between lesions from the same tooth and the heterogeneity across individual teeth. Multi-level modelling was able to consider the clustering effect among lesions within a single tooth and the baseline values of lesion mineral density; providing the correct effect size by considering hierarchical levels of lesion samples and taking in to account the different responses of each individual tooth under treatment.

CONCLUSION: Data generated by studies such as this should be analysed using appropriate statistical methods that consider the hierarchical nature of sample populations and heterogeneity existing amongst them. Simple statistical analyses could underestimate the effect of a given intervention, potentially masking significant findings and reducing the potential impact and reach of the research.

<u>DISCUSSION:</u>

Henry Margolis (Forsyth): I was wondering how far one takes this? You go from the slab, to the tooth, to the mouth, to the individual, I mean you're adding more and more parameters.

Jing Kang (Leeds): Here, in the model used, the parameter we have is actually just the treatment and the initial condition (with respect to lesion demineralisation at baseline). This is an established statistical model and there is some very nice software available that can be used to do the analysis.

Once we have the data, you specify how many levels the data is structured across and what is the parameter that we want to investigate and the result can be obtained quite quickly.

Henry Margolis (Forsyth): Is the need for such multi-level modelling due to the heterogeneity of tooth enamel? So this is an improved method where you have taken into account that you have taken sections from the same tooth. I'm wondering if that's because teeth are so heterogeneous between individuals and even within the same tooth. Is that true?

Jing Kang (Leeds): Yes, the tooth is very heterogeneous. It's just like human beings, each person is so different and it's the same teeth, teeth from different people are quite different as well.

Derk Joester (Northwestern): You have me intrigued, you have come up with a ranking in this procedure but you haven't really told us how much better D is than A. Can you give us an idea?

Jing Kang (Leeds): Yes, you're definitely correct, we can rank them, but some of the ranking does not reflect statistically significant differences between treatment groups. The only significantly different group we found is actually treatment group H, which is far, far, worse than the rest. Taking into account the parameter estimation, we can still make a ranking but we cannot conclude that D is definitely better than A.

<u>PANEL DISCUSSION:</u>

Brian Clarkson (Michigan): On the thermal imaging, just a comment: you probably couldn't distinguish between active and inactive lesions using that method. How do you distinguish between hypocalcification or hypomineralised spots on teeth and caries?

Paula Lancaster (Leeds): The only information that we have at the minute are the images that you see with respect to the demineralisation. Now we hope to be able to quantify the actual data. For the hypo- or hyper- quantification, we may have a different thermal signature for each of these to be able to investigate that might lead to some identification of what's going on. At the minute I can't say that for sure but it's an area for us to look at.

Stephen Hsu (Singapore): Just a follow-up question for Dr Kang, for the new multi-level analysis method, were these results better than traditional ANOVA or Kruskal-Wallis and can you show any major difference between these two?

Jing Kang (Leeds): Actually, I wouldn't say the multi-level model can give you a "better" result because that depends on how you define a good result. If we take finding a significant difference between multiple treatment groups as a good result then fine, but if we used the wrong approach to obtain that result then that isn't correct. I would say, use a multi-level model to take into account the structure of the data. For example, if we consider that in our case, several enamel slabs containing lesions were obtained from the same tooth, we need to control for this and estimating the correct standard error among our data will enable us to conclude the correct result. As I showed earlier, if we only look at the lesions as if they were independent of one another, ignoring which tooth those slabs are coming from, then we would get many significant differences between the treatment groups but many of them are false-positives. A lot of false-positive results might look good but I wouldn't say that that's a good scientific result to present to the general public without the proper statistical analysis.

Stephen Hsu (Singapore): Just a quick verification, the good results are the ones closer to the truth and the truth that we know, and we are just curious about what's the difference between these two. And, also, the site specificity in enamel is so huge, the buccal enamel and the lingual enamel of two different teeth maybe closer in composition than the difference between the lingual side of two teeth adjacent to each other, so that was my question.

Jing Kang (Leeds): I definitely agree with you, but unfortunately in this experiment we have no idea where the tooth came from because I believe they were all obtained from the tissue bank. If we had this additional information then we can put the data into our model and consider such information in our data analysis. We can't consider that at the moment but thank you for your suggestion. The comment regarding the result you mentioned, as I demonstrated earlier, if we look at the tooth as a whole, by averaging data from enamel slabs taken from a single tooth, we lose a lot of information and this will give us a lot of false negative results. If we look at the enamel slabs as if they were

independent, ignoring which tooth they come from, we will have a lot of false positive results. A multi-level model combines these to give us what we believe is a true significant difference between the treatments by taking in to account all associations and errors at each level.

Derk Joester (Northwestern): I have two quick questions, one for Alex Vieira. So, as Henry Margolis mentioned in his introduction, caries is a really multi-factorial disease. In your analysis, how were you able to control for the many, many things, from nutrition to diet to environmental factors, that might confound the analysis of genetic pre-disposition?

Alex Vieira (Pittsburgh): Well, that's a very good question, the criticism is well-taken. So there are two different things that I hinted at. One is that in the original association studies with humans, where those factors actually are much more dramatic and impact the data, we did the best we could. We had surrogates of socioeconomic status. The main experiment was done in the Philippines, so these people are coming from the same geographic area, they had the same cultural influences, kinds of diets and, in general, were living in kind of the same socioeconomic strata, so we could make a general assumption that the diet looked similar and so on and so forth. We got a sense that it's not perfect by far, but it's the best we could do. We did not have microbiological data. The latest studies we've been doing are adding that because technology is allowing us to afford that, but we did not have that in previous work and so if there are differences in the bacterial colonization then we cannot take that into account. In the case of the later experiment, which was done in extracted teeth, with a corresponding DNA sample from the same individuals, those individuals all had lower caries experience and they are about the same age, there were a number of males and females and then the lesion was created artificially in the lab. So, in that sense, there is a little bit less variability, but of course we are one step removed from what the reality is. But your criticism was well taken and I'm mindful of this; it's just the challenge we have.

Derk Joester (Northwestern): It wasn't meant as a criticism. My next question is a question for the thermal imaging. So, not having a lot of experience with the techniques that have been used, could you maybe briefly compare with the available imaging techniques? I heard your technique, and as a chemist my instinct would probably be to go to near infrared imaging for such applications. Just for us beginners, could you briefly comment on what's possible with which technique?

Paula Lancaster (Leeds): Yes, I could probably fill you in with the thermal imaging. The situation that we have at the minute is that there is no intra-oral camera, and the actual devices we have to enable us to image the anterior teeth are quite large, so you can only view the anterior teeth. When you are looking at the resolution capabilities, we have limited resolution visibility within the infrared. I'm in the mid-range with the camera that I'm using so I'm 7.5 to 13 microns and with a x4 lens we've got a 100 micron resolution with the images that we've got. So that's the position that we are at with the thermal camera. There are complications with the thermal imaging as well. We had emissivity aspects to consider when looking in the fissures. Previous research has said that they've detected caries within the fissures, but no consideration has been given to the emissivity problems within the fissure area. So, I'm very much focussing on a smooth surface area, which is much easier to be able to reduce that confounder. There is an emissivity difference between dentine and enamel but I'm looking at the difference in the temperature, rather than the absolute temperature. I'm not

saying this is going to be the be-all and end-all, but from a thermal point of view, with the way in which the technology is progressing, I think we've got potential for screening. With the camera technology now, we can attach a thermal imager to an iPhone and we've got infrared images that are the size that will go into pockets. So from a public health point of view, that's the direction that I'm coming from. Comparing with the other images, I'm probably not the best person to be able to comment too much on that, so I'm going to pass on that one.

Derk Joester (Northwestern): I like the idea of having thermal selfies!

Alex Vieira (Pittsburgh): The first question is for everybody. If you can suggest a way to look at a transcription factor that might be impacting the development of enamel in a particular moment, I would be very happy to hear your ideas and learn from your insight. I have a question for Nathan. From your perception in the clinic, how hard is it to distinguish an active from an inactive lesion? What's happening with caries is that caries has changed in the last 5 decades and now, because of widespread fluoride exposures, those lesions are smaller and they are trickier to identify. I understand your motivation, is that something that you feel is an obvious need for the clinician?

Nathan Jones (Michigan): Yes, so that was something I have actually been involved with in an NSF I-Corps (National Science Foundation Innovation Corps) program right now. I've been interviewing a lot of dentists to get their experience with this. Really, what they've told us is that, when it comes to telling the difference between active and inactive lesions, it's a guess, especially if they have met the patient for the first time. Typically, what they'll do is follow up with a patient regularly, and then yes, they can see that there's progression over time and that is how they would measure activity. But really there is no good way and that was a key motivation for us in this project.

Paul Anderson (QMU): I'm still confused as to why we get subsurface demineralisation in enamel. Do you think the rat model is going to give us any more information as to understanding why, when enamel dissolves, you get subsurface demineralisation rather than what you'd intuitively think, i.e. that the surface will dissolve first. It's not a straight physico-chemical effect.

Robert Free (Northwestern): I think you could definitely help with that. You can easily use these subsurface lesions and create a large number of them in a controlled environment. We can generate that in something that's similar to humans that can serve as an analogue. I think that will open up a potential for a wider variety of experiments to give us more information down that path. So I think it can help. I don't know if it will definitively provide the answer immediately, but I think, as a tool, it's good to know that it at least exists and can serve as a model.

Stephen Hsu (Singapore): I ask this question on behalf of paediatric dentists who are curious about your really nice work on keratin, as there is a distinct difference between the enamel matrix in primary teeth and adult teeth. We know that the caries progression is a lot faster in primary/baby teeth, which have a greater organic component than that of adult teeth. Have you seen a major difference in keratin distribution, structure, and function between adult and baby teeth?

Olivier Duverger (Bethesda): We have not looked at primary teeth in terms of distribution of these keratins. But clearly, we had this example also for keratin 75, where we had looked at 2 different hair disorders. One SNP in pseudofolliculitis barbae is leading to in-grown hair in regions of shaving in an adult (obviously), and the other SNP leading to loose anagen hair syndrome, which is a syndrome in which children have hair that is easily plucked and when they grow older they don't have any hair problems anymore. It turned out that the SNP that was linked to pseudofolliculitis barbae was leading to higher risk of caries in adults only and not in children, while the SNP linked to loose anagen hair syndrome had the opposite association. For keratin 6, we have the same situation, with two SNPs in the same keratin leading to risk in adults or in children. I didn't have time to mention this but we also find a link between these two SNPs in the sense that adults who have the SNP that leads to higher risk in adults have an even higher risk if they are also carrying the child-associated SNP but the other way around it's not true. So, when you have the SNP that leads to higher risk of caries in children, it leads to an exacerbated effect of the second SNP that leads to higher caries risk in adult. But you're right, our feeling is that these are expressed in both primary and permanent teeth, but probably the way they are incorporated into the matrix is different in the two sets of teeth, but we have not looked at this in detail.

Session 10: Enamel Matrix Proteins

Moderator: Steven Brookes, University of Leeds, UK

#69 AMELOGENIN - AMELOBLASTIN INTERMOLECULAR COMPLEXES AT THE ENAMEL PRISM BOUNDARY

MAZUMDER, P, SU, J, BAPAT, R, PRAJAPATI, S AND MORADIAN-OLDAK, J*

#70 KLK4 BINDS HYDROXYAPATITE AND UNDERGOES AUTOLYSIS IN ABSENCE OF SUBSTRATE

PEREZ, VA*, MANGUM, JE and HUBBARD, MJ

#71 THE STRUCTURE OF AMELOGENIN MUTANTS IN SOLUTION AND ON HYDROXYAPATITE

SHAW, WJ*, ARACHCHIGE, R, BURTON, SD, LU, J, XU, YS, TAO, J, TARASEVICH, B and BUCHKO, G

#73 PHOSPHORYLATION INFLUENCES THE SECONDARY STRUCTURE OF THE LEUCINE-RICH AMELOGENIN PEPTIDE

YAMAZAKI, H*, BENIASH, E, YAMAKOSHI, Y, SIMMER, JP, and MARGOLIS, HC

#74 AMELOGENIN PHOSPHORYLATION IS ESSENTIAL FOR TOOTH ENAMEL FORMATION

SHIN, N-Y, YAMAZAKI, H, MARGOLIS, SS, PUGACH, MK, SIMMER, JP[5], BENIASH, E[6] and MARGOLIS, HC[1 2]*

#75 ARE INTERACTIONS BETWEEN JUNCTIONAL EPITHELIUM PROTEINS KEY TO DENTOGINGIVAL ATTACHMENT?

HOLCROFT, J, SAN MIGUEL, S, GANSS, B*

#76 INVESTIGATING WDR72'S FUNCTION IN PROTEIN REMOVAL DURING ENAMEL MATURATION STAGE

KATSURA, KA*, ZHU, J, NAKANO, Y, ZHANG, Y, AND DEN BESTEN, P

#69 AMELOGENIN - AMELOBLASTIN INTERMOLECULAR COMPLEXES AT THE ENAMEL PRISM BOUNDARY

MAZUMDER, P, SU, J, BAPAT, R, PRAJAPATI, S AND MORADIAN-OLDAK, J*

Center for Craniofacial Molecular Biology, Division of Biomedical Sciences, Herman Ostrow School of Dentistry, University of Southern California (USC), Los Angeles, CA, 90033, USA

E-mail: joldak@usc.edu

Recent *in vivo* and *in vitro* studies provide supporting evidence that extracellular matrix proteins intermolecular complexes may play crucial roles in the process of crystal nucleation, growth and enamel tissue organization [Front. Physiol, 5 (2014) 274, J Struct Biol, 183 (2013) 239-249].

OBJECTIVES: To investigate ameloblastin and amelogenin interactions *in vitro* and to demonstrate their spatial co-localization in postnatal 1st mandibular mouse molars.

METHODS: We used *in situ* immunofluorescence imaging; immunochemistry, quantitative co-localization analysis (QCA) and new fluorescence resonance energy transfer (FRET) technique to demonstrate ameloblastin and amelogenin spatial interactions *in situ*. To identify interacting domains *in vitro* we designed four ameloblastin peptides derived from different regions of the full-length protein (AB1, AB2 and AB3 at N-terminus, and AB6 at C-terminus) and studied their interactions with a recombinant full-length amelogenin (rP172), and a synthetic tyrosine-rich amelogenin polypeptide (TRAP). A series of amelogenin Trp (W) variants (rP172 (W25), rP172 (W45) and rP172 (W161)) were also used for intrinsic fluorescence spectroscopy and circular dichroism.

RESULTS: The confocal laser microscopy and co-localization analysis of doubly labeled transverse sections at P8 showed that N-terminal ameloblastin and N-terminal amelogenin fragments co-localized around the periphery of the enamel rods in maturing rodent enamel. FRET analysis confirmed their molecular interactions in the nanometer range *in situ*. Fluorescence spectra of rP172 titrated with AB3 showed a shift in λ_{max} in a dose-dependent manner, indicating molecular interactions in the region encoded by exon 5 of ameloblastin. Fluorescence spectra of amelogenin Trp variants, as well as the spectra of TRAP titrated with AB3, showed that the N-terminus of amelogenin is involved in the interaction between ameloblastin and amelogenin.

CONCLUSION: Our data support cooperative functions of enamel matrix proteins in mediating the structural hierarchy of enamel. We suggest that macromolecular co-assembly between amelogenin and ameloblastin may play important roles in enamel biomineralization.

ACKNOWLEDGEMENTS: Supported by NIH-NIDCR; DE-13414, DE-020099.

DISCUSSION:

Colin Robinson (Leeds): When these enamel protein complexes form they are right across the enamel. Do you think the complexes function in actually creating the prism or do they simply end up there after completing some other function when they’ve been processed and then complexed?

Janet Moradian-Oldak (Southern California): Based on what we know about the secretory pathway of these proteins, there is some secretory activity on the lateral side of the ameloblast and the ameloblasts are responsible for forming the prisms. I think that fragments are localised at the prism periphery to maintain prism integrity rather than prism formation, ensuring prisms do not collapse or fuse with the interprismatic enamel.

Colin Robinson (Leeds): Does this stop the crystals going across the prism boundary?

Janet Moradian-Oldak (Southern California): That's the idea.

#70 KLK4 BINDS HYDROXYAPATITE AND UNDERGOES AUTOLYSIS IN ABSENCE OF SUBSTRATE

PEREZ, VA[1 3]*, MANGUM, JE[1] and HUBBARD, MJ[1 2]

[1]Department of Pharmacology and Therapeutics, University of Melbourne, Australia; [2]Department of Paediatrics, University of Melbourne, Melbourne, Australia; [3]School of Dentistry, University of Talca, Talca, Chile

Email: vperez@utalca.cl

BACKGROUND: Better understanding of the mechanistic basis of enamel hardening may have biological, pathological, and bioengineering benefits. The protease KLK4 degrades amelogenin (AMEL) at early- to mid-maturation as prelude to the hypermineralisation and final hardening of enamel matrix. It has been suggested that KLK4 may then be eliminated by paracellular diffusion after AMEL degradation, given its absence from mature enamel and reported lack of hydroxyapatite-binding properties. However, our recent finding of KLK4 in hypomineralised (chalky) enamel seems to challenge this idea, raising questions about the mechanism of KLK4's removal from the matrix.

OBJECTIVES: Test the hypothesis that 1) native KLK4 is a hydroxyapatite-binding protein and 2) KLK4 undergoes autolysis in absence of AMEL substrate.

METHODS: To test mineral binding, pure recombinant KLK4 (100 ng) was incubated with powdered hydroxyapatite (100 mg) at room temperature in physiological saline, then bound/unbound fractions were analysed with SDS-PAGE and immunoblotting. To test for autolysis, KLK4 was incubated without substrate for up to 48 h and its structural integrity was assessed as above.

RESULTS: KLK4 bound quantitatively to hydroxyapatite under native conditions with rapid kinetics ($t_{1/2}$ < 1 minute). In absence of substrate, KLK4 remained as a stable monomer after 1 h incubation, but became progressively degraded after 6 hours of incubation.

CONCLUSION: Based on these *in vitro* findings, after degrading AMEL, KLK4 has potential, both to be retained by enamel mineral and to undergo autolysis. Given the absence of KLK4 from mature enamel, we speculate the latter may occur during normal enamel development. These findings differ from an earlier report suggesting diffusional elimination of KLK4, perhaps due to their use of non-native conditions.

DISCUSSION

Steve Brookes (Leeds): Did you look at KLK4 activity when it was bound to hydroxyapatite?

Vidal Perez (Melbourne): No. It's an experiment we would have to think about.

Jason Wan (Bethesda): Is there any autoproteolysis inherent in the KLK4 protein on secretion into the matrix, or does it have to wait until all the matrix proteins are degraded before becoming autoproteolytic?

Vidal Perez (Melbourne): We think that when substrate is present, KLK4 degrades amelogenin in maturation.

Mike Hubbard (Melbourne): We don't expect KLK4 to autodegrade because there's substrate present. Using Triton as a proxy substrate keeps the KLK4 molecules apart in the absence of amelogenin and prevents their degradation.

Jim Simmer (Michigan): Do you know if autoproteolysis inactivates KLK4? KLK4 has 6 disulphate bridges and cleaving the backbone might not cause loss of activity.

Vidal Perez (Melbourne): I'm not sure. We think that if KLK4 undergoes autoproteolysis it should lead to some level of inactivation of the protein.

#71 THE STRUCTURE OF AMELOGENIN MUTANTS IN SOLUTION AND ON HYDROXYAPATITE

SHAW, WJ*, ARACHCHIGE, R, BURTON, SD, LU, J, XU, YS, TAO, J, TARASEVICH, B and BUCHKO, G

Pacific Northwest National Laboratory, Richland, WA 99354

Email: wendy.shaw@pnnl.gov

OBJECTIVES: Full-length amelogenin is necessary for proper enamel formation, but very little is understood at a mechanistic level about how amelogenin controls crystal growth. Protein structure (secondary, tertiary, and quaternary) is thought to play a key role in the function of amelogenin in enamel formation, and our group has started to provide quantitative insight into the structure of amelogenin in solution and bound to hydroxyapatite (HAP). The wildtype protein has a common lack of structure in solution[1,2] and in the nanosphere, but becomes structured when bound to HAP along with a reduction in flexibility.[2] The quaternary structure upon binding to HAP is significantly different than in solution[3]. The goal of these studies is to understand the secondary, tertiary, and quaternary structures of two naturally occurring mutants, T21I and P41T, to correlate structure and function in enamel development.

METHODS: Using solution and solid state NMR and AFM, the secondary, tertiary, and quaternary structure of two naturally occurring *full-length* amelogenin mutants will be compared in solution, in the nanosphere and bound to HAP.

RESULTS: The structural flexibility is changed in solution as a function of mutation, as well as binding to the surface. Preliminary data showing an intermolecular protein-protein interaction will also be shown. The quaternary structure also changes significantly for the single site mutants, a change that may be critical to amelogenin's function.

CONCLUSION: The application of NMR and AFM techniques to allow the investigation of proteins of >60 residues represents a major advancement for amelogenin specifically and biomineralization proteins in general. The differences as a function of single site mutation suggest that these mutations are critical in controlling structure, and/or protein-protein or protein-HAP interactions.

ACKNOWLEDGEMENTS: This work is funded by NIDCR, NIH, grant #DE-015347

REFERENCES: [1]Buchko, G. W.; Tarasevich, B. J.; Bekhazi, J.; Snead, M. L.; Shaw, W. J. *Biochemistry* 2008, *47*, 13215-13222; [2]Lu, J.; Xu, Y. S.; Buchko, G. W.; Shaw, W. J. *Journal of Dental Research* 2013, *92*, 1000-1004; [3]Tao, J.; Buchko, G. W.; Shaw, W. J.; Yoreo, J. J. D.; Tarasevich, B. J. *Langmuir* 2015, *31*, 10451-10460

DISCUSSION:

Janet Moradian-Oldak (Southern California): Is the perceived protein-mineral binding energy actually influenced by a contribution from protein-protein binding? The mutation could make the proteins more aggregative so they form layers on the apatite.

Wendy Shaw (Pacific Northwest National Laboratory): I would say that both interactions have increased. Protein-protein binding interaction has increased and the protein-hydroxyapatite interaction has increased, so both of those binding energies have gone up.

Janet Moradian-Oldak (Southern California): I was just wondering if in your calculations if these two distinct interactions are completely independent parameters for calculating energy of binding?

Wendy Shaw (Pacific Northwest National Laboratory): Based on the data we have, it's not clear what amino acids are contributing to binding so I'm hoping we'll be able to get that data from solid state NMR.

#72 INTRACELLULAR PROTEIN INTERACTIONS AS AETIOLOGICAL FACTORS IN AMELOGENESIS IMPERFECTA?

GABE, CM*, BROOKES, SJ, MYERS, SL, KINGSWELL NJ and KIRKHAM, J

Division of Oral Biology, School of Dentistry, University of Leeds, Leeds, LS9 7TF, UK

Email: dncmg@leeds.ac.uk

OBJECTIVES : Mutations in amelogenin are driving factors in some cases of amelogenesis imperfecta (AI). A Y64H point mutation in murine amelogenin, phenocopying the human P70T mutation, is associated with abnormal intracellular amelogenin accumulation, endoplasmic reticulum (ER) stress and ameloblast apoptosis driven by the unfolded protein response (UPR). We hypothesise that the Y64H mutation causes intracellular amelogenin aggregation leading to ER stress. Our aim here was to investigate the effect of the mutation on amelogenin interactions *in vitro*.

METHODS: Recombinant (r-) WT and Y64H His-tagged amelogenins were expressed in *E. coli* transfected with pET28/AMELX WT or pET28/AMELX MUT vectors. r-Amelogenin was extracted with 3% v/v acetic acid and desalted. His-tag cleavage was carried out followed by FITC labelling. The r-amelogenin was further purified using preparative SDS-PAGE and desalted. Solid phase binding assays were carried out to compare binding of solubilised FITC labelled WT or mutant r-amelogenin to unlabelled immobilised WT or mutant r-amelogenin.

RESULTS: Acid extraction generated an enriched r-amelogenin fraction eliminating the need for His-tag purification. Preparative SDS-PAGE produced highly purified FITC labelled r-amelogenin. Binding assays suggested that labelled Y64H mutant r-amelogenin is significantly more aggregative than WT by a factor of 3 ($p<0.05$).

CONCLUSIONS: A convenient and efficient methodology has been developed to produce recombinant WT and Y64H amelogenin. Handling steps have been reduced to a minimum increasing the yield to milligram amounts per run. Solid phase binding data indicated that the Y64H mutation can enhance amelogenin-amelogenin interactions, which may explain the observed intracellular accumulation of amelogenin and subsequent ER stress characteristic of AI in affected mice. The data suggest that pharmaceutical inhibition of abnormal protein aggregation, or upregulation of chaperones (foldases), may offer therapeutic benefit in cases of AI driven by abnormal intracellular protein aggregation.

DISCUSSION:

Ariane Berdal (Paris): Do you think that the intracellular accumulation of protein could be important for the transition from the secretion stage to the maturation stage because at transition stage the cells die. Could it be that you recapitulate something that is occurring during amelogenesis?

Claire Gabe (Leeds): Mutant amelogenin tends to aggregate. The ER attempts to manage this by for example, increasing folding capacity. Initially, the UPR signalling cascades try to rescue the cell but if that doesn't work then the UPR triggers apoptosis.

Steve Brookes (Leeds): Just a quick comment, the apoptosis starts in secretion; that’s the key thing.

#73 PHOSPHORYLATION INFLUENCES THE SECONDARY STRUCTURE OF THE LEUCINE-RICH AMELOGENIN PEPTIDE

YAMAZAKI, H[1,2]*, BENIASH, E[3], YAMAKOSHI, Y[4], SIMMER, JP[5], and MARGOLIS, HC[1,2]

[1]Center for Biomineralization, Department of Applied Oral Sciences, The Forsyth Institute, Cambridge, MA, USA; [2]Department of Developmental Biology, Harvard School of Dental Medicine, Boston, MA, USA; [3]Department of Oral Biology, Center for Craniofacial Regeneration, McGowan Institute for Regenerative Medicine, University of Pittsburgh, Pittsburgh, PA, USA; [4]Department of Biochemistry and Molecular Biology, School of Dental Medicine, Tsurumi University, Yokohama, Japan; [5]Department of Biologic and Materials Sciences, University of Michigan School of Dentistry, Ann Arbor, MI, USA

Email: HYamazaki@forsyth.org

Previously, we showed that serine-16 phosphorylation in native full-length porcine amelogenin (P173) and the Leucine-Rich Amelogenin Peptide (LRAP(+P)), an alternative amelogenin splice product, affects protein assembly and mineralization *in vitro*. Notably, P173 and LRAP(+P) stabilize amorphous calcium phosphate (ACP) and inhibit hydroxyapatite (HA) formation, while non-phosphorylated counterparts (rP172, LRAP(-P)) guide the growth of ordered bundles of HA crystals.

OBJECTIVE: To better understand how protein phosphorylation affects mineralization by determining its influence on secondary structures of amelogenin in the absence / presence of calcium and relevant mineral phases (HA – a prototype for enamel crystals; ACP – enamel crystal precursor).

METHODS: Solutions (5-15 mg/mL) of LRAP(+P), LRAP(-P), P173 and rP172 were prepared with / without $CaCl_2$ (7.5 mM) at pH 7.4. FTIR spectra of each solution were obtained using attenuated total reflectance (n= 3). Amide-I peaks were analyzed to provide secondary structure information. Secondary structures of LRAP(+P) and LRAP(-P) were similarly assessed following incubation (4 h) with suspensions of HA and pyrophosphate-stabilized ACP.

RESULTS: Amide-I spectra of LRAP(-P) and LRAP(+P) were found to be distinct from each other in all cases. Spectra analyses showed that LRAP(-P) is comprised mostly of random coil and β-sheet structures, while LRAP(+P) exhibits more β-sheet and α-helix structures, with little random coil. With added Ca, the random coil content increased in LRAP(-P), while LRAP(+P) exhibited a decrease in α-helix components. Similar tendencies were observed in the spectra and secondary structures of P173 and rP172, but to a lesser extent. Incubation of LRAP(-P) with HA or ACP resulted in comparable increases in β-sheet structure. Notably, however, LRAP(+P) secondary structure was more affected by ACP, primarily showing an increase in β-sheet structure, compared to that observed with added HA.

CONCLUSIONS: Findings suggest that phosphorylation of serine-16 in amelogenin induces unique secondary structure changes that may enhance its functional capacity to stabilize ACP during amelogenesis. Supported by NIDCR grant DE-023091 (HCM).

DISCUSSION:

Unknown: My question regards cell culture. When you analyse the biological function of LRAP, did the phosphorylation state influence the differentiation of cells?

Hajime Yamazaki (Forsyth): In cell culture? No I haven't and it would be difficult to measure using FTIR because, as you say, there will be a mixture of phosphorylated and non-phosphorylated proteins.

#74 AMELOGENIN PHOSPHORYLATION IS ESSENTIAL FOR TOOTH ENAMEL FORMATION

SHIN, N-Y[1,2], YAMAZAKI, H[1,2], MARGOLIS, SS[3], PUGACH, MK[2,4], SIMMER, JP[5], BENIASH, E[6] and MARGOLIS, HC[1,2]*

[1]Center for Biomineralization, Department of Applied Oral Sciences, The Forsyth Institute, Cambridge, MA, USA; [2]Department of Developmental Biology, Harvard School of Dental Medicine, Boston, MA, USA; [3]Department of Biological Chemistry, The Johns Hopkins University School of Medicine, Baltimore, MD, USA; [4]Department of Mineralized Tissue Biology, The Forsyth Institute, Cambridge, MA, USA; [5]Department of Biologic and Materials Sciences, University of Michigan School of Dentistry, Ann Arbor, MI, USA; [6]Department of Oral Biology, Center for Craniofacial Regeneration, McGowan Institute for Regenerative Medicine, University of Pittsburgh, Pittsburgh, PA, USA

Email: hmargolis@forsyth.org

Our recent studies have demonstrated a unique ability of full-length amelogenin to form higher-order assemblies and regulate ordered mineralization *in vitro*, as seen in developing enamel. We have also found that phosphorylation of serine-16 greatly enhances the capacity of native amelogenins to stabilize nanoparticles of amorphous calcium phosphate (ACP), the first mineral phase in developing enamel, preventing hydroxyapatite (HA) crystal formation. These observations lead us to hypothesize that amelogenin phosphorylation is critical for proper enamel formation.

OBJECTIVE: To test this hypothesis *in vivo.*

METHODS: Working with a commercial laboratory, we generated an amelogenin knock-in (KI) mouse with a targeted serine-16 to alanine point mutation that is phosphorylation-defective. Teeth from KI, Het and WT littermates were assessed using SEM, TEM, immunohistochemistry (IHC) and μCT.

RESULTS: KI, Het and WT littermates were readily generated, although KI mice required a soft diet. Western blot and IHC findings confirmed the absence of amelogenin phosphorylation. In sharp contrast to the WT, KI enamel was brittle and exhibited a marked phenotype in both secretory and maturation stages. Mature KI enamel showed a loss of decussating prism patterns and numerous surface defects (nodules). TEM analyses of secretory and early maturation stages indicate that KI enamel (incisor) is comprised of discontinuous short bundles of aligned HA-like crystals, unlike WT enamel that exhibits extremely long and well-aligned enamel ribbons. Het enamel also appeared abnormal, with a variable mosaic appearance with portions of underlying and surface enamel reflecting both WT enamel and KI phenotypes. Based on μCT, KI and Het enamel were significantly under-mineralized, compared to WT enamel. Preliminary evidence suggests that ACP->HA transformation seen in each phenotype takes place faster in KI enamel.

CONCLUSION: Amelogenin serine-16 phosphorylation is essential for proper enamel formation and, in part, plays a critical role in regulating the appositional growth of initial enamel ribbons.

ACKNOWLEDGEMENTS: Supported by NIDCR grant DE-023425 (HCM)

No Questions

#75 ARE INTERACTIONS BETWEEN JUNCTIONAL EPITHELIUM PROTEINS KEY TO DENTOGINGIVAL ATTACHMENT?

HOLCROFT, J, SAN MIGUEL, S, GANSS, B*

Faculty of Dentistry, University of Toronto, Toronto, ON M5S3E2, CANADA

Email: b.ganss@utoronto.ca

The molecular composition of the layer between gingival epithelial cells and tooth mineral at the junctional epithelium (JE) is poorly defined, with only laminin 5 (LAM332) consistently localized at this interface. More recently, several enamel-derived proteins such as ameloblastin (AMBN), amelotin (AMTN), odontogenic, ameloblast-associated (ODAM), follicular dendritic cell secreted protein (FDCSP) and secreted calcium binding phosphoprotein, rich in proline and glutamine (SCPP-PQ1) have been found in the JE. AMTN and ODAM have been shown to interact strongly in vitro.

OBJECTIVE: To determine whether AMBN, AMTN, ODAM, FDCSP, SCPP-PQ1 and LAM332 subunits interact and to analyze the affinities and kinetics of such interactions in vitro.

METHODS: Coding sequences for all proteins investigated were cloned into bait and prey vectors of the high stringency Yeast-two-hybrid Matchmaker® Gold system and protein interactions identified by growth and color conversion of yeast colonies. Recombinant proteins were expressed in *E. coli* and affinity purified. Surface Plasmon Resonance (SPR) measurements were conducted to determine binding affinities and kinetics. Immunogold Transmission Electron Microscopy (TEM) was used to determine the ultrastructural localization of proteins at the cell-mineral interface of the JE.

RESULTS: AMTN, ODAM and FDCSP, as well as LAMC2, were localized at the internal basal lamina of the JE in human tissues. Select protein interactions between AMBN, AMTN, FDCSP, SCPP-PQ1, LAMB3 and LAMC2 were confirmed by yeast-two-hybrid interaction analyses. The affinities between most proteins were found to be in the 1 x 10^{-7} M range.

CONCLUSION: The presence of the enamel-derived proteins AMBN, AMTN, ODAM, FDCSP, SCPP-PQ1 and LAMB3 and LAMC2, as well their select interactions, suggest that these proteins are functional constituents of a specialized basal lamina-like interface that mediates the adhesion between gingival epithelial cells and tooth mineral at the junctional epithelium.

<u>DISCUSSION</u>:

Steve Brookes (Leeds): Is there any indication that the enamel proteases are expressed in junctional epithelium?

Bernard Ganss (Toronto): Not that I'm aware of, not in the junctional epithelium. I don't think anyone has looked at that yet. I think it's all limited to the bacterial protease.

Jim Simmer (Michigan): We looked for KLK4 and didn't see KLK4.

#76 INVESTIGATING WDR72'S FUNCTION IN PROTEIN REMOVAL DURING ENAMEL MATURATION STAGE

KATSURA, KA*, ZHU, J, NAKANO, Y, ZHANG, Y, AND DEN BESTEN, P

School of Dentistry and Department of Oral and Craniofacial Sciences, University of California at San Francisco, San Francisco, CA 94143, US

Email: Kei.Katsura@ucsf.edu

OBJECTIVES: Our overall aim is to determine the function of WDR72 in enamel mineralization. We previously identified a human WDR72 mutation and characterized a *Wdr72* knockout mouse model, both showing hypomineralized and hypomaturation enamel phenotypes of Amelogenesis Imperfecta. Ours and other published works have demonstrated that loss of WDR72 leads to retained protein in the enamel matrix, which prevents the continuous growth of the enamel crystals. This retention of matrix proteins in the enamel space could be the result of either a defect in matrix protein hydrolysis or a defect in protein removal from the mineralizing matrix. To further direct our studies of the role of WDR72 in enamel formation, we investigated both of these possibilities.

METHODS: Expression of KLK4 mRNA in $Wdr72^{+/+}$, $Wdr72^{+/-}$ and $Wdr72^{-/-}$ micro-dissected maturation stage ameloblasts was compared by qPCR. Whole mandibular incisors and P14 molars were standardized by weight and demineralized in 0.5 M acetic acid overnight following homogenization. Samples were assayed for KLK4 proteolytic activity using a KLK4-specific quencher peptide and analyzed using a fluorescent plate reader. To determine the potential role of WDR72 in ameloblast-mediated endocytosis of proteins the enamel matrix, mice were injected with HRP and relative uptake into $Wdr72^{+/+}$ *and* $Wdr72^{-/-}$ maturation stage ameloblasts was compared using confocal microscopy.

RESULTS: KLK4 mRNA expression and proteinase activity were not significantly different among $Wdr72^{+/+}$, $Wdr72^{+/-}$ and $Wdr72^{-/-}$ mice. HRP uptake was reduced at the apical ends of $Wdr72^{-/-}$ maturation stage ameloblasts with no obvious effect in secretory ameloblasts.

CONCLUSIONS: Our findings support WDR72 as a regulator of enamel matrix removal and further suggest a role in endocytic processing of enamel matrix proteins. Reduced HRP uptake in $Wdr72^{-/-}$ maturation-stage ameloblasts coupled with no significant difference in KLK4 activity point to WDR72's function in reabsorption of enamel matrix proteins through endocytosis.

No Questions

PANEL DISCUSSION:

Steve Brookes (Leeds): This question relates to serine-16 in amelogenin. Have we any idea what the pK of the phosphate group is? Is there any inductive effect from the surrounding residues? Is it negatively charged at physiological pH?

Henry Margolis (Forsyth): I assume it is negatively charged. Our FTIR studies seem to show neutralisation with calcium binding or with the addition of protons. I don't know what the pK is; there are a couple of calculation programmes that suggest it is negatively charged.

Steve Brookes (Leeds): When you replace serine-16 with alanine, can you be sure that you are looking at the effect of loss of phosphorylation, rather than a gain of toxicity from the alanine? Could you replace phosphoserine with a negatively charged amino acid, e.g. aspartic acid, rather than replacement with uncharged alanine?

Henry Margolis (Forsyth): Yes that is one approach. We've done a lot of comparative work with non-phosphorylated serine in which phosphate is replaced with a hydroxyl group. We are planning to synthesise the alanine substituted protein so we can double check that. It may have an effect, but I don't know at this point.

Mike Hubbard (Melbourne): Congratulations on producing a mouse expressing non-phosphorylated amelogenin. What is known about the kinases that phosphorylate serine-16 and how practical would it be to phosphorylate recombinant amelogenin so that everybody works with the naturally relevant protein?

Henry Margolis (Forsyth): If we'd have been working with the native phosphorylated proteins then that would save a lot of work! Kinases are very interesting, a lot of people are working with them, so it is something that we're interested in. Is there an AI family where the phosphorylation is lacking? The phosphorylation site is highly conserved so it's important, and its absence may be of importance pathologically.

Jim Simmer (Michigan): I have a comment about kinases and the FAM20 group. It might be possible to express these in *E. coli* concurrently with recombinant amelogenin and thereby phosphorylate serine-16. Really nice work on that serine-16 knock-out. I think there's a lot more work that needs to be done on showing the differences between the simple loss of the phosphate group and the complete loss of the protein. How much amelogenin function is retained in the absence of serine-16 phosphorylation? It looks very similar to a complete amelogenin knock-out. I'm wondering if this has ramifications for all the structure-function data we have acquired that is based on recombinant protein that doesn't have that phosphate? It would seem like an awful lot of amelogenin function is dependent upon phosphorylated serine-16.

Henry Margolis (Forsyth): I think serine-16 phosphorylation is very important. With the loss of the phosphate group in the mouse model there is an effect on the regulation of mineralisation and regulation of crystal shape and orientation similar to what we saw *in vitro*. I think we have to change some of our thinking when we say that recombinant amelogenin proteins do not stabilise ACP. There are concentration effects; we worked with rather dilute solutions of non-phosphorylated amelogenins *in vitro* and saw a weak stabilisation effect but *in vivo*, the concentration could be very much higher, possibly hundreds of milligrams per mL. A lot of biomineralisation proteins are phosphorylated, amelogenin has one phosphate, salivary proteins have two, osteopontin has many. It is very important, and it wasn't lost upon me looking at your recent data on the amelogenin knockout - a lot of functionality is tied in with the phosphorylation. Could there be other things going on? They're the things we're checking.

Ariane Berdal (Paris): Did you have a chance to immuno-label amelogenin in your mutants? Do you think that the amelogenins would not only affect enamel directly but also cause a loss of ameloblast polarity? Could loss of polarity cause the ectopic mineralisation you describe? We have also observed similar ectopic mineralisation in *Fam20a* and *Fam20c* mutants in which amelogenin phosphorylation would be affected.

Henry Margolis (Forsyth): The ameloblasts look normal during the secretory stage, but during the maturation stage they become very abnormal. I think the dysregulation of the mineralisation process initiates some sort of signal that affects cells and causes the loss of prism architecture. It could be other things, but the first thing that comes to mind is that cells are involved. It starts in the secretory stage, all of the cells look normal but they're recognising that mineralisation is abnormal.

Coin Robinson (Leeds): I am intrigued how the long wild type crystals compare to the lined up short fragments in the affected animals. Would you like to comment whether these fragments represent a repeating structure that is not normally present? Clearly, the process by which crystallites are formed is operating to a degree but it's very interrupted. There was an earlier paper by Dr Shaw about the early collapse of proteins on the surface of the crystal, it builds up then it's degraded, then it starts again.

Henry Margolis (Forsyth): We interpreted our data in terms of the loss of inhibition on crystal growth or nucleation. There is a lot of evidence that the crystal elongation process, not only in enamel, but in other biological systems, is through the non-classical crystal growth mechanism, involving the accumulation of pre-cursors or nucleation clusters or an accumulation of amorphous material. Previous reports show that crystal growth in non-biological systems occurs via the accumulation of an amorphous phase at the growing end of the crystals, which then transforms. In the absence of phosphorylated serine-16, I think such an amorphous phase transforms too quickly and the crystal growth process is slowed. Phosphorylated amelogenin, stabilises these amorphous phases to promote crystal elongation over starting new growth centres.

Jim Simmer (Michigan): I think it might be a little premature to draw some of these conclusions as those kinds of effects can be explained by the plain of section. Rods may appear very short because they're going in or out of the section. From our experience with the amelogenin null mouse, different section plains may give different impressions as to the length of the rods and crystal observed.

Henry Margolis (Forsyth): I agree and that's why we measured them. More work needs to be done, maybe some tomography, to verify the data but the wild type and the knock-ins are prepared in exactly the same way.

Olivier Duverger (Bethesda): I have a question for Bernhard. Rodents have continuously growing incisors and it is essential to maintain the junctional epithelium. Given the incisor is continually erupting, this must be an active process involving constant remodelling. For that reason, it is not wholly relevant to human junctional epithelium formation, but do you think the incisor model may provide more information on the dynamics of how the junctional epithelium is maintained both on the enamel and dentinal surfaces present in the incisor?

Bernhard Ganss (Toronto): We haven't looked at the junctional epithelium in incisors. You are correct; incisor junctional epithelium differs from the molar junctional epithelium in terms of both its dynamics due to the continuous eruption of the incisor, but also due to differences between lingual and buccal sides of the incisor. All the studies we have done to date were done in mouse and human molars.

Olivier Duverger (Bethesda): Do you think ameloblasts could undergo a final trans-differentiation and have a role related to the junctional epithelium? I don't think the ameloblasts just disappear as there would be a gap, or at least a transient gap, between the ameloblasts and the junctional epithelium, which would have to be "re-glued". Do you have any idea as to the eventual fate of the ameloblasts and whether they are incorporated into the junctional epithelium?

Bernhard Ganss (Toronto): There have been some studies looking at what percentage of ameloblasts undergo apoptosis and at what stage. Some of them do apoptose but the rest are shed. There has been a publication from a Japanese group earlier this year where lineage tracing confirmed that the junctional epithelium is mainly derived from the reduced enamel epithelium. I'm not sure as to the actual dynamics involved when the reduced enamel epithelium converts entirely into junctional epithelium.

Jim Simmer (Michigan): There have been a number of reports where specific amelogenin residues have been changed, leading in many cases to increased binding to the crystals and also increased protein-protein aggregation. One of the main properties of amelogenin, and the matrix in general, is that it's highly absorbable. It is a critical function of that matrix that it can be degraded and removed. One possible conclusion from amelogenin-mineral binding experiments is that there is a strong selection pressure dictating binding affinity. Binding cannot be too strong; perhaps akin to substrate

binding to an enzyme active site? Can we conclude from such studies that there may be an optimal binding affinity between amelogenin and the mineral?

Wendy Shaw (Pacific Northwest National Laboratory): I think that is a very good possibility. For me, it is hard to say whether it's merely binding strength that is directly affected by an amino acid substitution or whether there is a structural change associated with a substitution that blocks binding to the mineral. Being able to distinguish between those two things is critical and experiments can be designed to test this as we learn more about amelogenin structure when bound to the mineral surface.

Henry Margolis (Forsyth): There is an increase in binding in the mutated forms, but as Wendy said, conformation of protein on the surface is very important. We just published a paper showing that the phosphorylated amelogenin has a larger footprint on the apatite surface. Our interpretation of that data is that it makes amelogenin more effective in terms of crystal growth inhibition. I don't think it's the phosphate group *per se* that increases the inhibitory potential of amelogenin on crystal growth that but rather the effect of phosphorylation on amelogenin conformation once it binds to the apatite surface.

Janet Moradian-Oldak (Southern California): Amelogenin binding to mineral is a very dynamic process. For many years we believed it was the C-terminal domain that had a strong affinity for the mineral surface. Now we know that there are binding domains at the N-terminal. Let's not forget that MMP20 and KLK4 cleave the protein and can therefore control and manipulate the way amelogenin binds to the mineral.

Colin Robinson (Leeds): We shouldn't talk about these crystals as if they exhibit a uniform apatite surface. Jennifer Kirkham has shown very distinct charged domains along the crystal. If you look at the chemical composition from the inner to outer enamel the carbonate goes down four-fold and similarly, the magnesium goes down. The crystal surface is not uniform as the crystal chemistry depends on where the crystals originated. The generic information may be correct, but at some point you're going to have to look at crystals with more carbonate, more magnesium, and whether binding is related specifically to those charged domains.

Wendy Shaw (Pacific Northwest National Laboratory): Thanks Colin, that's a really good point. We've done some studies where we've observed that LRAP adopts specific secondary structures when bound to apatite containing various levels of carbonate. We do see a difference so I think it's critical to consider all of those things in our *in vitro* studies.

Sylvie Babajko (Paris): I have a question for Janet. Did you look at amelogenin co-localisation with enamelin or other enamel matrix proteins? Do you have a hypothesis that the protein-protein interactions that pre-determine matrix function initially occur inside the cell? Alternatively, knowing that translation is not really concomitant, do proteins interact following secretion into the extracellular matrix?

Janet Moradian-Oldak (Southern California): We have a paper from 2011 where we studied amelogenin co-localisation with enamelin. Within the limits of the techniques available to us, we did observe co-localisation; especially at the very early stage, close to Tomes' process. This is not surprising, as we know in some cases that these proteins are secreted together, but we haven't done any systematic analyses using FRET to look at interaction with other proteins in the cell. These proteins are going to be together and they are sometimes secreted together. What question are you asking regarding intracellular events?

Sylvie Babajko (Paris): The translation of different mRNAs is not necessarily concomitant, so how do you explain why different matrix proteins are present in the same vesicles. At what point do they meet and interact?

Janet Moradian-Oldak (Southern California): Ameloblastin and amelogenin are co-localised together in the secretory vesicles. Is nature being efficient by using the same bus to transport two proteins? Maybe this is a chaperone mechanism to avoid premature aggregation or maybe there is some other functional reason. Regarding protein co-localization or interaction in the extracellular matrix: maybe everything starts with off as a heteromolecular assembly and with degradation, component molecules get re-distributed. For example, the C-terminal of ameloblastin does its job, and then ameloblastin is quickly degraded leaving the insoluble N-terminals that concentrate together around the prism boundaries.

Mike Hubbard (Melbourne): Question for Leeds. Having some time ago suggested that the endoplasmic reticulum's involved in transporting calcium across the epithelium, I'm naturally interested in the UPR story you have bought to the table. How restricted do you think this is to the genotype you've got; might the UPR be a feature in other mutations and besides the effects on protein secretion, do you think calcium transport might also be disrupted? In cell biology, the UPR is not necessarily a bad thing. The UPR is beneficial in secretory cells and its negative effects impact if stress becomes excessive. Have you seen a less severe phenotype in those mutants cells that are perhaps under less load, for example the second molar that has got thinner enamel or cells in the cervical regions that produce less enamel?

Claire Gabe (Leeds): I think that even the less stressed cells use the UPR and maximise the folding capacity of the ER to help maintain the secretory pathway. With the mutation, the cells are overstressed to the point that the folding mechanisms are no longer sufficient. The kinetics of chaperone binding and management of the secretory load might be affected leading to apoptosis.

Steve Brookes (Leeds): The molars in our heterozygous amelogenin mutants are massively affected, so I think they're suffering ER stress too. You're quite right that wild type ameloblasts normally activate the UPR. John Bartlett's lab showed that the UPR was active in secretory ameloblasts and then was down-regulated again in maturation, when the secretory load and ER stress was reduced.

Your comment on calcium is highly relevant because affected cells fail to fully mineralise the enamel. They may produce a matrix but it never mineralises, so calcium transport and calcium handling in general may well be affected too.

Mike Hubbard (Melbourne): This could be relevant to phospho-amelogenin, as phospho-amelogenin could be affecting the amount of calcium in the secretory vesicle, so all these things are nicely integrated.

John Bartlett (Ohio): I believe the UPR was first discovered in B cells, which secrete a huge amount of antibodies and no one considers these cells stressed. The UPR just helps those massive amounts of proteins get folded properly and the antibodies put together properly so they can be secreted in the correct way. I think that happens to the ameloblasts in the secretory stage.

Jennifer Kirkham (Leeds): Just to pick up on Mike's comment about the beneficial effects of the UPR. We've published on mouse molars in amelogenin mutants and they were badly affected. We also did quite a bit of work looking at some of the beneficial aspects of the UPR. The cells (affected by the mutation) are okay at first, they're surviving thanks to the UPR. You can quantitate the effect of the UPR: measure the amount of ER, as the ER itself becomes more voluminous under stress, or you can look at the way the UPR down-regulates protein secretion in order to maintain secretory homeostasis. We find that the initial response works to help cells maintain some function. It appears to us, and this is where we speculate, that once the burden becomes too great, then there is a switch in the signalling pathway towards apoptosis, but at first we see an up-regulation of beneficial aspects related to the response.

Wendy Shaw (Pacific Northwest National Laboratory): I have a question for Claire. Earlier this week Tomas Wald explained how he thought the tri-tyrosyl motif might affect amelogenin self-assembly in ameloblasts and I'm curious whether you looked at that in your mutation?

Claire Gabe (Leeds): We haven't looked at that specifically but it is an interesting question.

Steve Brookes (Leeds): Before Claire joined us, we did try looking at amelogenin binding with *N*-acetylglucosamine, which reportedly binds via the tri-tyrosyl motif, but unfortunately we didn't observe binding with either wild type or mutant amelogenin.
